# Direct catalytic asymmetric and *anti*-selective vinylogous addition of butenolides to chromones†

**DOI:** 10.1039/d0sc01914c

**Published:** 2020-06-22

**Authors:** Jin Cui, Naoya Kumagai, Takumi Watanabe, Masakatsu Shibasaki

**Affiliations:** Institute of Microbial Chemistry (BIKAKEN) Tokyo 141-0021 Japan twatanabe@bikaken.or.jp mshibasa@bikaken.or.jp

## Abstract

An *anti*-selective catalytic asymmetric Michael-type vinylogous addition of β,γ-butenolides to chromones was developed. The catalyst system developed herein is characterized by tuning of the steric and electronic effects using a proper Biphep-type chiral ligand to invert the diastereoselection, and improvement of the catalyst turnover by a coordinative phenolic additive. The catalytic protocol renders potentially biologically active natural product analogs accessible in good yield with moderate diastereoselectivity and high enantiomeric purity, mostly greater than 99% ee.

## Abstract 2

An *anti*-selective catalytic asymmetric Michael-type vinylogous addition of β,γ-butenolides to chromones was developed.

## Graphical abstract

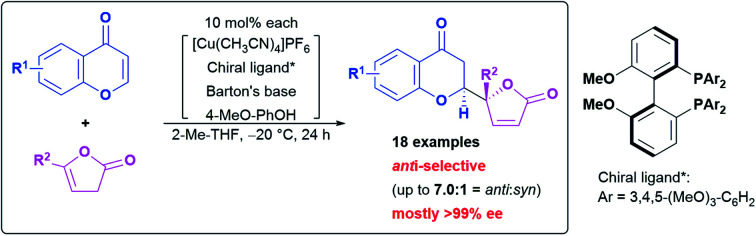

## Introduction

Well-designed asymmetric transformations under reagent-controlled conditions have revolutionized C–C bond formation with the desired absolute configuration.^1^ Moreover, catalytic asymmetric reactions in the modern era are in demand because of their sustainability; direct asymmetric catalytic reactions that do not require activation of the substrate(s) by derivatization are ideal processes in terms of atom-economy.^2^ Our research group has devoted tremendous effort toward developing these valuable transformations, including aldol- and Michael-type catalytic asymmetric reactions. In fact, we reported the first catalytic asymmetric aldol reaction in 1992,^3^ a catalytic asymmetric nitroaldol reaction promoted by the LaLi_3_tris(binaphthoxide) (LLB) catalyst comprising BINOL, La, and Li (3 : 1 : 3 complex). Subsequently, we disclosed the first example of a direct catalytic asymmetric aldol reaction of simple ketones in 1997.^4^ For conjugate additions, a La–Na–BINOL-catalyzed^5^ asymmetric Michael reaction was published in 1995, followed by the development of Al–Li–BINOL^6^ and La-linked-BINOL^7^ catalyzed versions. Since then, direct catalytic asymmetric aldol and Michael strategies have been applied to various substrates to attain universal applicability; recent endeavors indicate that even notoriously tough pronucleophiles, such as amides, can be tamed by selective activation toward deprotonation through the coordination of chiral copper species to a tactically designed amide-substructure.^8^

Chiral γ-butenolide units that feature a five-membered γ-lactone with unsaturation at α,β-carbon atoms are ubiquitous in natural products and biologically active compounds.^9^ The direct utilization of the nonactivated α,β- and β,γ-unsaturated butyrolactones as pronucleophiles instead of 2-siloxyfurans avoids the generation of silyl by-products and the preactivation of butenolides, paving the way to optically active butenolide-containing products in an atom-economical manner.^10,11^ The direct use of γ-butenolide in catalytic asymmetric vinylogous conjugate addition to chromones is a straightforward approach to highly appealing biologically privileged architectures which was pioneered by Trost *et al.*^10^ The reported dinuclear zinc-ProPhenol-catalyzed transformation proceeds with high diastereo- and enantioselectivity with α,β- and β,γ-butenolides and a wide variety of chromones to afford natural product-like scaffold, in which the *syn*-configuration predominated as the newly formed connectivity (*vide infra*).

**Fig. 1:**
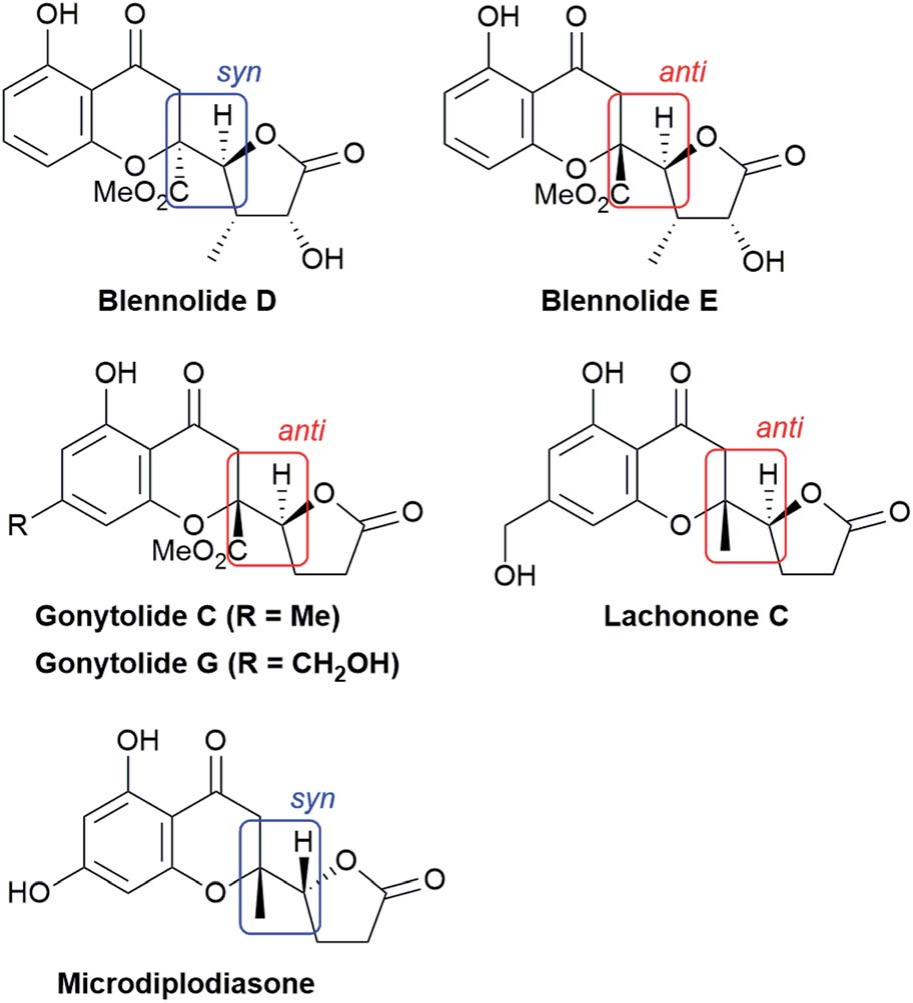
Structure of chromanone lactone natural products.

**Fig. 2:**
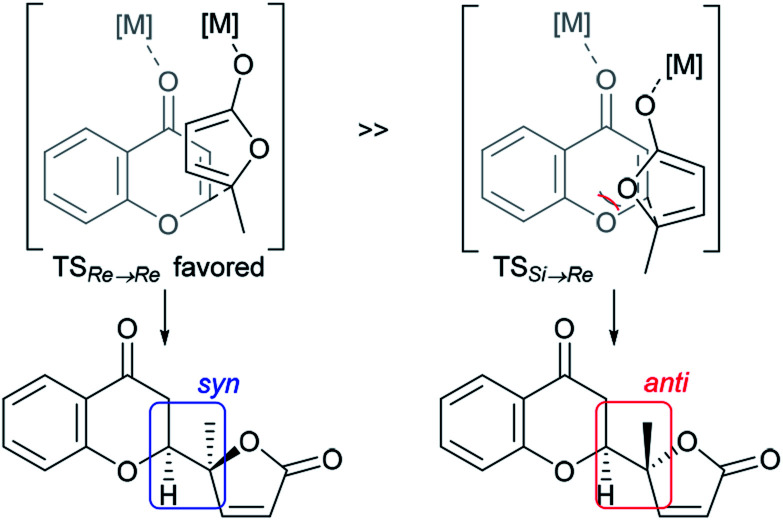
Rationale for intrinsic *syn*-selectivity of vinylogous addition of butenolide to chromone (ref. 10).

The structural features of the products share characteristics of the core components of the chromanone lactone natural products depicted in Fig. 1, including blennolides D and E, microdiplodiasone, gonytolides C and G, and lachnone C.^9^ Two of the natural products mentioned above have a *syn*-stereochemistry between the chromanone core and the butanolide-derived 5-membered lactone, but natural products with an *anti*-configuration are more abundant. Thus, an *anti*-selective version of the addition reaction of butenolides to chromanone is in high demand. In fact, in the above-mentioned paper, Trost stated that “we believe that the diastereoselectivity is a feature inherent to this type of transformation” in terms of the stereochemical outcome. Fig. 2 illustrates the predominant formation of *syn*-products attributed to repulsion of the lone pairs embedded in the chromone and butenolide cores. As Trost points out, the same tendency is observed in the cycloaddition reaction of siloxyfurans and benzopyryliums reported by Porco and co-workers.^12^ In the present report, we disclose a direct catalytic asymmetric vinylogous Michael-type reaction of chromones and β,γ-butenolides to afford *anti*-adducts with almost complete enantioselectivity using a newly developed catalyst system.

## Results and discussion

The reaction conditions were first screened using a simple chromone **1a** and α-angelica lactone **2a** treated with [Cu(CH_3_CN)]PF_6_, a cationic copper(i) source, chiral ligand, and Brønsted base (10 mol% each) at a fixed temperature (0 °C) in THF (Table 1). When (*R*)-tolyl-BINAP (**L1**) was utilized as a bidentate phosphine ligand with DBU as a base, the inherent *syn*-selectivity was confirmed (entry 1). Changing the ligand to (*R*)-Garphos (**L2**, entry 2) afforded more or less the same result as shown in entry 1 (*anti* : *syn* = 1 : 5.7 in entry 1, and 1 : 6.7 in entry 2). The enantiomeric excess of the major product (*syn*-isomer) was at best around 30% (entry 2), and conversion was equally unsatisfactory (only 2 turnovers). The chiral ligands, including (*R*,*R*p)-Walphos- (**L3**) and (*R*,*S*)-JosiPhos-type (**L4**) ligands, and (*R*,*R*)-Ph-BPE (**L5**) gave the *syn*-products, exclusively, with a meager chemical yield (entries 3–5). The catalyst system involving a (*R*,*R*p)-Taniaphos-type ligand (**L6**) resulted in almost no reaction (entry 6). Subsequently, we incorporated Biphep-type biphenyl-based chiral ligands. Our first selection was unfortunately fruitless; the **L7** ligand with a 3,5-xylyl group at the phosphorus centers resulted in a similar yield to earlier (entrie 7). Surprisingly, changing the aryl substituents on the phosphorus to 3,4,5-(MeO)_3_-phenyl (**L8**) reverted the diastereoselectivity to *anti* : *syn* = 2.6 : 1 with excellent enantioselectivity (>99% ee) of the *anti*-product^13^ (89% combined NMR yield of *anti*- and *syn*-isomers, entry 8). Decreasing the temperature to −40 °C improved the diastereoselectivity up to 3.5 : 1 without any loss of enantiopurity (>99% ee), but the chemical yield was reduced to 65% (entry 9). The choice of the Brønsted base was also critical to the reaction outcome. In fact, the use of triethylamine (Et_3_N) instead of DBU exhibited a detrimental effect on conversion, even at 0 °C (29%, entry 10). Tetramethylguanidine (TMG) slightly increased the yield compared to DBU (72%, entry 11) with the same diastereomeric ratio (3.5 : 1). Barton's base dramatically improved the conversion to 97%, but the diastereoselectivity was compromised (2.0 : 1, entry 12). Continuing with Barton's base, the solvent effect was thoroughly investigated. CH_2_Cl_2_, toluene, and dimethylformamide (DMF) showed no beneficial effect on *anti*-selectivity (2.1 : 1 at best, entries 13–15), whereas 2-Me-THF improved the diastereoselectivity (*anti* : *syn* = 3.5 : 1; entry 16). In each case, the enantiomeric ratio remained at an almost perfect level (>99% ee). The use of DBU in 2-Me-THF improved the diastereoselectivity to 4.1 : 1, but the conversion was unsatisfactory (60%, entry 17). Increasing the amount of DBU (20 mol%) negatively affected the conversion (40%) and diastereomeric ratio (2.0 : 1, entry 18).

**Table 1:** Optimization and screening of chiral ligands, Brønsted bases and solvents for the direct vinylogous addition of butenolide to chromone

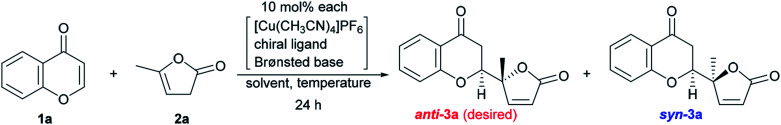									
Entry	Ligand		Brønsted base	Solvent	Temperature (°C)	Yield^a^ (%)	dr (*anti* : *syn*)	ee [%] (major isomer)	ee [%] (minor isomer)
1		**L1**	DBU	THF	0	22	1 : 5.7	93	−23
2		**L2**	DBU	THF	0	17	1 : 6.7	90	−30
3		**L3**	DBU	THF	0	7	1 : >19	ND	5
4		**L4**	DBU	THF	0	5	1 : >19	ND	23
5		**L5**	DBU	THF	0	4	1 : >19	ND	−12
6		**L6**	DBU	THF	0	trace	ND	ND	ND
7		**L7**	DBU	THF	0	24	1 : 6.7	92	−24
8		**L8**	DBU	THF	0	89	2.6 : 1	>99	33
9		**L8**	DBU	THF	−40	65	3.5 : 1	>99	48
10		**L8**	Et_3_N	THF	0	29	2.9 : 1	>99	33
11		**L8**	TMG	THF	−40	72	3.5 : 1	>99	50
12		**L8**	Barton's base	THF	−40	97	2.0 : 1	>99	44
13		**L8**	Barton's base	CH_2_Cl_2_	−40	95	1.3 : 1	>99	69
14		**L8**	Barton's base	Toluene	−40	94	2.1 : 1	>99	80
15		**L8**	Barton's base	DMF	−40	81	1 : 1	>99^c^	10
16		**L8**	Barton's base	2-Me-THF	−40	95	3.5 : 1	>99	44
17		**L8**	DBU	2-Me-THF	−40	60	4.1 : 1	>99	48
18		**L8**	DBU^b^	2-Me-THF	−40	40	2.0 : 1	>99	48
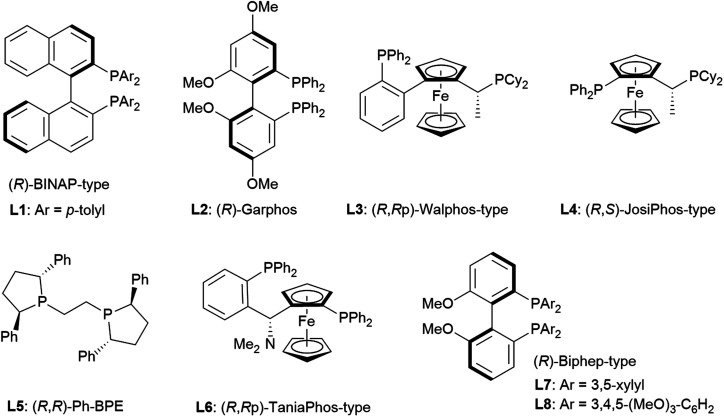									

a Combined NMR yield of both isomers.

b 20 mol% of DBU.

c ee of *anti*-isomer.

**Fig. 3:**
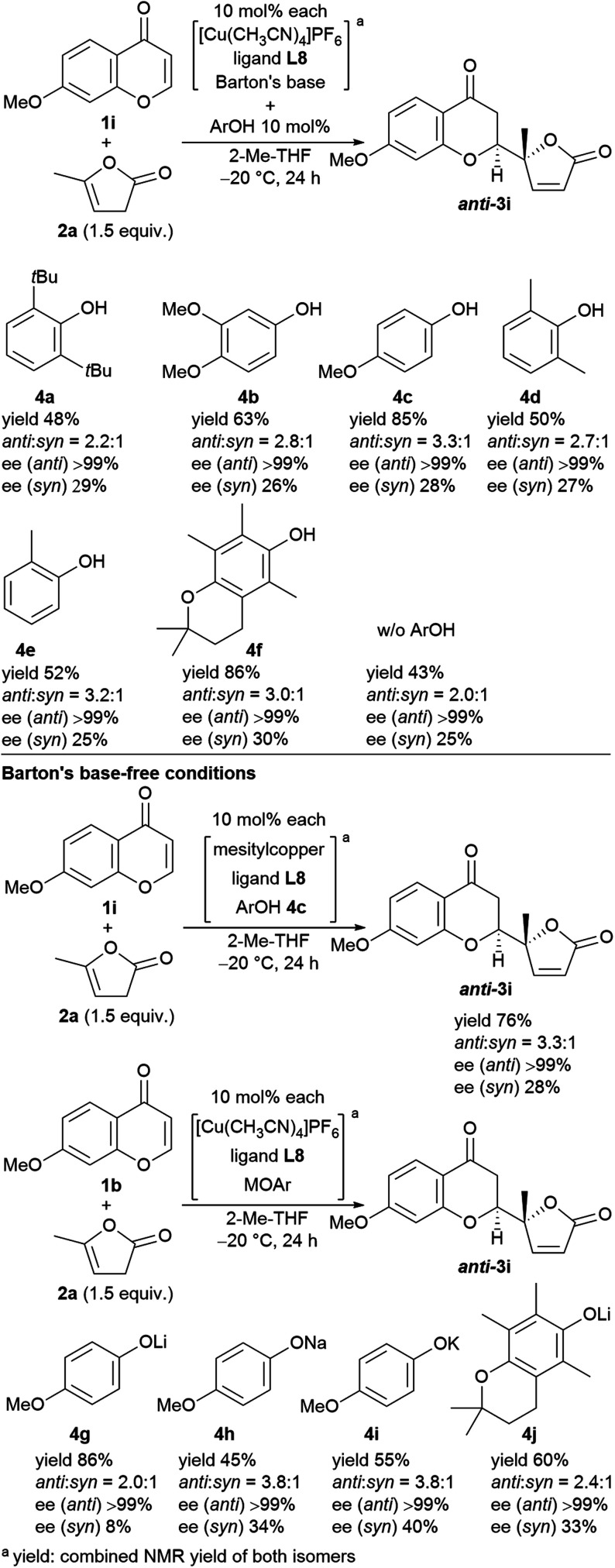
Screening of phenol additives for the direct vinylogous addition of butenolides to chromones and the reactions with Barton's base-free conditions.

We next undertook a study on substrate generality using the initially optimized reaction conditions shown in Table 1, entry 16, but only disappointing results were obtained. For example, the NMR yield of 6-MeO- (**3b**), 7-Me- (**3h**), and 7-MeO-products (**3i**) was low, around 30−40%, whereas 6-Br- (**3f**) and 7-AcO-adducts (**3j**) were afforded in a moderate yield with lower diastereoselectivity (2.3 : 1, and 2.1 : 1, respectively). At this stage, we began to fine-tune the reaction conditions using 7-MeO-chromone (**1i**) as the benchmark substrate with fixed parameters: temperature at −20 °C, reaction duration of 24 h, and 2-Me-THF as the solvent (43% yield, dr of 2.0 : 1, and >99% ee for major *anti*-products). Initially, the lithium alkoxide of 2,2,5,7,8-pentamethylchroman-6-ol (**4j**) was utilized, resulting in 60% NMR yield, 2.4 : 1 dr, and >99% ee for major *anti*-isomers (Fig. 3, bottom), which led us to investigate phenolic additives.^14^

**Fig. 4:**
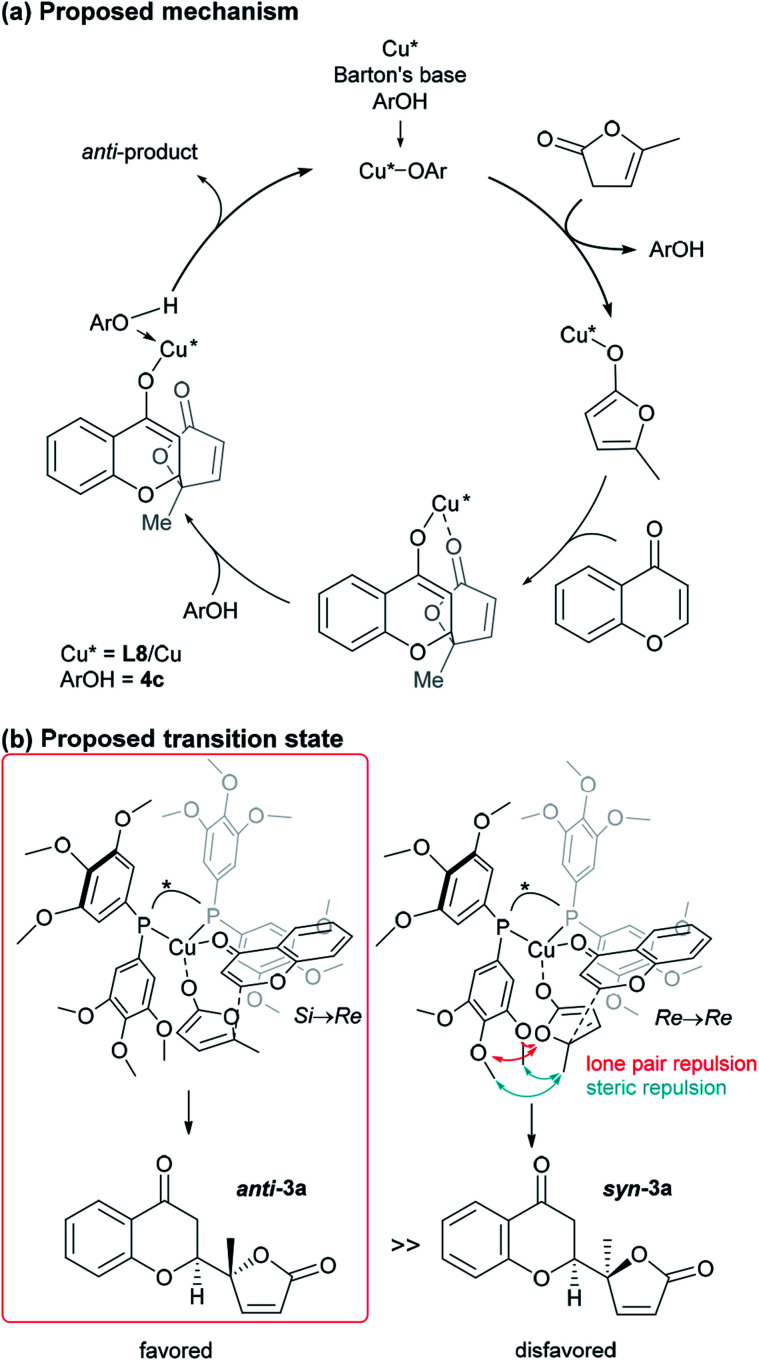
Proposed catalytic cycle and TS of the present vinylogous addition of butenolide to chromone.

Performing the reaction with five phenolic derivatives with varied substitution patterns (**4a–4e**) in the presence of Barton's base, as shown in Fig. 3, clearly indicate that additive **4c** was preferable for achieving high conversion up to 85% with satisfactory diastereoselectivity (3.3 : 1) and excellent enantioselectivity (>99%). The sterically hindered phenols (**4a**, **4d**, **4e**) bearing noncoordinative alkyl groups at the *ortho*-position yielded slight improvement of the conversion. Moreover, when using **4b** as the additive, a moderate conversion was observed. The noticeable effect of a phenolic additive that participates in the catalytic cycle to affect the reaction outcome was also documented in our previous aldol reaction using α-vinyl-appended thioamide and 7-azaindoline amide as substrates.^15^ In the present reaction, perturbation of the catalyst system by coordination of **4c** to the Cu^I^ center should be essential for the catalyst turnover from the Cu^I^–alkoxide (Fig. 4a). The deprotonation of butenolide by catalyst **L8**/Cu^I^-OAr **4c**, which was generated from **L8**/Cu^I^/Barton's base/**4c**, initiated the catalysis; the Cu^I^ dienolate formed a cyclic transition state with the incoming chromone en route to the *anti*-isomer *via* the coordination of **4c** to the Cu^I^ cation and subsequent protonation, followed by regeneration of the catalyst. On the other hand, the catalyst prepared with phenol **4f** provided comparable conversion but lower dr as compared with the reaction with **4c**. The electron-rich nature of **4f** corresponding to the high basicity of the conjugate base (Cu^I^–OAr) is crucial to promote the reaction. This electronic feature compensates for its reduced coordination, caused by the *ortho*-substituted methyl groups, with Cu^I^ cation from the Cu^I^–alkoxide intermediate.

Next, we revisited Barton's base-free conditions, but did not obtain superior results (Fig. 3, bottom). A basic copper source, MesCu, decreased the conversion to 76%. Both sodium- (**4h**) and potassium 4-methoxyphenoxide (**4i**) exhibited a good diastereoselectivity of 3.8 : 1, but the turnover was limited. The lithium congener (**4g**) drove the catalytic cycle efficiently (86% yield), but with only moderate diastereoselectivity (2.0 : 1). Throughout, the enantioselectivity remained at >99%.

At this point, the factors affecting the reversal of the diastereoselectivity was investigated. As shown in Fig. 2, the topology between chromone and butenolide upon their impending C–C bond formation disfavors the severe repulsion of two ring oxygens in the Si → Re transition state. In principle, this repulsive nonbonding interaction cannot be avoided simply by changing the structure of the substrates, which led Trost to express that this tendency is “inherent”. Intrigued by the change of preferred diastereoselection by **L7** and **L8**, we focused our attention on the substituent effect of the ancillary aromatic rings attached to the two phosphorous atoms of Biphep-type ligands (Table 2). The ligands **L9**, **L10** and **L7** exhibited a strong propensity to form *syn*-isomers (entry 1–3). The proportion of *anti*-isomers increased dramatically by introducing more sterically demanding substituents; two *tert*-butyl groups at *meta*-positions (**L11**) afforded a 1 : 1 mixture of *anti*- and *syn*-isomers (entry 4). This trend was maintained when another functional group (MeO) was introduced (**L12**); the *anti*/*syn* ratio increased to 1.4 : 1 (entry 5). We then examined the less sterically demanding ligand **L13** having MeO groups at *meta*-positions which resulted in a similar degree of *anti*-selectivity (1.7 : 1, entry 6) to that of entry 5. These results clearly indicate that steric bulkiness cannot be the only factor which govern diastereoselectivity, but electronic profiles are also critical. The trimethoxy substructure in **L8** provided the best *anti*-selectivity (entry 7).

**Table 2:** Effect of substituents on ancillary phenyl moieties of Biphep-type ligands

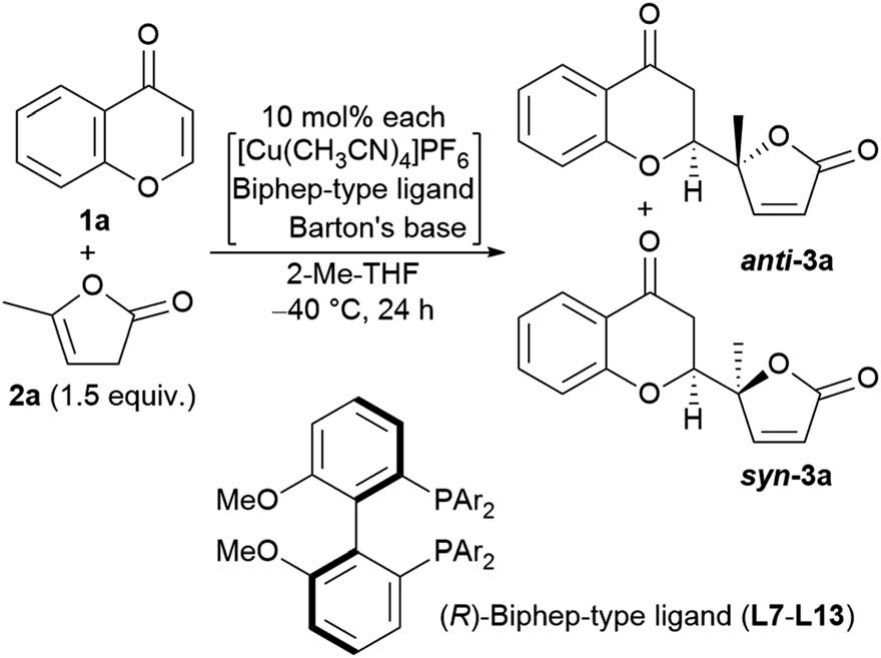				
Entry	Ligand	Yield^a^ [%], dr (*anti* : *syn*)	ee [%]	
			*anti*	*syn*
1	**L9**:	57	98	−37
	Ar = Ph	1 : 9.7		
2	**L10**:	43	97	−21
	Ar = *p*-tol	1 : 12		
3	**L7**:	47	98	−29
	Ar = 3,5-xylyl	1 : 7.5		
4	**L11**:	79	>99	53
	Ar = 3,5-*t*Bu_2_-Ph	1 : 1		
5	**L12**:	88	>99	80
	Ar = 3,5-*t*Bu_2_-4-MeO-Ph	1.4 : 1		
6	**L13**:	91	>99	35
	Ar = 3,5-(MeO)_2_-Ph	1.7 : 1		
7	**L8**:	>95	>99	46
	Ar = 3,4,5-(MeO)_3_-Ph	4.0 : 1		

a Combined NMR yield of both isomers.

Based on the results described above, we speculate that the present system takes advantage of the steric and electronic repulsion between the substituents at the ancillary benzene rings and the butenolide substrate to tame the “disfavored” transition state (Fig. 4b). Lower *anti*-selectivity of *tert*-butly-bearing ligands (**L11** and **L12**) compared to the less bulky ones (**L8** and **L13**) indicated that the steric effect is not determining factor for *anti*-selectivity. Introduction of the methoxy groups exerted electrostatic repulsion between the oxygens of the butenolide ring and ligand recognized in Re → Re, compared with those from the two ring oxygens in the Si → Re transition state. Thereby, the relative stability of the two transition states was flipped to fulfil the surprising stereochemical switch for the reaction course.^16^

**Fig. 5:**
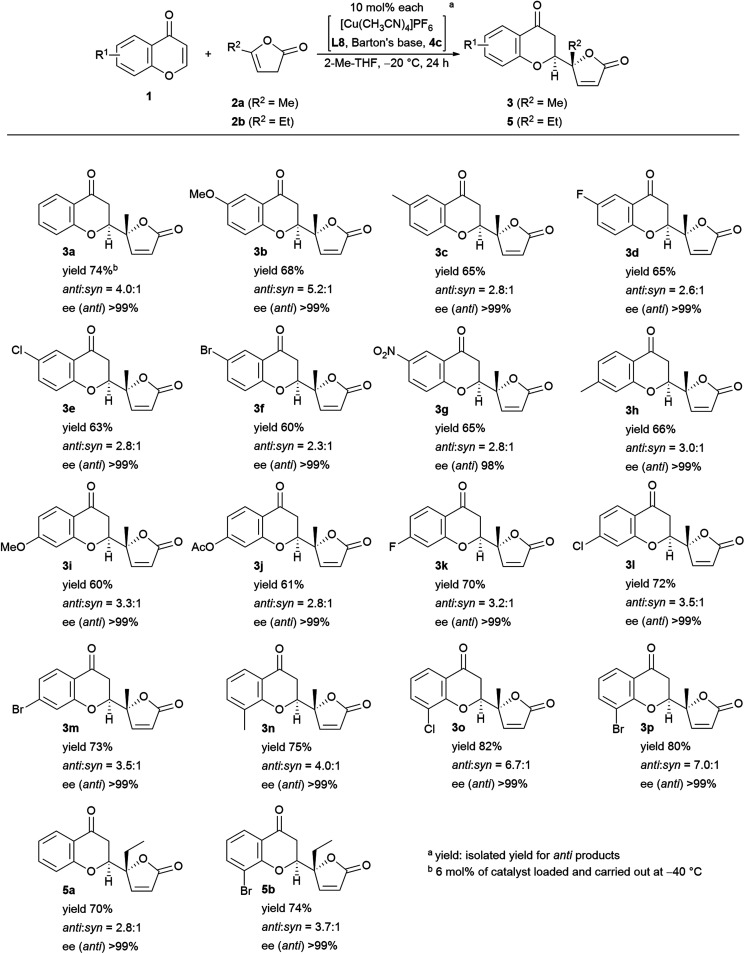
Substrate scope of the vinylogous addition of butenolide to chromone.

Encouraged by the results described above, we investigated the scope of the reaction in terms of diversity in both the substrates as shown in Fig. 5. First, all the reactions performed gave >99% ee with one exception: 98% ee (**3g**). Unsubstituted chromone, **1a**, afforded 74% isolated yield of adduct **3a**. The 6-substituted products (R^1^ = MeO (**3b**), Me (**3c**), F (**3d**), Cl (**3e**), Br (**3f**), NO_2_ (**3g**)) were obtained in fair to good diastereoselectivity (up to 5.2 : 1), and a reasonable isolated yield of *anti*-products, from 60% to 73%. The 7-functionalized chromones gave equally good results with a diastereoselectivity up to 3.5 : 1, and an isolated yield of the desired isomer ranging from 61% to 73% (R^1^ = Me (**3h**), MeO (**3i**), AcO (**3j**), F (**3k**), Cl (**3l**), and Br (**3m**)). Substitution at the 8-position showed preferential effects for diastereoselectivity: 4.0 : 1 for **3n** (R^1^ = Me), 6.7 : 1 for **3o** (R^1^ = Cl) in overall highest isolated yield (82%). The highest diastereoselectivity (7.0 : 1) was achieved with 8-bromo-substitution (**3p**). Introduction of an ethyl group instead of the methyl group of the pronucleophile (**2b**) was also tolerable; with chromone (**1a**), the diastereoselectivity was 2.8 : 1 in 70% isolated yield (**5a**), and with 8-bromochomone, the diastereoselectivity was 3.7 : 1 in 74% yield (**5b**, isolated yield for *anti*-adduct).

**Scheme 1:**
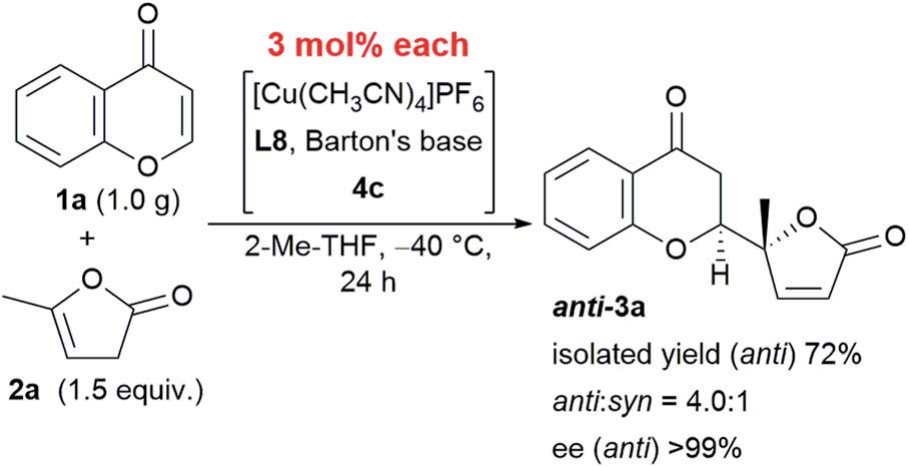
Gram-scale protocol with reduced catalyst loading.

This reaction is characterized not only by its broad substrate scope, but also by its scalability. A gram–scale reaction using 1.0 g of chromone **1a** and 1.5 equivalent of α-angelica lactone **2a** was successfully carried out with even less catalyst (as low as 3 mol%) resulting in a diastereoselectivity of 4.0 : 1 and an enantioselectivity > 99% at −40 °C (Scheme 1). The only difference in the results from that of the smaller scale reaction with 6 mol% catalyst was an isolated yield of 72%, which was within the experimental fluctuation range.

## Conclusions

We report an *anti*-selective catalytic asymmetric vinylogous addition of β,γ-butenolides to chromones. Considering the extremely high intrinsic tendency to produce *syn*-adducts under a substrate-controlled reaction mechanism, the unexpected selectivity reversal was highly remarkable. The catalyst system developed herein is characterized by (1) tuning of the steric and electronic environment within the stereocontrolling transition state using (*R*)-3,4,5-(MeO)_3_-MeOBIPHEP as a chiral ligand to invert diastereoselection, and (2) improvement of the catalyst turnover by a coordinative phenoxide additive to increase the chemical yield. This method will pave the way to the syntheses of natural product-like libraries with high levels of stereoselectivity. Further synthetic studies in this vein will be reported in due course.

## Conflicts of interest

There are no conflicts of interest to declare.

## Supplementary Material

SC-011-D0SC01914C-s001

SC-011-D0SC01914C-s002
