# Supplementary material for: Direct catalytic asymmetric and anti-selective vinylogous addition of butenolides to chromones
Source: Chem Sci. 2020 Jun 22;11(27):7170–6. doi: 10.1039/d0sc01914c (PMC8159409; doi:10.1039/d0sc01914c)
Supplement: SC-011-D0SC01914C-s001 [file SC-011-D0SC01914C-s001.pdf]

Electronic Supplementary Material (ESI) for Chemical Science.
This journal is © The Royal Society of Chemistry 2020

Direct Catalytic Asymmetric and *Anti*-selective Vinylogous Addition of Butenolides to Chromones

Jin Cui, Naoya Kumagai, Takumi Watanabe,* Masakatsu Shibasaki*

Institute of Microbial Chemistry (BIKAKEN), Tokyo, Japan

twatanabe@bikaken.or.jp, mshibasa@bikaken.or.jp

1. General Methods S2

1-1. Reaction and Purification
1-2. Instrumentation
1-3. Materials

2. General Procedures S3

2-1. General Procedure for the Synthesis of Chromanones
2-2. General Procedure for Direct Vinylogous Addition of Butenolides to Chromones

3. Crystal structure analysis S10

4. Appendages S12

5. References S13

6. NMR Spectra of New Compounds (^{1}H and ^{13}C NMR spectra) S14

1. General Methods

1-1. Reaction and Purification

The catalytic asymmetric vinylogous addition was performed in a flame-dried round-bottomed flask with a Teflon-coated magnetic stirring bar. The test tubes or round-bottomed flask were fitted with a 3-way glass stopcock under Ar atmosphere unless otherwise noted. Air- and moisture-sensitive liquids were transferred via a gas-tight syringe and a stainless-steel needle. All work-up and purification procedures were carried out with reagent-grade solvents under ambient atmosphere.

1-2. Instrumentation

NMR was recorded on JEOL ECS-400 spectrometer. Chemical shifts for proton are referenced to residual protium in the NMR solvent ($CDCl_3$: δ 7.26 ppm). For ^{13}C NMR chemical shifts are reported in the scale relative to NMR solvent ($CDCl_3$: δ 77.0 ppm) as an internal reference. NMR data are reported as follows: chemical shifts, multiplicity (s: singlet, d: doublet, dd: doublet of doublets, t: triplet, q: quartet, m: multiplet, br: broad signal), coupling constant (Hz), and integration. Single-crystal X-ray data were collected on a Rigaku R-AXIS RAPID II imaging plate area detector with graphite-monochromated Cu-Kα radiation. Optical rotation was measured using a 2 mL cell with a 1.0 dm path length on a JASCO polarimeter P-1030. High resolution mass spectra (ESI Orbitrap (+)) were measured on ThermoFisher Scientific LTQ Orbitrap XL. Melting points were measured on a Yanagimoto Seisakusho Micro Melting Point Apparatus. HPLC analysis was conducted on a JASCO HPLC system equipped with Daicel chiral-stationary-phase columns (0.46 cm ϕ × 25 cm). Infrared (IR) spectra were recorded on a JASCO FT/IR-4100 Fourier transform infrared spectrophotometer.

1-3. Materials

Unless otherwise noted, materials were purchased from commercial suppliers and were used without purification. Toluene, THF, diethyl ether, and CH_2Cl_2 were purified by passing through a solvent purification system (Glass Contour). Anhydrous 2-methyltetrahydrofuran (2-Me-THF) was purchased from Sigma-Aldrich. Ligands and $[Cu(MeCN)_4]PF_6$ were purchased from Strem Chemical, Inc. or Sigma-Aldrich and used as received (opened and handled in a glove box). 4-Methoxyphenol was purchased from TCI and used after recrystallization from *n*-hexane/EtOAc. Column chromatography was performed with silica gel Merck 60 (230–400 mesh ASTM). Preparative TLC plates (1.05744.0001, PLC Silica gel 60 F_{254}) were purchased from Merck.

2. General Procedures

2-1. General Procedure for the Synthesis of Chromanones

The chromanones **1** were prepared from the corresponding substituted *o*-hydroxyacetophenone according to reported methods.[1),2)] The chromanone **1j** was prepared by acylation of 7-hydroxy-4-chromone (Sigma-Aldrich) with acyl chloride and Et_3N in CH_2Cl_2 as a white solid. ^{1}H NMR (400 MHz, $CDCl_3$): δ 8.22 (d, *J* = 8.8 Hz, 1H), 7.83 (d, *J* = 6.0 Hz, 1H), 7.27 (d, *J* = 2.0 Hz, 1H), 7.15 (dd, *J* = 8.8 Hz, 2.0 Hz, 1H), 6.33 (d, *J* = 6.0 Hz, 1H), 2.35 (s, 3H); ^{13}C NMR (100 MHz, $CDCl_3$): δ 176.77, 168.48, 156.94, 155.38, 154.51, 127.27, 122.69, 119.51, 113.14, 111.09, 21.12.

2-2. General Procedure for Direct Vinylogous Addition of Butenolides to Chromones

To a flame-dried 20 mL test tube equipped with a magnetic stirring bar and a 3-way glass stopcock were charged with (*R*)-3,4,5-MeO-MeOBIPHEP (22.6 mg, 0.024 mmol, 10 mol%), 4-methoxyphenol (**4c**) (3.0 mg, 0.024 mmol, 10 mol%) and $[(CuCH_3CN)_4]PF_6$ (8.9 mg, 0.024 mmol, 10 mol%) in a glove box. To this mixture was added dry 2-Me-THF (0.4 mL) via syringe at room temperature. A flame-dried 20 mL test tube was charged with chromones (**1**, 0.24 mmol, 1.0 eq), butenolide (**2**) (0.36 mmol, 1.5 eq) and dry 2-Me-THF (0.32 mL), and reaction mixture was cooled to –20 °C. To the resulting cooled solution was added the (*R*)-3,4,5-MeO-MeOBIPHEP/$[(CuCH_3CN)_4]PF_6$/**4c** solution dropwise within 1 min. Then Barton's base in 2-Me-THF (0.48 mL, 0.024 mmol, 10 mol%; 0.05 M) was added dropwise to run the reaction. After 24 h of stirring at –20 °C, a solution of acetic acid in THF (0.2 mL; 0.1 M). The mixture was concentrated under reduced pressure. The residual product was purified by chromatography on silica gel or preparative thin layer chromatography. The diastereomeric ratio (d.r.) was determined by NMR analysis. Enantiomeric excess was determined by HPLC analysis.

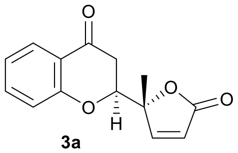

3a

(*S*)-2-((*R*)-2-Methyl-5-oxo-2,5-dihydrofuran-2-yl)chroman-4-one (3a): Prepared by the general procedure with 6 mol% catalyst loading at –40 °C, and isolated by preparative thin layer chromatography (Et_2O/petroleum ether = 4/1) as a white solid (43 mg, 74%, dr = 4:1). Melting point: 115 °C−117 °C; ^{1}H NMR (400 MHz, $CDCl_3$): δ 7.85 (dd, *J* = 8.0 Hz, 1.6 Hz, 1H), 7.62 (d, *J* = 5.6 Hz, 1H), 7.48 (ddd, *J* = 7.6 Hz, 7.6 Hz, 1.6 Hz, 1H), 7.04 (dd, *J* = 7.6 Hz, 7.6 Hz, 1H), 6.98 (d, *J* = 8.4 Hz, 1H), 6.17 (d, *J* = 5.6 Hz, 1H), 4.42 (dd, *J* = 13.2 Hz, 3.2 Hz, 1H), 2.83 (dd, *J* = 16.8 Hz, 13.6 Hz, 1H), 2.73 (dd, *J* = 16.4 Hz, 3.2 Hz, 1H), 1.62 (s, 3H); ^{13}C NMR (100 MHz, $CDCl_3$): δ 190.44, 171.38, 160.35, 157.39, 136.26, 126.97, 122.10, 121.98, 120.73, 117.72, 87.53, 79.55, 37.97, 19.02; IR (KBr): 3085, 2985, 2930, 1762, 1692, 1606, 1578, 1473, 1464, 1380, 1306, 1224, 1149, 1116, 1084, 1056, 1029, 999, 904, 889, 851, 820, 767, 681 cm^{-1}; HRMS (ESI$^+$) [M+Na]$^+$ found 267.0630, $C_{14}H_{12}O_4Na$ requires 267.0628; $[\alpha]_D^{25}$ +148 (*c* 0.21, $CHCl_3$); Enantiomeric excess was determined to be >99% ee by chiral HPLC analysis. HPLC: CHIRALCEL IA (ϕ 0.46 cm x 25 cm), 2-propanol/n-hexane = 5/95, flow rate 0.8 mL/min, detection 254 nm, tR = 51.2 min. A gram scale synthesis using chromone **1a** (1.0 g, 6.75 mmol, 1.0 eq) and **2a** (993 mg, 10.13 mmol, 1.5 eq) with (*R*)-3,4,5-MeO-MeOBIPHEP (192 mg, 0.203 mmol, 3 mol%), 4-methoxyphenol (**4c**) (25 mg, 0.203 mmol, 3 mol%), $[(CuCH_3CN)_4]PF_6$ (76 mg, 0.203 mmol, 3 mol%), and Barton's base in 2-Me-THF (4.06 mL, 0.203 mmol, 3 mol%; 0.05 M) in 2-Me-THF (20 mL) at –40 °C afforded **3a** (72%, dr = 4:1) with pure **3a** (423 mg) and a mixture of diastereomers (1.14 g) containing **3a** (762 mg) by chromatography on silica gel (Et_2O/petroleum ether = 4/1). Enantiomeric excess was determined to be >99% ee by chiral HPLC analysis. HPLC: CHIRALCEL OD-H (ϕ 0.46 cm x 25 cm), 2-propanol/n-hexane = 5/95, flow rate 1.0 mL/min, detection 254 nm, tR = 65.8 min.

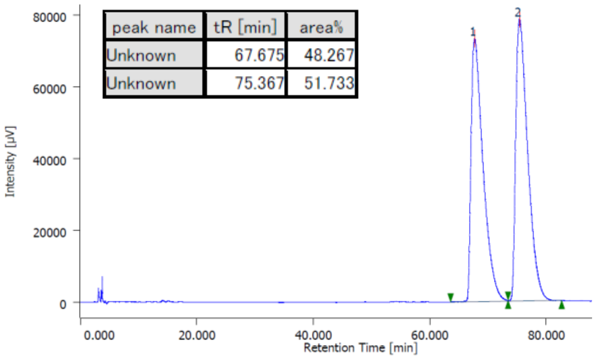

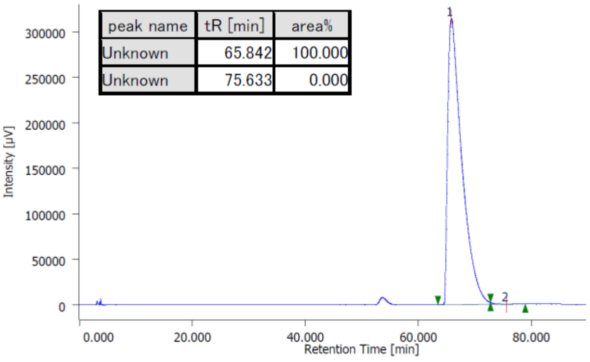

(*S*)-6-Methoxy-2-((*R*)-2-methyl-5-oxo-2,5-dihydrofuran-2-yl)chroman-4-one (3b) Prepared by the general procedure and isolated by preparative thin layer chromatography (Et_2O/petroleum ether = 3/1) as a white solid (44.7 mg, 68%, dr = 5.2:1). Melting point: 122 °C–125 °C; ^{1}H NMR (400 MHz, $CDCl_3$): δ 7.61 (d, *J* = 6.0 Hz, 1H), 7.30 (d, *J* = 3.2 Hz, 1H), 7.10 (dd, *J* = 9.2 Hz, 3.2 Hz, 1H), 6.93 (d, *J* = 8.8 Hz, 1H), 6.18 (d, *J* = 6.0 Hz, 1H), 4.38 (dd, *J* = 13.2 Hz, 3.2 Hz, 1H), 3.80 (s, 3H), 2.79 (m, 2H), 1.62 (s, 3H); ^{13}C NMR (100 MHz, $CDCl_3$): δ 190.56, 171.41, 157.49, 155.07, 154.59, 125.40, 122.00, 120.71, 119.06, 107.41, 87.55, 79.81, 55.80, 38.00, 19.03; IR (KBr): 3100, 3073, 3008, 2964, 2941, 2914, 2835, 1749, 1715, 1683, 1653, 1615, 1586, 1488, 1455, 1432, 1404, 1379, 1361, 1339, 1284, 1245, 1215, 1171, 1148, 1119, 1084, 1055, 1034, 1011, 972, 945, 917, 906, 882, 851, 823, 754, 727, 702, 680, 667, 646, 603, 580, 554 cm^{-1}; HRMS (ESI$^+$) [M+Na]$^+$ found 297.0736, $C_{15}H_{14}O_5Na$ requires 297.0733; $[\alpha]_D^{25}$ +240 (*c* 0.03, $CHCl_3$); Enantiomeric excess was determined to be >99% ee by chiral HPLC analysis. HPLC: CHIRALCEL OD-H (ϕ 0.46 cm x 25 cm), 2-propanol/n-hexane = 5/95, flow rate 1.5 mL/min, detection 254 nm, tR = 62.6 min.

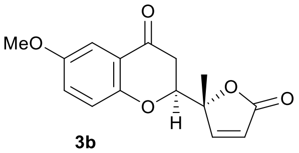

3b

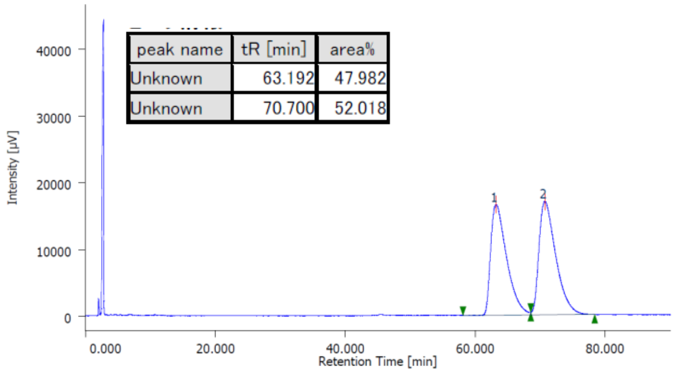

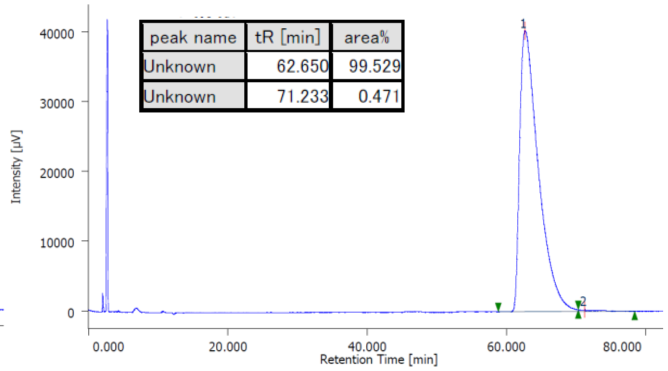

(*S*)-6-Methyl-2-((*R*)-2-methyl-5-oxo-2,5-dihydrofuran-2-yl)chroman-4-one (3c) Prepared by the general procedure and isolated by preparative thin layer chromatography (Et_2O/petroleum ether = 3/1) as a white solid (40.2 mg, 65%, d.r. = 74/26). Melting point: 167 °C–170 °C; ^{1}H NMR (400 MHz, $CDCl_3$): δ 7.67 (m, 1H), 7.61 (d, *J* = 5.6 Hz, 1H), 7.31 (dd, *J* = 8.8 Hz, 2.4 Hz, 1H), 6.90 (d, *J* = 8.4 Hz, 1H), 6.18 (d, *J* = 5.6 Hz, 1H), 4.39 (dd, *J* = 13.2 Hz, 3.2 Hz, 1H), 2.83 (dd, *J* = 16.4 Hz, 13.2 Hz, 1H), 2.73 (dd, *J* = 16.4 Hz, 3.2 Hz, 1H), 2.31 (s, 3H), 1.63 (s, 3H); ^{13}C NMR (100 MHz, $CDCl_3$): δ 190.71, 171.43, 158.50, 157.47, 137.35, 131.73, 126.65, 122.03, 120.43, 117.54, 87.59, 79.67, 38.12, 20.41, 19.05; IR (KBr): 3095, 2980, 2915, 1768, 1750, 1682, 1617, 1490, 1420, 1294, 1248, 1228, 1180, 1111, 1086, 1013, 944, 917, 847, 819, 606, 551, 535 cm^{-1}; HRMS (ESI$^+$) [M+Na]$^+$ found 281.0786, $C_{15}H_{14}O_4Na$ requires 281.0784; $[\alpha]_D^{25}$ +200 (*c* 0.2, $CHCl_3$); Enantiomeric excess was determined to be >99% ee by chiral HPLC analysis. HPLC: CHIRALCEL OD-H (ϕ 0.46 cm x 25 cm), 2-propanol/n-hexane = 5/95, flow rate 1.0 mL/min, detection 254 nm, tR = 57.2 min.

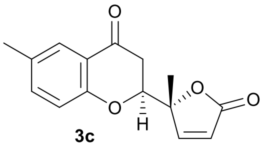

3c

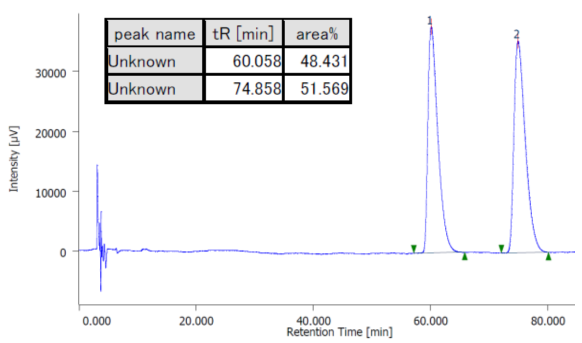

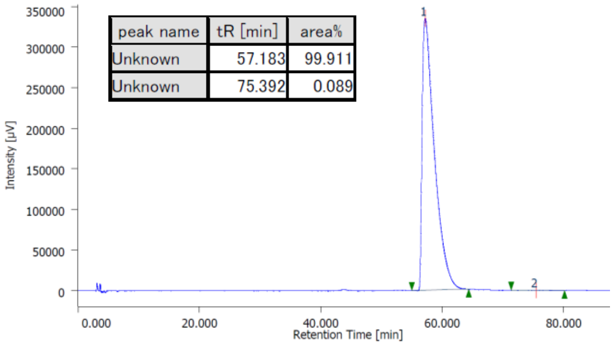

(*S*)-6-Fluoro-2-((*R*)-2-methyl-5-oxo-2,5-dihydrofuran-2-yl)chroman-4-one (3d) Prepared by the general procedure and isolated by preparative thin layer chromatography (Et_2O/petroleum ether = 3/1) as a white solid (40.8 mg, 65%, dr = 2.6:1). Melting point: 160 °C–165 °C; ^{1}H NMR (400 MHz, $CDCl_3$): δ 7.60 (d, *J* = 5.6 Hz, 1H), 7.53 (dd, *J* = 8.0 Hz, 3.2 Hz, 1H), 7.23 (m, 1H), 6.99 (dd, *J* = 8.8 Hz, 4.0 Hz, 1H), 6.20 (d, *J* = 5.6 Hz, 1H), 4.42 (dd, *J* = 12.8 Hz, 4.0 Hz, 1H), 2.81 (m, 2H), 1.63 (s, 3H); ^{13}C NMR (100 MHz, $CDCl_3$): δ 189.66, 189.65, 171.26, 158.81, 157.13, 156.61, 156.59, 156.39, 123.94, 123.70, 122.22, 121.36, 121.29, 119.54, 119.46, 112.28, 112.04, 87.40, 79.89, 37.81, 19.11; IR (KBr): 3087, 2986, 2922, 2872, 2852, 1760, 1697, 1619, 1483, 1437, 1405, 1381, 1337, 1274, 1243, 1190, 1169, 1150, 1117, 1082, 1049, 1010, 948, 91, 905, 890, 879, 850, 822, 808, 756, 727, 708, 540 cm^{-1}; HRMS (ESI$^+$) [M+Na]$^+$ found 285.0536, $C_{14}H_{11}O_4FNa$ requires 285.0534; $[\alpha]_D^{25}$ +152 (*c* 0.045, $CHCl_3$); Enantiomeric excess was determined to be >99% ee by chiral HPLC analysis. HPLC: CHIRALCEL OD-H (ϕ 0.46

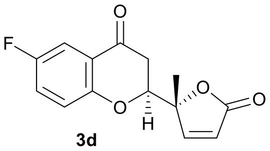

3d

cm x 25 cm), 2-propanol/n-hexane = 5/95, flow rate 1.5 mL/min, detection 254 nm, tR = 60.8 min.

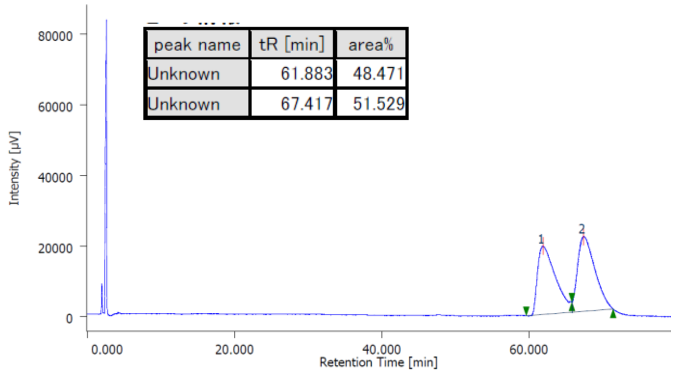

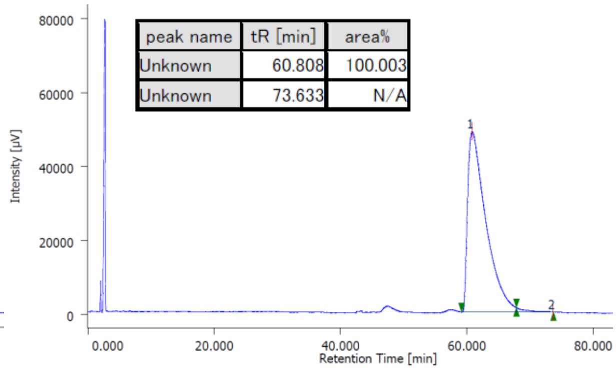

(*S*)-6-Chloro-2-((*R*)-2-methyl-5-oxo-2,5-dihydrofuran-2-yl)chroman-4-one (3e) Prepared by the general procedure and isolated by preparative thin layer chromatography (Et_2O/petroleum ether = 4/1) as a white solid (42 mg, 63%, dr = 2.8:1). Melting point: 158 °C–164 °C; ^1H NMR (400 MHz, $CDCl_3$): δ 7.84 (d, *J* = 2.8 Hz, 1H), 7.59 (d, *J* = 5.6 Hz, 1H), 7.45 (dd, *J* = 8.8 Hz, 2.4 Hz, 1H), 6.97 (d, *J* = 9.2 Hz, 1H), 6.20 (d, *J* = 5.6 Hz, 1H), 4.43 (dd, *J* = 12.4 Hz, 4.0 Hz, 1H), 2.81 (m, 2H), 1.63 (s, 3H); ^{13}C NMR (100 MHz, $CDCl_3$): δ 189.29, 171.20, 158.80, 157.01, 136.15, 127.85, 126.45, 122.30, 121.59, 119.51, 87.37, 79.84, 37.77, 19.15; IR (KBr): 3081, 2916, 1767, 1755, 1695, 1601, 1573, 1470, 1423, 1380, 1361, 1336, 1276, 1246, 1227, 1216, 1197, 1151, 1128, 1111, 1095, 1084, 1056, 1006, 974, 945, 917, 889, 862, 844, 826, 789, 755, 727, 680, 603 cm^{-1}; HRMS (ESI$^+$) [M+Na]$^+$ found 301.0241, $C_{14}H_{11}O_4ClNa$ requires 301.0238; $[\alpha]_D^{25}$ +195 (*c* 0.05, $CHCl_3$); Enantiomeric excess was determined to be >99% ee by chiral HPLC analysis. HPLC: CHIRALCEL OD-H (ϕ 0.46 cm x 25 cm), 2-propanol/n-hexane = 1/9, flow rate 1.5 mL/min, detection 254 nm, tR = 35.9 min.

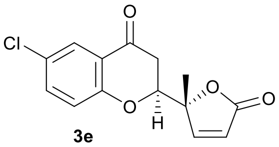

3e

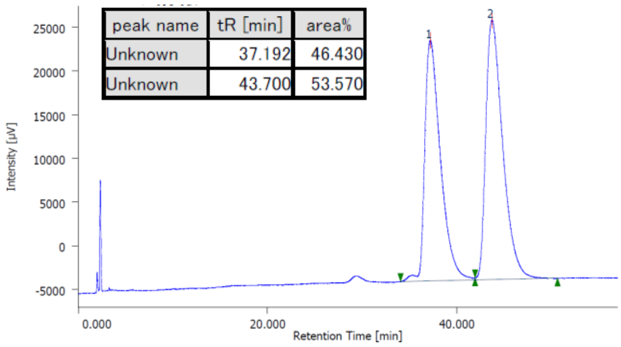

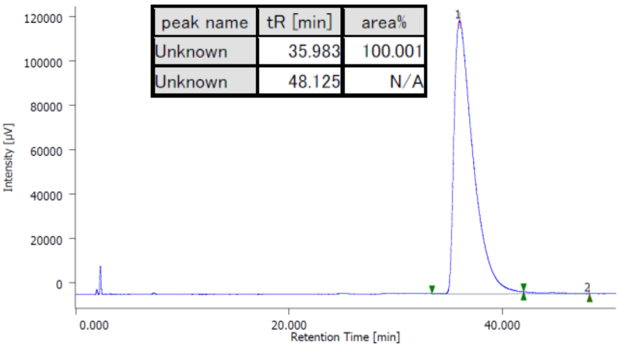

(*S*)-6-Bromo-2-((*R*)-2-methyl-5-oxo-2,5-dihydrofuran-2-yl)chroman-4-one (3f) Prepared by the general procedure and isolated by preparative thin layer chromatography (Et_2O/petroleum ether = 3/1) as a white solid (46 mg, 60%, dr = 2.3:1). Melting point: 170 °C–175 °C; ^1H NMR (400 MHz, $CDCl_3$): δ 7.99 (d, *J* = 2.4 Hz, 1H), 7.58 (dd, *J* = 8.8 Hz, 2.8 Hz, 1H), 7.58 (d, *J* = 6.0 Hz, 1H), 6.92 (d, *J* = 8.8 Hz, 1H), 6.20 (d, *J* = 5.6 Hz, 1H), 4.42 (dd, *J* = 12.4 Hz, 4.0 Hz, 1H), 2.81 (m, 2H), 1.63 (s, 3H); ^{13}C NMR (100 MHz, $CDCl_3$): δ 189.15, 171.19, 159.26, 156.99, 138.95, 129.57, 122.32, 122.05, 119.84, 114.98, 87.36, 79.81, 37.72, 19.15; IR (KBr): 3077, 3055, 2968, 2938, 2915, 1764, 1752, 1691, 1600, 1569, 1469, 1419, 1379, 1358, 1335, 1276, 1227, 1196, 1156, 1130, 1111, 1087, 1006, 973, 944, 919, 855, 841, 826, 781, 757, 726, 678, 648, 600, 550, 530 cm^{-1}; HRMS (ESI$^+$) [M+Na]$^+$ found 344.9734, $C_{14}H_{11}O_4BrNa$ requires 344.9733; $[\alpha]_D^{25}$ +182 (*c* 0.055, $CHCl_3$); Enantiomeric excess was determined to be 99.2% ee by chiral HPLC analysis. HPLC: CHIRALCEL OD-H (ϕ 0.46 cm x 25 cm), 2-propanol/n-hexane = 1/9, flow rate 1.5 mL/min, detection 254 nm, tR = 39.7 min.

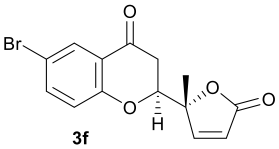

3f

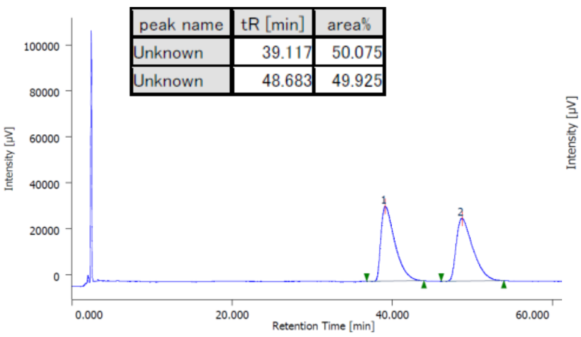

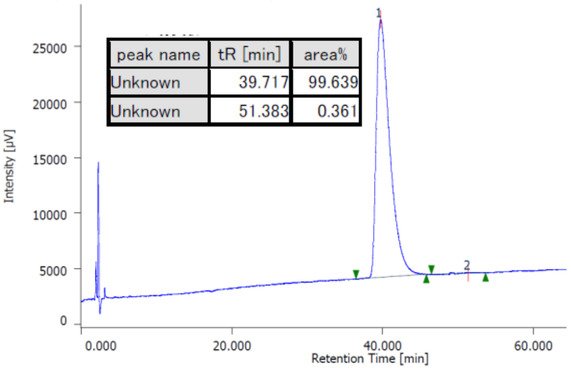

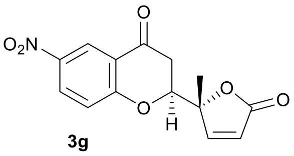

3g

(*S*)-2-((*R*)-2-Methyl-5-oxo-2,5-dihydrofuran-2-yl)-6-nitrochroman-4-one (3g) Prepared by the general procedure and isolated by preparative thin layer chromatography (Et_2O/petroleum ether = 3/1) as a white solid (45 mg, 65%, dr = 2.8:1). Melting point: 170 °C–173 °C; ^1H NMR (400 MHz, $CDCl_3$): δ 8.77 (d, *J* = 2.4 Hz, 1H), 8.37 (dd, *J* = 9.2 Hz, 2.8 Hz, 1H), 7.58 (d, *J* = 5.6 Hz, 1H), 7.17 (d, *J* = 9.2 Hz, 1H), 6.24 (d, *J* = 5.6 Hz, 1H), 4.58 (dd, *J* = 10.0 Hz, 6.8 Hz, 1H), 2.88 (m, 2H), 1.68 (s, 3H); ^{13}C NMR (100 MHz, $CDCl_3$): δ 188.19, 170.88, 164.03, 156.36, 142.70, 130.63, 123.56, 122.74, 120.43, 119.22, 87.17, 80.27, 37.49, 19.38; IR (KBr): 3094, 2920, 1763, 1703, 1653, 1616, 1586, 1523, 1473, 1438, 1405, 1381, 1346, 1278, 1244, 1220, 1147, 1129, 1108, 1080, 1057, 1004, 950, 907, 880, 820, 750 cm^{-1}; HRMS (ESI$^+$) $[M+Na]^+$ found 312.0482, $C_{14}H_{11}O_6NNa$ requires 312.0479; $[\alpha]_D^{25}$ +187 (*c* 0.045, $CHCl_3$); Enantiomeric excess was determined to be 98.3% ee by chiral HPLC analysis. HPLC: CHIRALCEL OD-H (ϕ 0.46 cm x 25 cm), 2-propanol/n-hexane = 15/85, flow rate 1.0 mL/min, detection 254 nm, tR = 116.4 min.

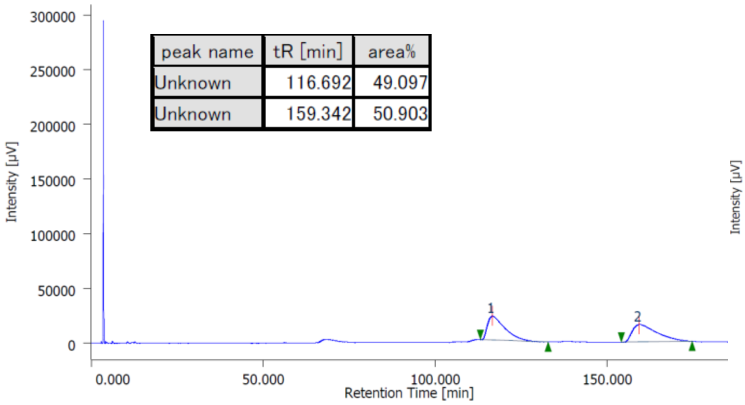

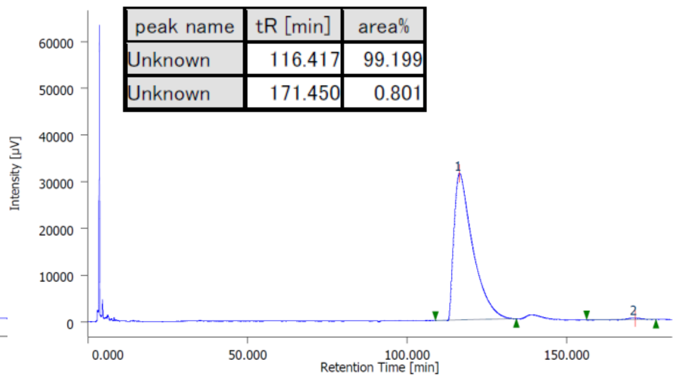

(*S*)-7-Methyl-2-((*R*)-2-methyl-5-oxo-2,5-dihydrofuran-2-yl)chroman-4-one (3h) Prepared by the general procedure and isolated by preparative thin layer chromatography (Et_2O/petroleum ether = 3/1) as a white solid (40.8 mg, 66%, dr = 3:1). Melting point: 140 °C–142 °C; ^1H NMR (400 MHz, $CDCl_3$): δ 7.76 (d, *J* = 8.0 Hz, 1H), 7.60 (d, *J* = 5.6 Hz, 1H), 6.87 (d, *J* = 8.0 Hz, 1H), 6.81 (brs, 1H), 6.18 (d, *J* = 5.6 Hz, 1H), 4.41 (dd, *J* = 13.2 Hz, 3.2 Hz, 1H), 2.76 (m, 2H), 2.36 (s, 3H), 1.62 (s, 3H); ^{13}C NMR (100 MHz, $CDCl_3$): δ 190.13, 171.41, 160.44, 157.34, 148.00, 126.94, 123.51, 122.07, 118.57, 117.79, 87.59, 79.65, 38.01, 21.91, 19.09; IR (KBr): 3097, 2981, 2955, 2923, 1796, 1767, 1680, 1615, 1571, 1507, 1493, 1455, 1423, 1384, 1368, 1345, 1329, 1302, 1289, 1254, 1240, 1220, 1207, 1185, 1159, 1130, 1115, 1092, 1054, 1041, 1019, 1005, 983, 953, 943, 900, 889, 872, 845, 816, 781, 757, 690, 590, 552, 518 cm^{-1}; HRMS (ESI$^+$) $[M+Na]^+$ found 281.0786, $C_{15}H_{14}O_4Na$ requires 281.0784; $[\alpha]_D^{25}$ +200 (*c* 0.025, $CHCl_3$); $[\alpha]_D^{25}$ +277 (*c* 0.2, $CHCl_3$); Enantiomeric excess was determined to be >99% ee by chiral HPLC analysis. HPLC: CHIRALCEL OD-H (ϕ 0.46 cm x 25 cm), 2-propanol/n-hexane = 5/95, flow rate 0.8 mL/min, detection 254 nm, tR = 78.1 min.

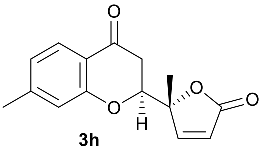

3h

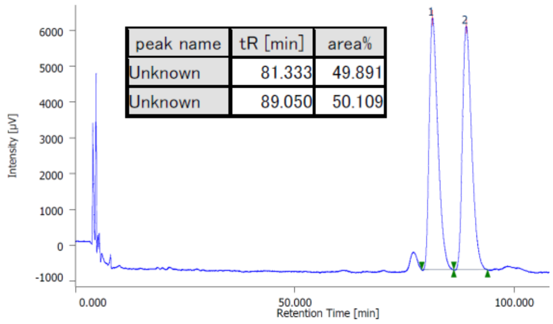

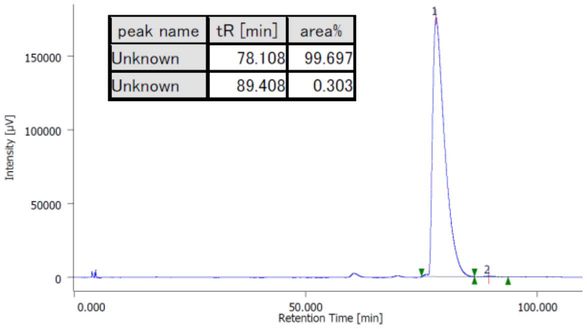

(*S*)-7-Methoxy-2-((*R*)-2-methyl-5-oxo-2,5-dihydrofuran-2-yl)chroman-4-one (3i) Prepared by the general procedure and isolated by preparative thin layer chromatography (Et_2O/petroleum ether = 3/1) as a white solid (39.5 mg, 60%, dr = 3.3:1). Melting point: 138 °C–140 °C; ^1H NMR (400 MHz, $CDCl_3$): δ 7.81 (d, *J* = 8.8 Hz, 1H), 7.58 (d, *J* = 5.6 Hz, 1H), 6.61 (dd, *J* = 8.8 Hz, 2.0 Hz, 1H), 6.43 (d, *J* = 2.0 Hz, 1H), 6.19 (d, *J* = 5.6 Hz, 1H), 4.45 (dd, *J* = 13.2 Hz, 3.6 Hz, 1H), 3.84 (s, 3H), 2.73 (m, 2H), 1.62 (s, 3H); ^{13}C NMR (100 MHz, $CDCl_3$): δ 188.99, 171.43, 166.27, 162.40, 157.05, 128.75, 122.21, 114.60, 110.69, 100.76, 87.66, 79.87, 55.73, 37.75, 19.28; IR (KBr): 2918, 2849, 1767, 1716, 1682, 1652, 1608, 1575, 1558, 1541, 1519, 1507, 1497, 1489, 1455, 1445, 1396, 1375, 1361, 1338, 1261, 1198, 1161, 1116, 1061, 1024, 1006, 953, 903, 819 cm^{-1}; HRMS (ESI$^+$) $[M+Na]^+$ found 297.0737, $C_{15}H_{14}O_5Na$ requires 297.0733; $[\alpha]_D^{25}$ +204 (*c* 0.045, $CHCl_3$); Enantiomeric excess was determined to be 99.3% ee by chiral HPLC analysis. HPLC: CHIRALCEL IA (ϕ 0.46 cm x 25 cm), 2-propanol/n-hexane = 5/95, flow rate 1.0 mL/min, detection 254 nm, tR = 76.8 min.

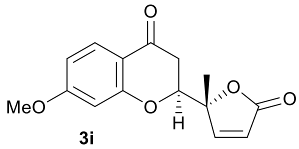

3i

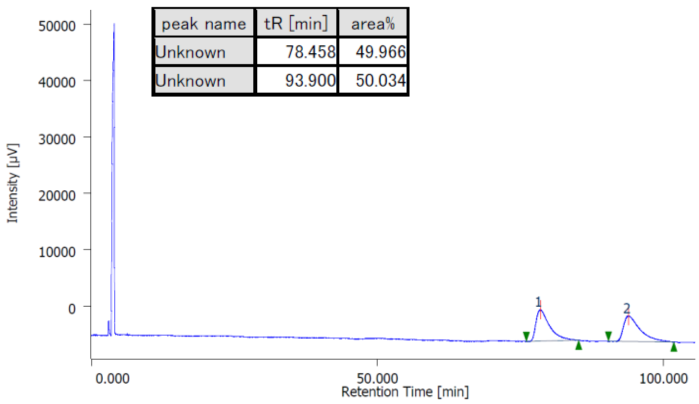

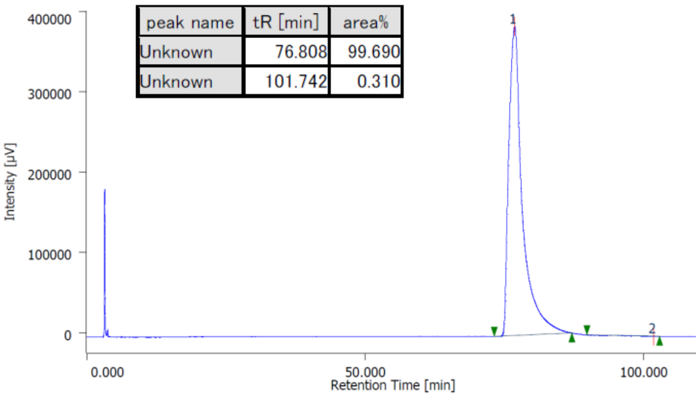

(*S*)-2-((*R*)-2-Methyl-5-oxo-2,5-dihydrofuran-2-yl)-4-oxochroman-7-yl acetate (3j) Prepared by the general procedure and isolated by preparative thin layer chromatography (Et_2O/petroleum ether = 3/1) as a white solid (44 mg, 61%, dr = 2.8:1). Melting point: 136 °C−139 °C; ^1H NMR (400 MHz, $CDCl_3$): δ 7.90 (d, *J* = 8.8 Hz, 1H), 7.59 (d, *J* = 5.6 Hz, 1H), 6.80 (m, 2H), 6.19 (d, *J* = 5.6 Hz, 1H), 4.45 (dd, *J* = 13.2 Hz, 3.2 Hz, 1H), 2.79 (m, 2H), 2.31 (s, 3H), 1.62 (s, 3H); ^{13}C NMR (100 MHz, $CDCl_3$): δ 189.31, 171.28, 168.48, 161.20, 157.20, 156.61, 128.55, 122.16, 118.64, 116.17, 110.94, 87.38, 80.02, 37.80, 21.11, 19.04; IR (KBr): 3077, 2917, 2849, 1767, 1715, 1694, 1651, 1612, 1582, 1557, 1540, 1518, 1507, 1486, 1472, 1441, 1369, 1337, 1287, 1245, 1196, 1141, 1118, 1050, 1013, 949, 903, 844, 818, 784 cm^{-1}; HRMS (ESI$^+$) $[M+Na]^+$ found 325.0684, $C_{16}H_{14}O_6Na$ requires 325.0683; $[\alpha]_D^{25}$ +168 (*c* 0.04, $CHCl_3$); Enantiomeric excess was determined to be >99% ee by chiral HPLC analysis. HPLC: CHIRALCEL IB (ϕ 0.46 cm x 25 cm), 2-propanol/n-hexane = 4/96, flow rate 1.0 mL/min, detection 254 nm, tR = 169.7 min.

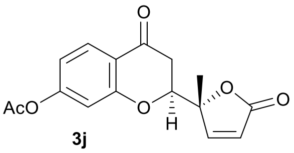

3j

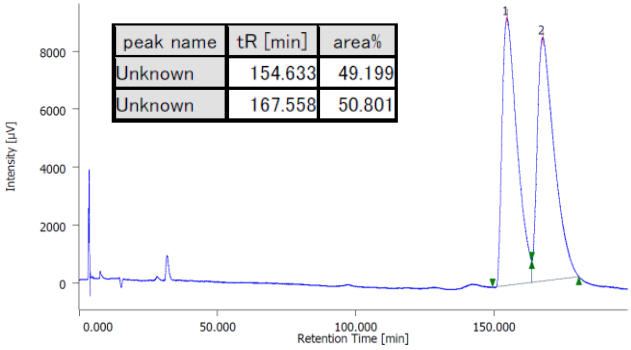

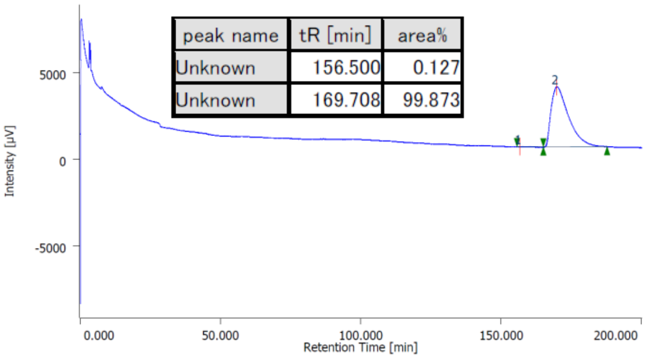

(*S*)-7-Fluoro-2-((*R*)-2-methyl-5-oxo-2,5-dihydrofuran-2-yl)chroman-4-one (3k) Prepared by the general procedure and isolated by preparative thin layer chromatography (Et_2O/petroleum ether = 3/1) as a white solid (44 mg, 70%, dr = 3.2:1). Melting point: 120 °C−125 °C; ^1H NMR (400 MHz, $CDCl_3$): δ 7.90 (dd, *J* = 8.8 Hz, 5.8 Hz, 1H), 7.59 (d, *J* = 6.0 Hz, 1H), 6.78 (ddd, *J* = 8.8 Hz, 8.8 Hz, 2.4 Hz, 1H), 6.70 (dd, *J* = 9.6 Hz, 2.0 Hz, 1H), 4.46 (dd, *J* = 12.8 Hz, 3.2 Hz, 1H), 2.79 (m, 2H), 1.63 (s, 3H); 13C NMR (100 MHz, CDCl3): δ 188.93, 171.21, 168.79, 166.24, 162.02, 161.89, 157.02, 129.70, 129.58, 122.26, 117.72, 117.70, 110.74, 110.52, 104.87, 104.63, 87.35, 80.16, 37.69, 19.11; IR (KBr): 3114, 3088, 2988, 2916, 2849, 2359, 1828, 1769, 1749, 1715, 1696, 1651, 1615, 1589, 1557, 1540, 1519, 1507, 1488, 1473, 1445, 1385, 1337, 1286, 1254, 1190, 1142, 1105, 1056, 1007, 958, 915, 848, 829, 808, 785, 721, 681, 669, 636 617, 557, 532 cm^{-1}; HRMS (ESI$^+$) $[M+Na]^+$ found 285.0537, $C_{14}H_{11}O_4FNa$ requires 285.0534; $[\alpha]_D^{25}$ +161 (*c* 0.055, $CHCl_3$); Enantiomeric excess was determined to be >99% ee by chiral HPLC analysis. HPLC: CHIRALCEL OD-H (ϕ 0.46 cm x 25 cm), 2-propanol/n-hexane = 5/95, flow rate 0.8 mL/min, detection 254 nm, tR = 92.8 min.

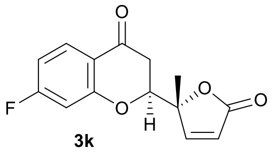

3k

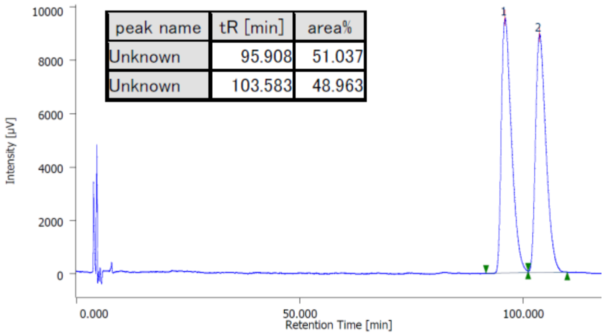

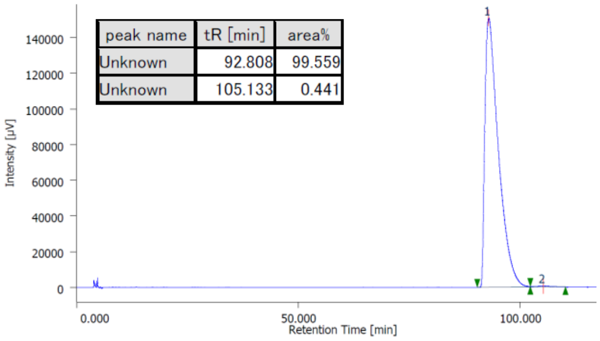

(*S*)-7-Chloro-2-((*R*)-2-methyl-5-oxo-2,5-dihydrofuran-2-yl)chroman-4-one (3l) Prepared by the general procedure and

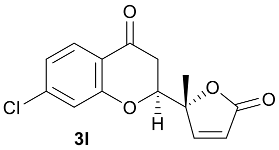

3l

isolated by preparative thin layer chromatography (Et_2O/petroleum ether = 3/1) as a white solid (48 mg, 72%, dr = 3.5:1). Melting point: 124 °C−126 °C; ^{1}H NMR (400 MHz, $CDCl_3$): δ 8.02 (dd, *J* = 8.8 Hz, 5.8 Hz, 1H), 7.80 (d, *J* = 5.6 Hz, 1H), 7.25 (m, 2H), 6.41 (d, *J* = 5.6 Hz, 1H), 3.01 (m, 2H), 1.84 (s, 3H); 13C NMR (100 MHz, CDCl3): δ 189.29, 171.18, 160.62, 157.04, 142.21, 128.27, 123.00, 122.25, 119.33, 118.00, 87.32, 80.01, 37.79, 19.08; IR (KBr): 3118, 3093, 2981, 2916, 1759, 1697, 1653, 1600, 1567, 1472, 1455, 1428, 1380, 1362, 1339, 1315, 1286, 1274, 1244, 1215, 1186, 1149, 1122, 1082, 1055, 1005, 955, 918, 905, 856, 844, 820, 774, 756, 734 cm^{-1}; HRMS (ESI$^+$) $[M+Na]^+$ found 301.0242, $C_{14}H_{11}O_4ClNa$ requires 301.0238; $[\alpha]_D^{25}$ +200 (*c* 0.06, $CHCl_3$); Enantiomeric excess was determined to be >99% ee by chiral HPLC analysis. HPLC: CHIRALCEL OD-H (*ϕ* 0.46 cm x 25 cm), 2-propanol/n-hexane = 5/95, flow rate 1.0 mL/min, detection 254 nm, tR = 85.6 min.

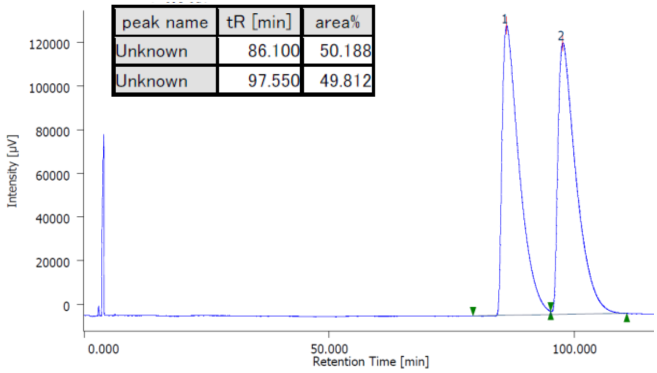

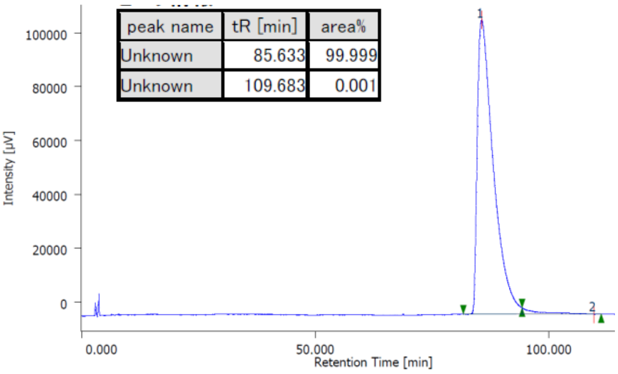

(*S*)-7-Bromo-2-((*R*)-2-methyl-5-oxo-2,5-dihydrofuran-2-yl)chroman-4-one (3m) Prepared by the general procedure and

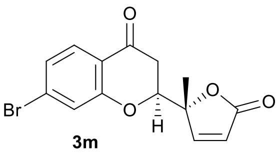

3m

isolated by preparative thin layer chromatography (Et_2O/petroleum ether = 3/1) as a white solid (56 mg, 73%, dr = 3.5:1). Melting point: 128 °C−133 °C; ^{1}H NMR (400 MHz, $CDCl_3$): δ 7.76 (d, *J* = 8.4 Hz, 1H), 7.62 (d, *J* = 5.6 Hz, 1H), 7.25 (m, 2H), 6.23 (d, *J* = 5.6 Hz, 1H), 2.82 (m, 2H), 1.66 (s, 3H); 13C NMR (100 MHz, CDCl3): δ 189.45, 171.17, 160.46, 157.02, 130.70, 128.24, 125.84, 122.24, 121.03, 119.66, 87.31, 79.98, 37.81, 19.06; IR (KBr): 2917, 2849, 2358, 1791, 1769, 1757, 1715, 1696, 1651, 1635, 1592, 1557, 1540, 1518, 1507, 1488, 1471, 1455, 1419, 1396, 1375, 1361, 1338, 1313, 1282, 1242, 1211, 1118, 1070, 1002, 952, 902, 853, 815 cm^{-1}; HRMS (ESI$^+$) $[M+Na]^+$ found 344.9738, $C_{14}H_{11}O_4BrNa$ requires 344.9733; $[\alpha]_D^{25}$ +230 (*c* 0.05, $CHCl_3$); Enantiomeric excess was determined to be 99.5% ee by chiral HPLC analysis. HPLC: CHIRALCEL OD-H (*ϕ* 0.46 cm x 25 cm), 2-propanol/n-hexane = 5/95, flow rate 1.0 mL/min, detection 254 nm, tR = 89.5 min.

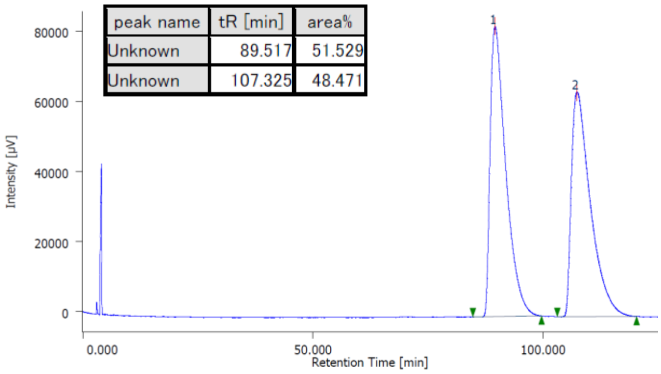

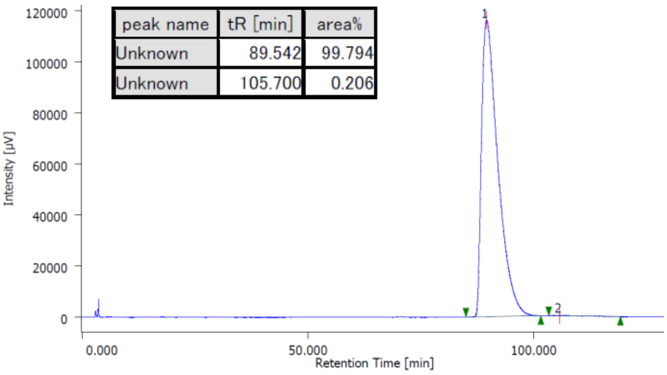

(*S*)-8-Methyl-2-((*R*)-2-methyl-5-oxo-2,5-dihydrofuran-2-yl)chroman-4-one (3n) Prepared by the general procedure and

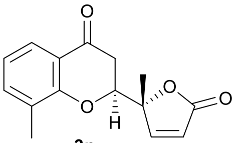

3n

isolated by chromatography on silica gel (Et_2O/petroleum ether = 3/1) as a white solid (46 mg, 75%, dr = 4/1). Melting point: 168 °C−170 °C; ^{1}H NMR (400 MHz, $CDCl_3$): δ 7.72 (d, *J* = 7.6 Hz, 1H), 7.58 (d, *J* = 6.0 Hz, 1H), 7.36 (d, *J* = 7.2 Hz, 1H), 6.95 (dd, *J* = 7.6 Hz, 7.6 Hz, 1H), 6.20 (d, *J* = 5.6 Hz, 1H), 4.45 (dd, *J* = 13.2 Hz, 3.2 Hz, 1H), 2.78 (m, 2H), 2.25 (s, 3H), 1.66 (s, 3H); ^{13}C NMR (100 MHz, $CDCl_3$): δ 190.79, 171.38, 158.57, 157.01, 137.20, 127.03, 124.66, 122.25, 121.57, 120.44, 87.78, 79.41, 37.96, 19.28, 15.46; IR (KBr): 3073, 2917, 1750, 1715, 1682, 1599, 1479, 1456, 1435, 1338, 1303, 1271, 1260, 1243, 1223, 1203, 1156, 1106, 1090, 1010, 943, 915, 840, 826, 786, 741 cm^{-1}; HRMS (ESI$^+$) $[M+Na]^+$ found 281.0785, $C_{15}H_{14}O_4Na$ requires 281.0784; $[\alpha]_D^{25}$ +203 (*c* 0.055, $CHCl_3$); Enantiomeric excess was determined to be >99% ee by chiral HPLC analysis. HPLC: CHIRALCEL IA (*ϕ* 0.46 cm x 25 cm), 2-propanol/n-hexane = 5/95, flow rate 0.6 mL/min, detection 254 nm, tR = 58.3 min.

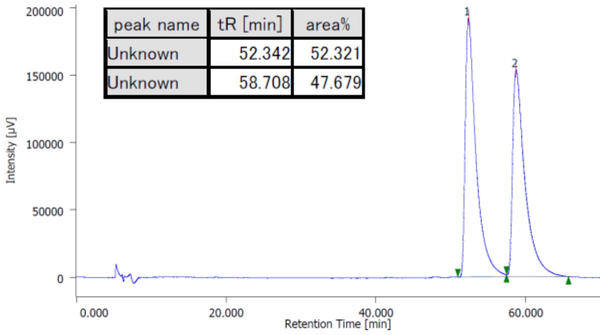

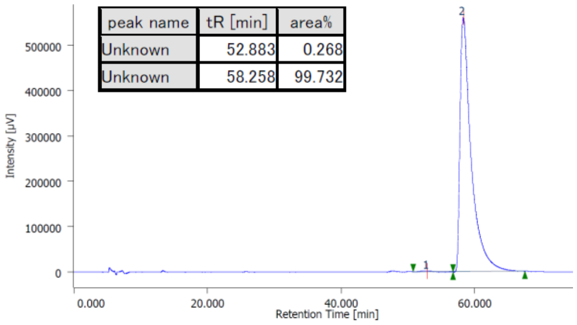

(*S*)-8-Chloro-2-((*R*)-2-methyl-5-oxo-2,5-dihydrofuran-2-yl)chroman-4-one (3o) Prepared by the general procedure and isolated by chromatography on silica gel (Et_2O/petroleum ether = 4/1) as a white solid (54.7 mg, 82%, dr = 6.7:1). Melting point: 182 °C−186 °C; ^{1}H NMR (400 MHz, $CDCl_3$): δ 7.72 (dd, *J* = 7.6 Hz, 1.2 Hz, 1H), 7.69 (d, *J* = 5.6 Hz, 1H), 7.58 (dd, *J* = 8.0 Hz, 1.6 Hz, 1H), 7.01 (dd, *J* = 8.0 Hz, 8.0 Hz, 1H), 6.21 (d, *J* = 5.6 Hz, 1H), 4.45 (dd, *J* = 12.8 Hz, 3.2 Hz, 1H), 2.87 (m, 2H), 1.68 (s, 3H); ^{13}C NMR (100 MHz, $CDCl_3$): δ 189.63, 171.24, 157.66, 155.90, 136.28, 125.58, 122.87, 122.24, 122.09, 122.05, 87.29, 80.34, 37.66, 18.78; IR (KBr): 3092, 2916, 2848, 1750, 1715, 1685, 1651, 1596, 1557, 1539, 1507, 1468, 1455, 1440, 1416, 1362, 1294, 1269, 1239, 1198, 1139, 1110, 1081, 1066, 1005, 943, 913, 862, 817, 732 cm^{-1}; HRMS (ESI$^+$) $[M+Na]^+$ found 301.0240, $C_{14}H_{11}O_4ClNa$ requires 301.0238; $[\alpha]_D^{25}$ +190 (*c* 0.045, $CHCl_3$); Enantiomeric excess was determined to be >99% ee by chiral HPLC analysis. HPLC: CHIRALCEL OD-H (*ϕ* 0.46 cm x 25 cm), 2-propanol/n-hexane = 5/95, flow rate 0.8 mL/min, detection 254 nm, tR = 93.1 min.

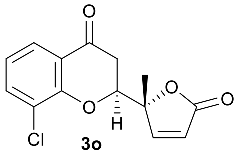

3o

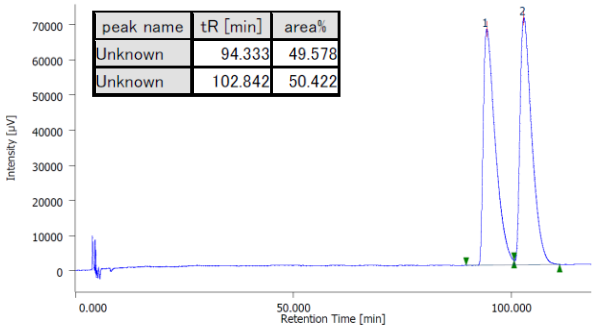

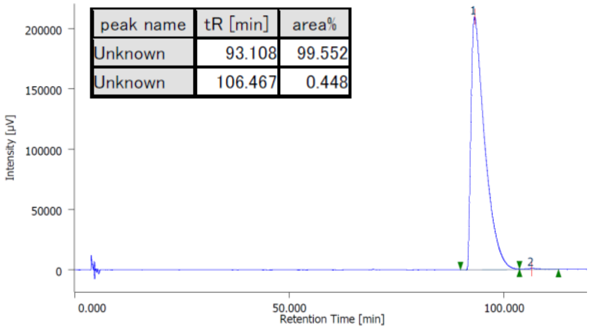

(*S*)-8-Bromo-2-((*R*)-2-methyl-5-oxo-2,5-dihydrofuran-2-yl)chroman-4-one (3p) Prepared by the general procedure and isolated by chromatography on silica gel (Et_2O/petroleum ether = 4/1) as a white solid (61.8 mg, 80%, dr = 7.0:1). Melting point: 177 °C−180 °C; ^{1}H NMR (400 MHz, $CDCl_3$): δ 7.83 (dd, *J* = 7.6 Hz, 1.2 Hz, 1H), 7.74 (dd, *J* = 7.6 Hz, 1.6 Hz, 1H), 7.69 (d, *J* = 5.6 Hz, 1H), 7.01 (dd, *J* = 7.6 Hz, 7.6 Hz, 1H), 6.21 (d, *J* = 5.6 Hz, 1H), 4.45 (dd, *J* = 13.2 Hz, 3.6 Hz, 1H), 2.85 (m, 2H), 1.68 (s, 3H); ^{13}C NMR (100 MHz, $CDCl_3$): δ 189.64, 171.25, 157.65, 156.74, 139.37, 126.36, 122.87, 122.07, 122.01, 111.68, 87.32, 80.33, 37.58, 18.84; IR (KBr): 3092, 2992, 2916, 2848, 1749, 1715, 1684, 1651, 1593, 1557, 1541, 1507, 1488, 1462, 1436, 1417, 1376, 1362, 1335, 1291, 1268, 1238, 1196, 1181, 1153, 1133, 1110, 1087, 1061, 1004, 967, 942, 914, 856, 838, 823, 779, 758, 731, 722, 683, 669 cm^{-1}; HRMS (ESI$^+$) $[M+Na]^+$ found 344.9736, $C_{14}H_{11}O_4BrNa$ requires 344.9733; $[\alpha]_D^{25}$ +177 (*c* 0.08, $CHCl_3$); Enantiomeric excess was determined to be >99% ee by chiral HPLC analysis. HPLC: CHIRALCEL OD-H (*ϕ* 0.46 cm x 25 cm), 2-propanol/n-hexane = 5/95, flow rate 1.0 mL/min, detection 254 nm, tR = 81.5 min.

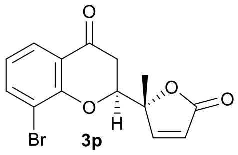

3p

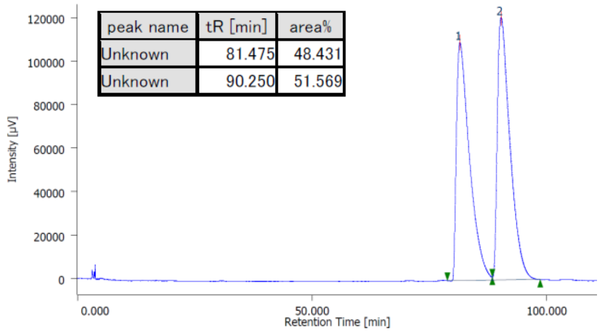

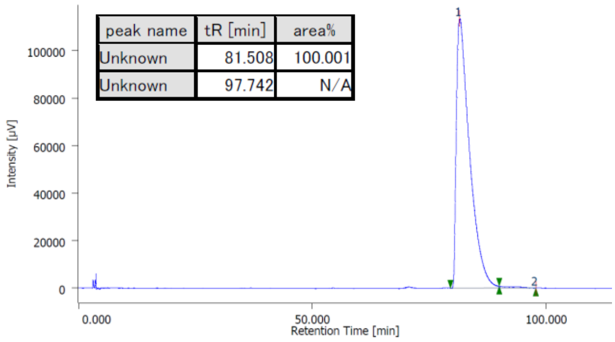

(*S*)-2-((*R*)-2-Ethyl-5-oxo-2,5-dihydrofuran-2-yl)chroman-4-one (5a) Prepared by the general procedure and isolated by preparative thin layer chromatography (Et_2O/petroleum ether = 3/1) as a yellow oil (43 mg, 70%, dr = 2.8:1). ^1H NMR (400 MHz, $CDCl_3$): δ 7.87 (dd, *J* = 8.0 Hz, 2.0 Hz, 1H), 7.55-7.45 (m, 2H), 7.05 (dd, *J* = 7.2 Hz, 7.2 Hz, 1H), 6.99 (d, *J* = 8.4 Hz, 1H), 6.24 (d, *J* = 5.6 Hz, 1H), 4.52 (dd, *J* = 13.2 Hz, 2.8 Hz, 1H), 2.85 (dd, *J* = 16.4 Hz, 13.6 Hz, 1H), 2.70 (dd, *J* = 16.4 Hz, 2.8 Hz, 1H), 2.10 (m, 2H), 0.90 (t, *J* = 7.2 Hz, 3H); ^{13}C NMR (100 MHz, $CDCl_3$): δ 190.58, 171.66, 160.50, 155.56, 136.30, 127.03, 123.40, 122.10, 120.80, 117.81, 90.57, 79.19, 37.95, 24.90, 7.04; IR (KBr): 3082, 3017, 2976, 2938, 2883, 1768, 1694, 1607, 1578, 1473, 1464, 1404, 1372, 1321, 1306, 1230, 1213, 1177, 1149, 1115, 1087, 1065, 1035, 995, 978, 959, 938, 919, 894, 851, 821, 765 cm^{-1}; HRMS (ESI$^+$) $[M+Na]^+$ found 281.0786, $C_{15}H_{14}O_4Na$ requires 281.0784; $[\alpha]_D^{25}$ +166 (*c* 0.05, $CHCl_3$); Enantiomeric excess was determined to be 99.7% ee by chiral HPLC analysis. HPLC: CHIRALCEL OD-H (ϕ 0.46 cm x 25 cm), 2-propanol/n-hexane = 1/9, flow rate 1.0 mL/min, detection 254 nm, tR = 42.3 min.

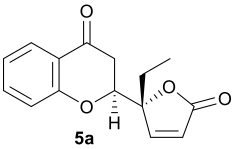

5a

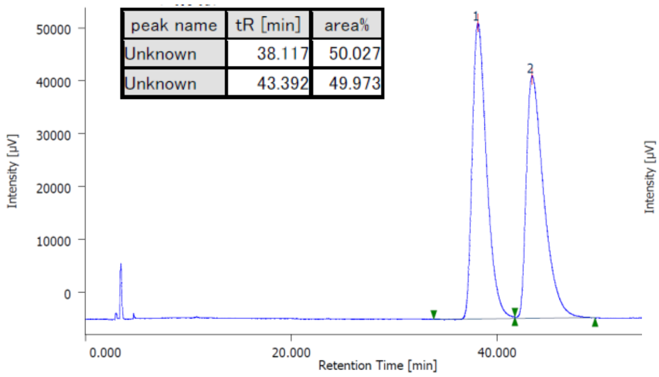

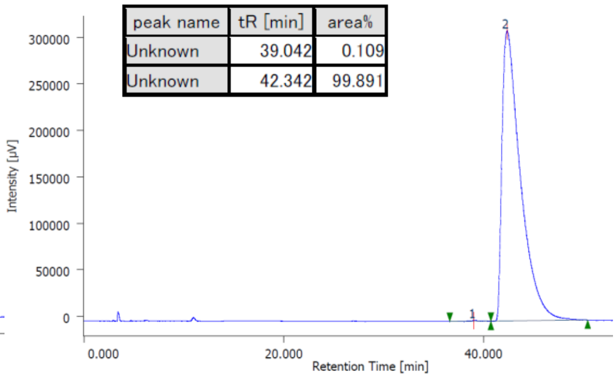

(*S*)-8-Bromo-2-((*R*)-2-ethyl-5-oxo-2,5-dihydrofuran-2-yl)chroman-4-one (5b) Prepared by the general procedure and isolated by chromatography on silica gel (Et_2O/petroleum ether = 3/1) as a white solid (60 mg, 74%, dr = 3.7:1). Melting point: 151 °C–153 °C; ^1H NMR (400 MHz, $CDCl_3$): δ 7.83 (dd, *J* = 7.6 Hz, 1.2 Hz, 1H), 7.74 (dd, *J* = 7.6 Hz, 1.6 Hz, 1H), 7.55 (d, *J* = 5.6 Hz, 1H), 6.95 (dd, *J* = 8.0 Hz, 8.0 Hz, 1H), 6.27 (d, *J* = 5.6 Hz, 1H), 4.55 (dd, *J* = 13.6 Hz, 2.8 Hz, 1H), 2.89 (dd, *J* = 16.8 Hz, 13.6 Hz, 1H), 2.75 (dd, *J* = 16.8 Hz, 3.2 Hz, 1H), 2.16 (m, 2H), 0.92 (t, *J* = 7.2 Hz, 3H); ^{13}C NMR (100 MHz, $CDCl_3$): δ 189.80, 171.51, 156.85, 155.75, 139.38, 126.34, 123.49, 122.83, 122.02, 111.76, 90.41, 79.97, 37.45, 24.79, 7.13; IR (KBr): 3095, 2981, 2945, 2915, 2848, 1758, 1715, 1687, 1651, 1633, 1593, 1556, 1507, 1487, 1463, 1455, 1435, 1415, 1374, 1338, 1328, 1290, 1264, 1238, 1199, 1171, 1149, 1131, 1092, 1062, 1027, 1013, 961, 916, 860, 837, 821, 786, 757, 727 cm^{-1}; HRMS (ESI$^+$) $[M+Na]^+$ found 358.9890, $C_{15}H_{13}O_4BrNa$ requires 358.9889; $[\alpha]_D^{25}$ +144 (*c* 0.045, $CHCl_3$); Enantiomeric excess was determined to be >99% ee by chiral HPLC analysis. HPLC analysis. HPLC: CHIRALCEL IB (ϕ 0.46 cm x 25 cm), 2-propanol/n-hexane = 5/95, flow rate 1.0 mL/min, detection 254 nm, tR = 67.4 min.

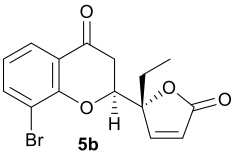

5b

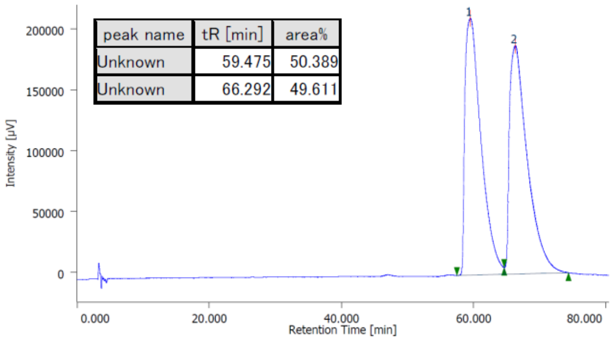

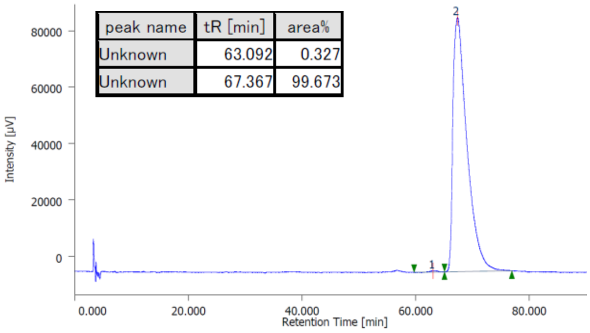

3. Crystal structure analysis

Solid state structure of **3a**.

Single crystals of **3a** were obtained by vapor diffusion from dichloromethane/hexane at RT. A suitable crystal was selected and the sample was measured on a Rigaku R-AXIS RAPIS II diffractometer using multi-layer mirror monochromated Cu-Kα radiation. The data were collected at 93 K. Refined structure and crystallographic parameters are summarized in Supplementary Table S1 and Supplementary Fig. S1. CCDC 1988595 contains the supplementary

crystallographic data for **3a**.

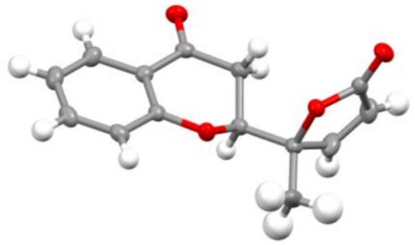

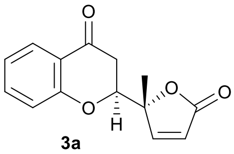

3a

Figure S1 Structure of **3a** in the solid state. Color code: red: oxygen; gray: carbon; white: hydrogen.

Table S1 Selected crystal data of **3a**.

Empirical formula	$C_{14}H_{12}O_4$
Formula weight	244.24
Crystal system	monoclinic
Crystal size/mm^3	0.2 × 0.2 × 0.05
Space group	P21
Lattice Parameters	
a	8.10250(10) Å
b	6.64010(10) Å
c	11.71340(10) Å
α	90 °
β	102.4050(10) °
γ	90 °
V	615.484(13) Å^3
Z	2
R_1	0.0273
wR_2	0.0709
D_{calc}	1.318 g/cm^3
F(000)	256.0

Solid state structure of **3g**.

Single crystals of **3g** were obtained by vapor diffusion from dichloromethane/hexane at RT. A suitable crystal was selected and the sample was measured on a Rigaku R-AXIS RAPIS II diffractometer using multi-layer mirror monochromated Cu-Kα radiation. The data were collected at 93 K. Refined structure and crystallographic parameters are summarized in Supplementary Table S2 and Supplementary Fig. S2. CCDC 1988596 contains the supplementary crystallographic data for **3g**.

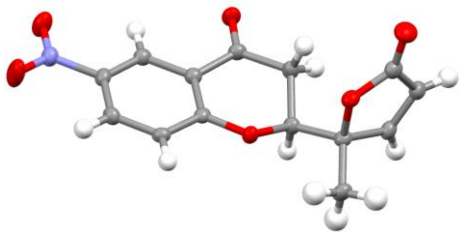

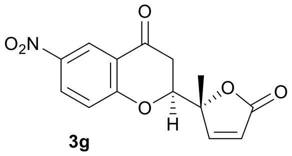

3g

Figure S2 Structure of **3g** in the solid state. Color code: red: oxygen; light blue: nitrogen; gray: carbon; white: hydrogen.

Table S2 Selected crystal data of **3g**.

Empirical formula	$C_{28}H_{22}N_2O_{12}$
Formula weight	578.47
Crystal system	monoclinic
Crystal size/mm^3	0.1 × 0.02 × 0.02
Space group	P21
Lattice Parameters	
a	12.4535(2) Å
b	6.54820(10) Å
c	15.9054(3) Å
α	90 °
β	105.099(2) °
γ	90 °
V	1252.28(4) Å^3
Z	2
R_1	0.0434
wR_2	0.0942
D_{calc}	1.534 g/cm^3
F(000)	600.0

4. Appendages

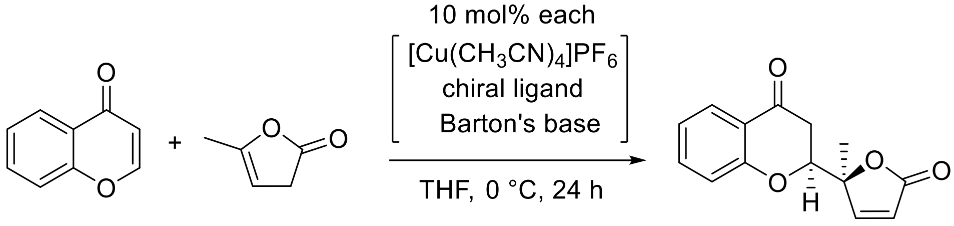

***syn*-3a**

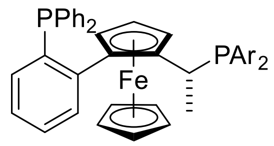

Ar = 3,5-$(CF_3)_2$-C_6H_3

(*R*,*R*p)-Walphos-type

yield 72%
dr > 20:1
ee (*syn*) = –10%

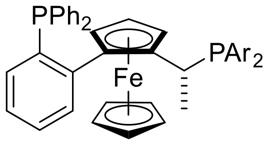

Ar = 3,5-Me_2-C_6H_3

(*R*,*R*p)-Walphos-type

yield 39%
dr = 16:1
ee (*syn*) = –10%

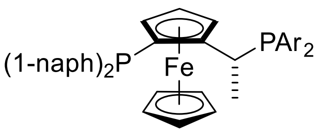

Ar = 3,5-Me_2-C_6H_3

(*R*,*S*)-JosiPhos-type

yield 75%
dr > 20:1
ee (*syn*) = –52%

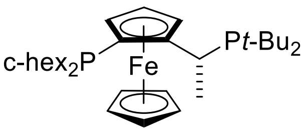

(*R*,*S*)-JosiPhos-type

yield 79%
dr > 20:1
ee (*syn*) = –50%

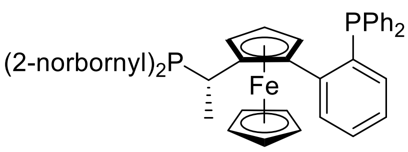

(*S*,*S*p)-Walphos-type

yield 63%
dr > 20:1
ee (*syn*) = 23%

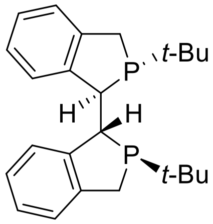

(1*R*,1'*R*,2*S*,2'*S*)-DuanPhos

yield < 5%

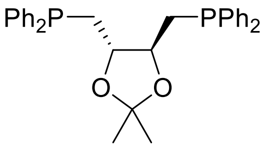

(*S*,*S*)-DIOP

yield 90%
dr > 20:1
ee (*syn*) = 0%

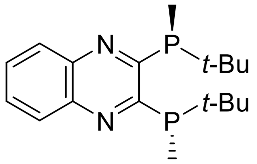

(S,S)-QuinoxP*

yield < 5%

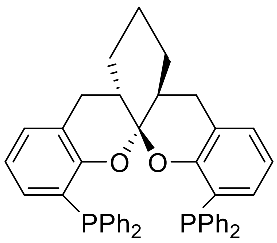

(*R*,*R*,*R*)-(+)-Ph-SKP

yield 51%
dr > 20:1
ee (*syn*) = –36%

yield: combined NMR yield of both isomers

Figure S3 Ineffective ligands suffering from low selectivity and conversion.

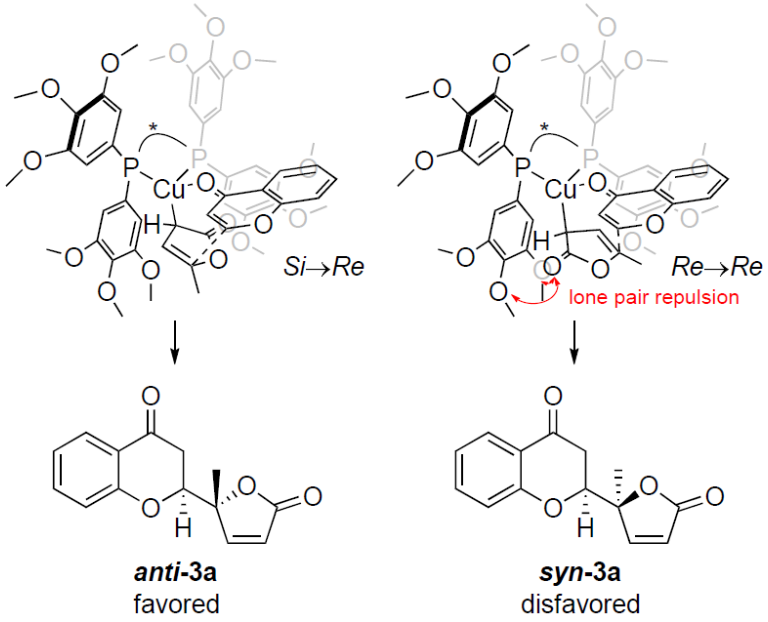

Figure S4 Proposed TS via C-bound form.

5. References

(1) Trost, B. M.; Gnanamani, E.; Kalnmals, C. A.; Hung, C.; Tracy, J. S. *J. Am. Chem. Soc.* **2019**, *141*, 1489−1493.
(2) Ding, C.; Yu, Y.; Yu, Q.; Xie, Z.; Zhou, Y.; Zhou, J.; Liang, G.; Song, Z. *ChemCatChem*, **2018**, *10*, 5397−5410.

6. NMR Spectra of New Compounds (^{1}H and ^{13}C NMR spectra)

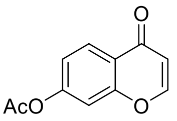

1j

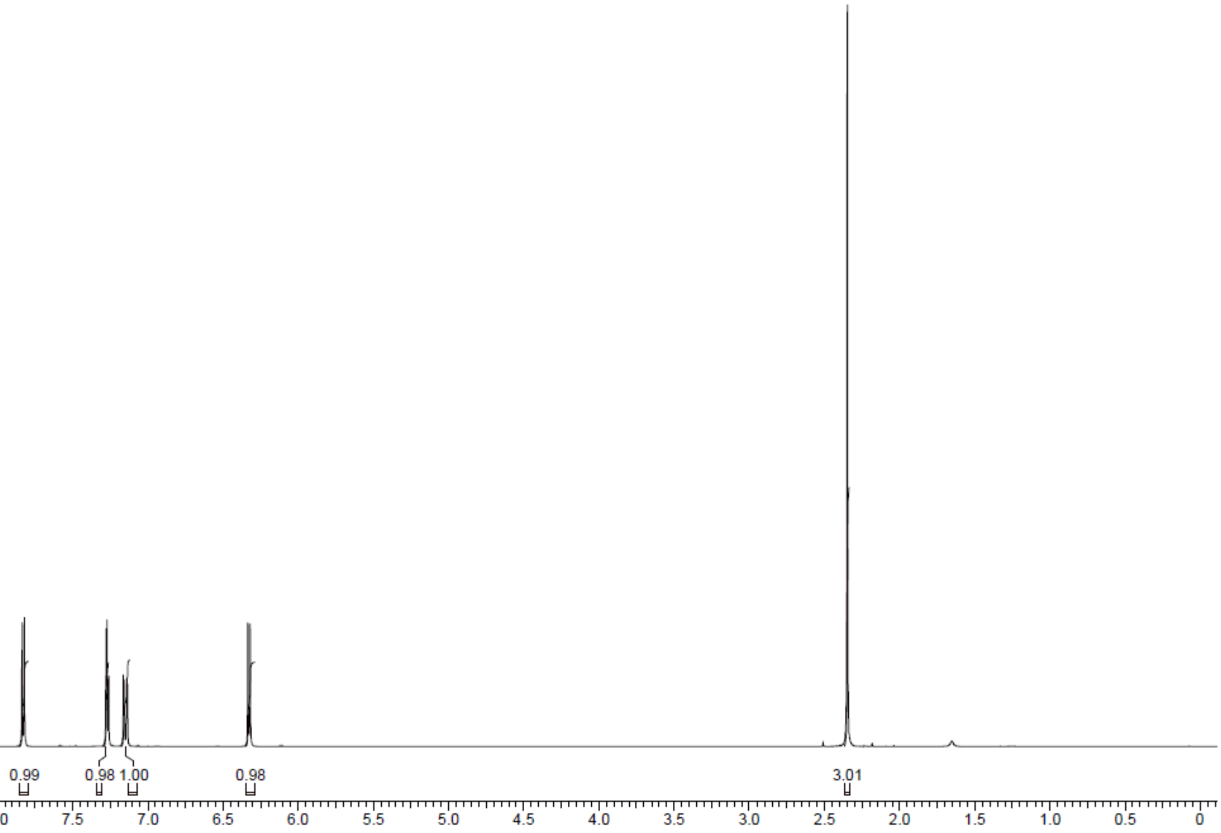

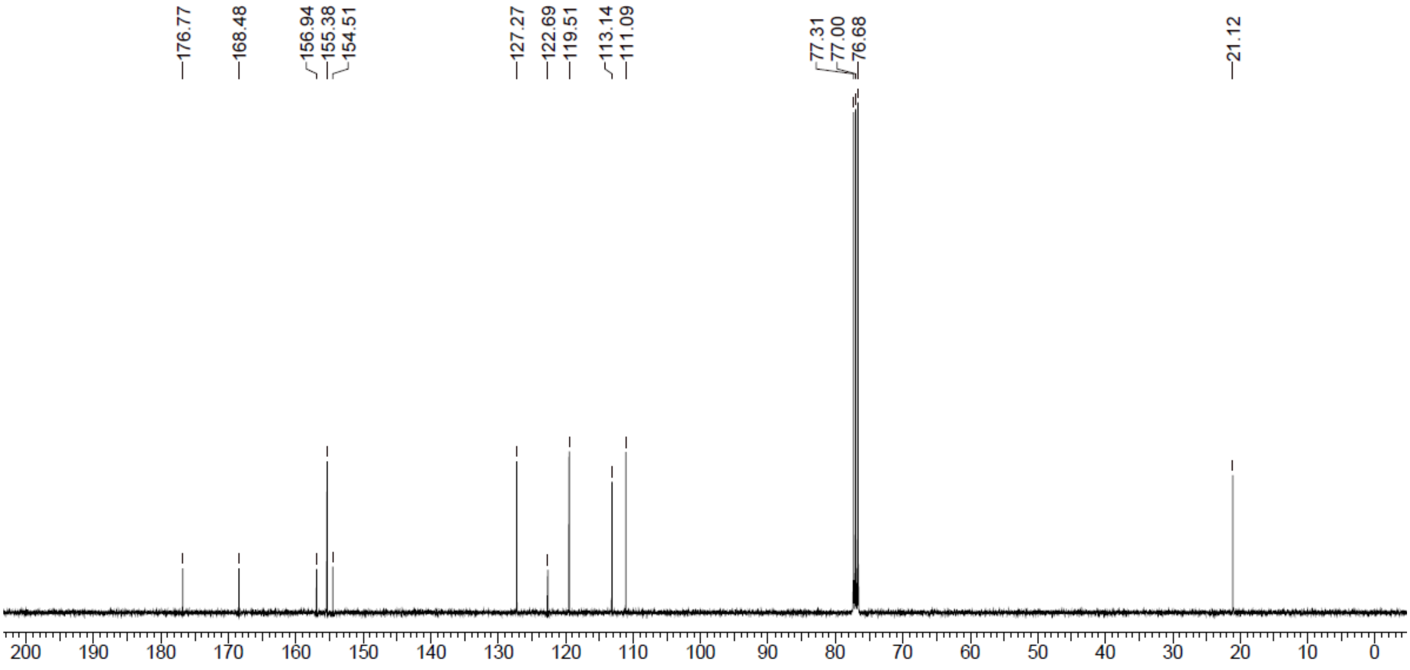

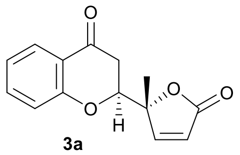

3a

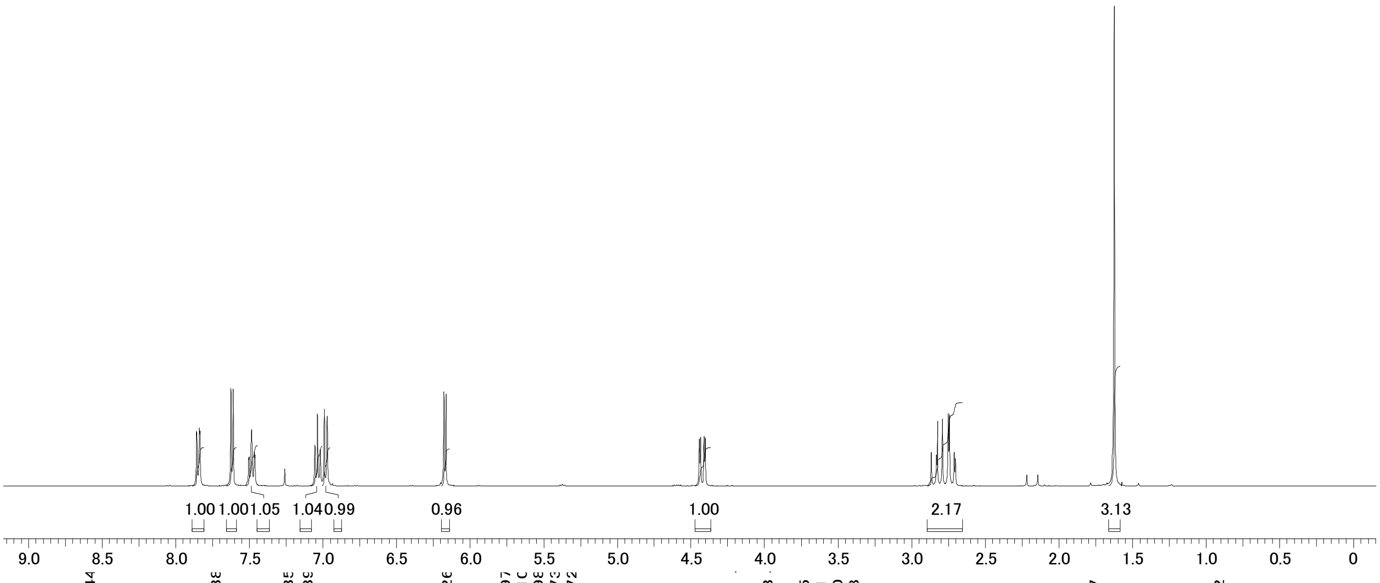

1.00 1.00 1.05 1.04 0.99
0.96
1.00
2.17
3.13
9.0 8.5 8.0 7.5 7.0 6.5 6.0 5.5 5.0 4.5 4.0 3.5 3.0 2.5 2.0 1.5 1.0 0.5 0

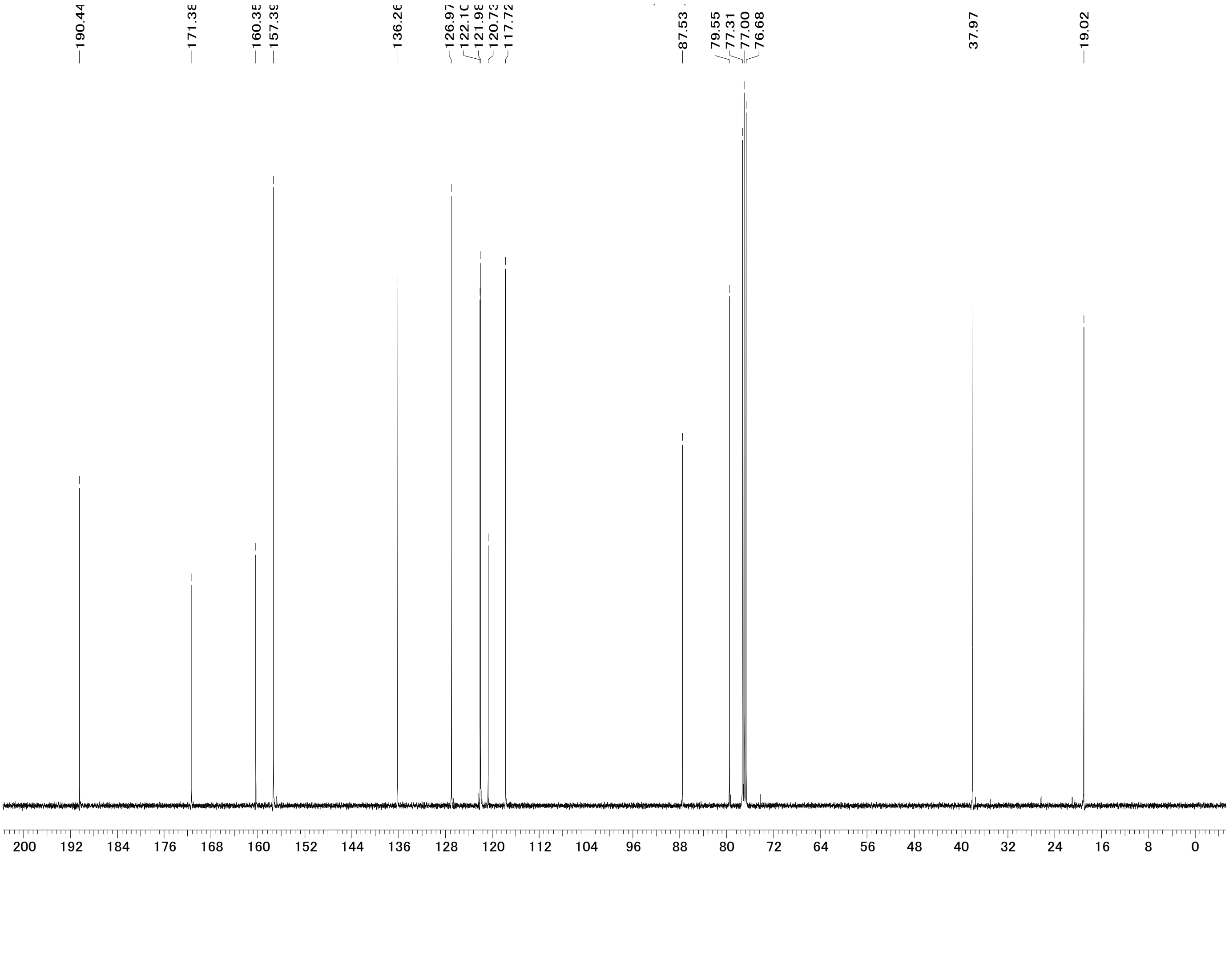

190.44
171.38
160.35
157.39
136.26
126.97
122.10
121.98
120.73
117.72
87.53
79.55
77.31
77.00
76.68
37.97
19.02
200 192 184 176 168 160 152 144 136 128 120 112 104 96 88 80 72 64 56 48 40 32 24 16 8 0

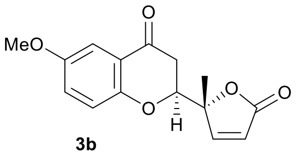
MeO
O
O
H
O
O

3b

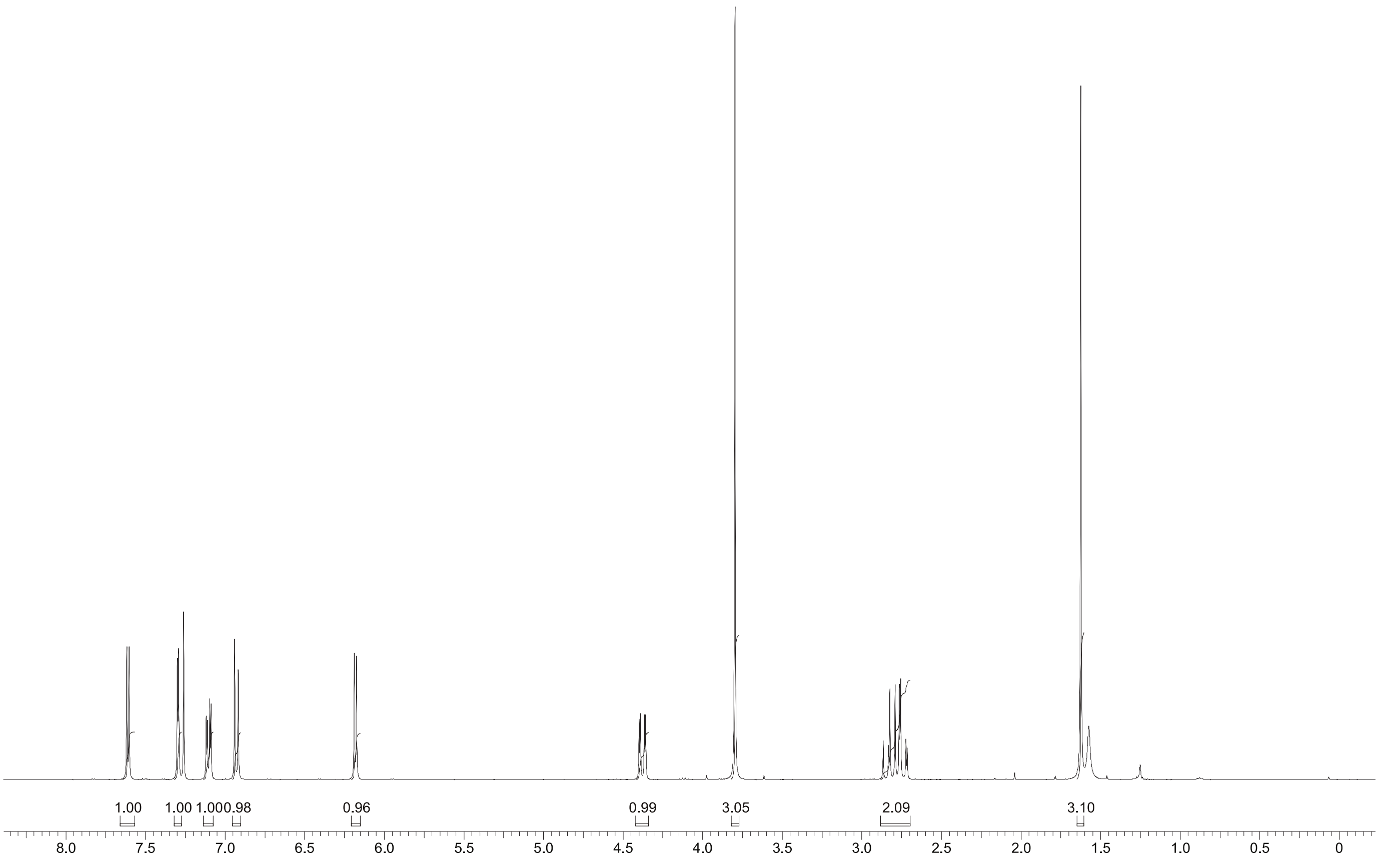
1.00
1.00
1.00
0.98
0.96
0.99
3.05
2.09
3.10
8.0 7.5 7.0 6.5 6.0 5.5 5.0 4.5 4.0 3.5 3.0 2.5 2.0 1.5 1.0 0.5 0

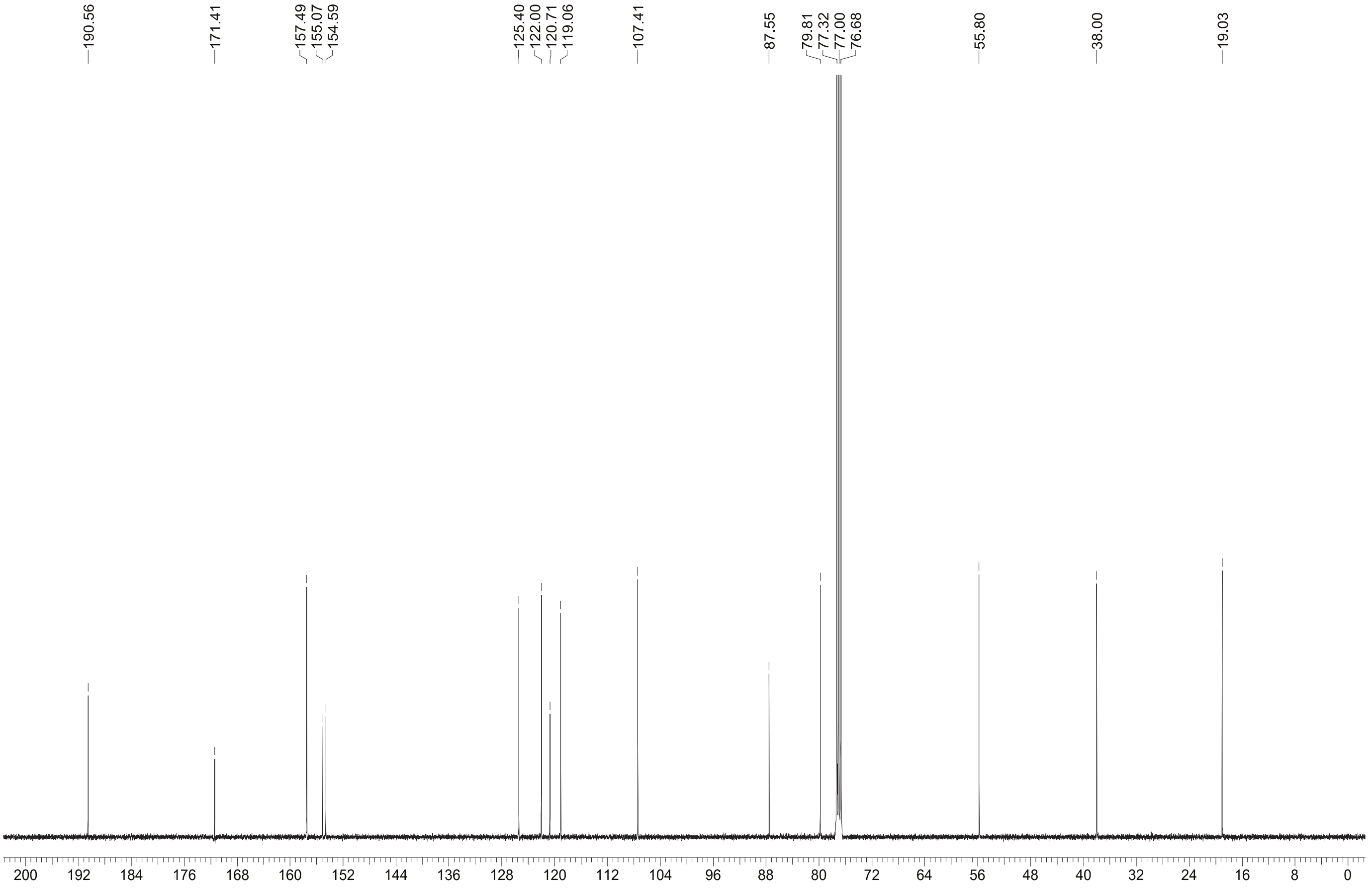
190.56
171.41
157.49
155.07
154.59
125.40
122.00
120.71
119.06
107.41
87.55
79.81
77.32
77.00
76.68
55.80
38.00
19.03
200 192 184 176 168 160 152 144 136 128 120 112 104 96 88 80 72 64 56 48 40 32 24 16 8 0

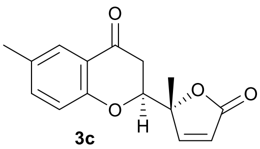

3c

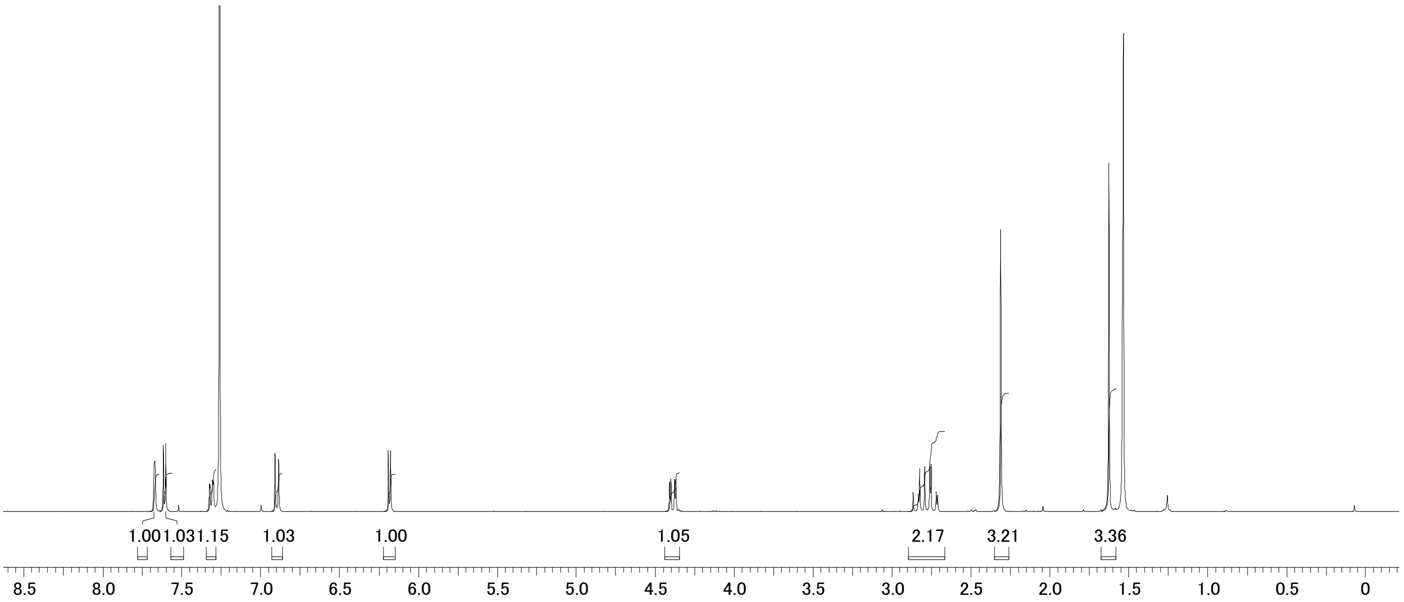
1.00 1.03 1.15 1.03 1.00 1.05 2.17 3.21 3.36
8.5 8.0 7.5 7.0 6.5 6.0 5.5 5.0 4.5 4.0 3.5 3.0 2.5 2.0 1.5 1.0 0.5 0

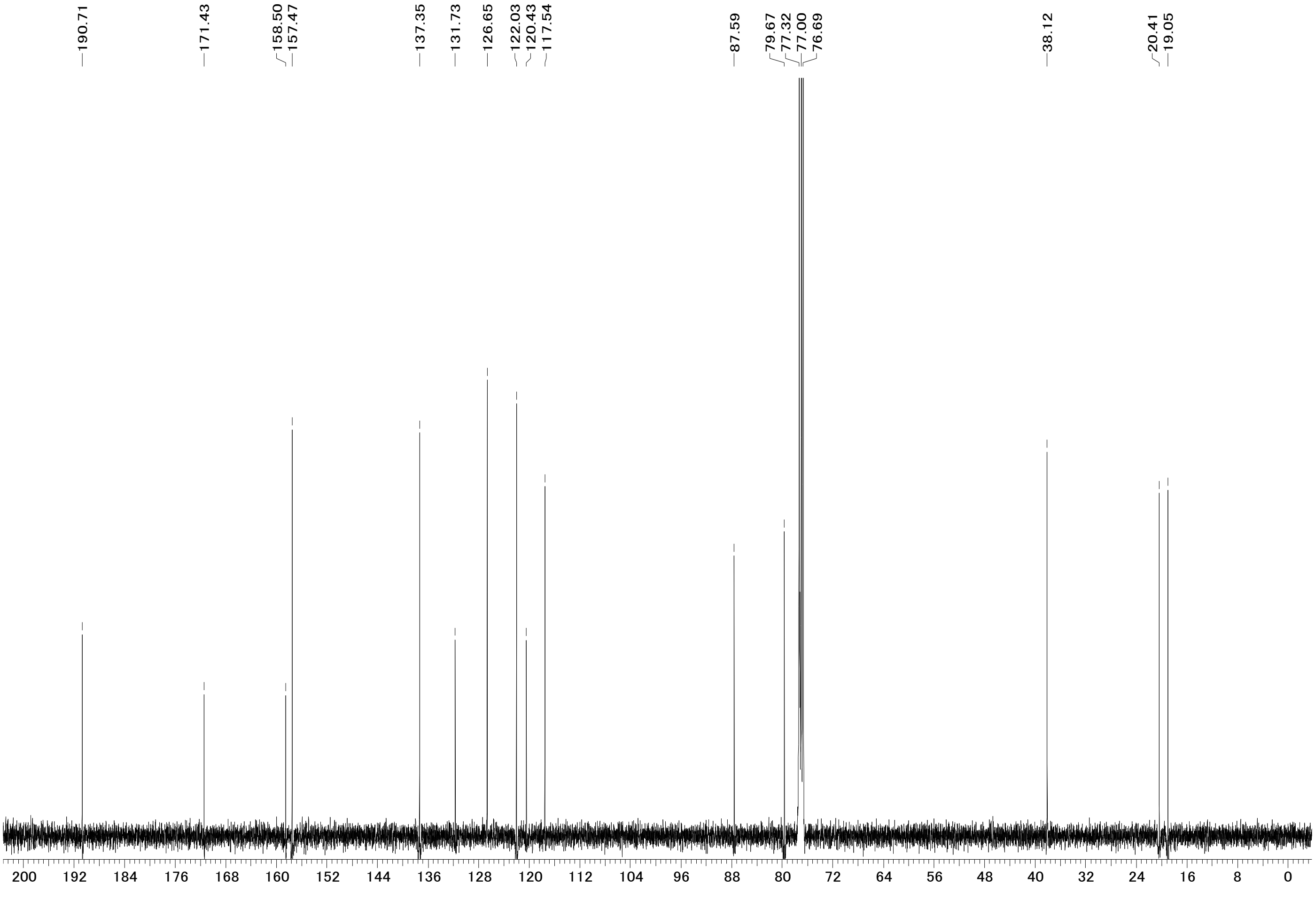
190.71
171.43
158.50
157.47
137.35
131.73
126.65
122.03
120.43
117.54
87.59
79.67
77.32
77.00
76.69
38.12
20.41
19.05
200 192 184 176 168 160 152 144 136 128 120 112 104 96 88 80 72 64 56 48 40 32 24 16 8 0

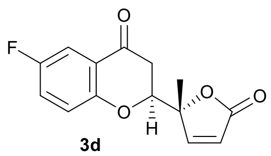
F
O
O
O
O
H

3d

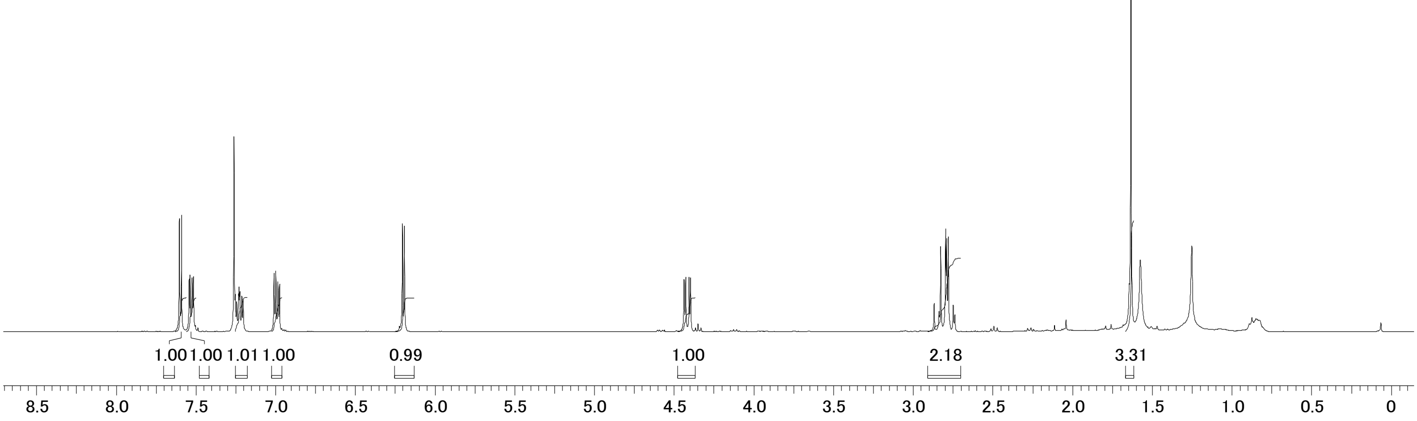
1.00 1.00 1.01 1.00
0.99
1.00
2.18
3.31
8.5 8.0 7.5 7.0 6.5 6.0 5.5 5.0 4.5 4.0 3.5 3.0 2.5 2.0 1.5 1.0 0.5 0

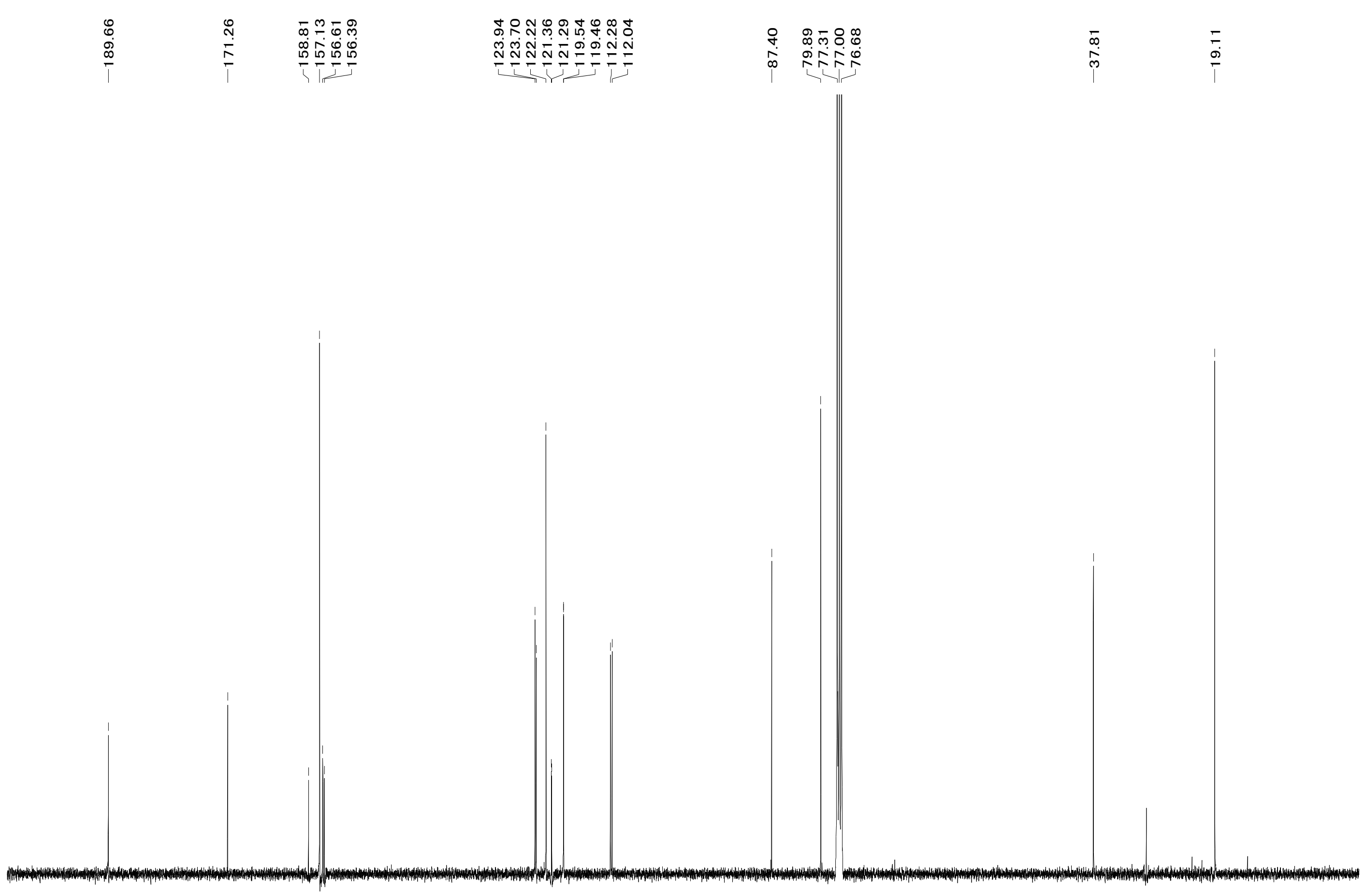
189.66
171.26
158.81 157.13 156.61 156.39
123.94 123.70 122.22 121.36 121.29 119.54 119.46 112.28 112.04
87.40
79.89 77.31 77.00 76.68
37.81
19.11
200 192 184 176 168 160 152 144 136 128 120 112 104 96 88 80 72 64 56 48 40 32 24 16 8 0

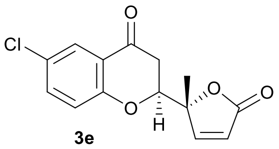
Cl
O
O
O
O
H

3e

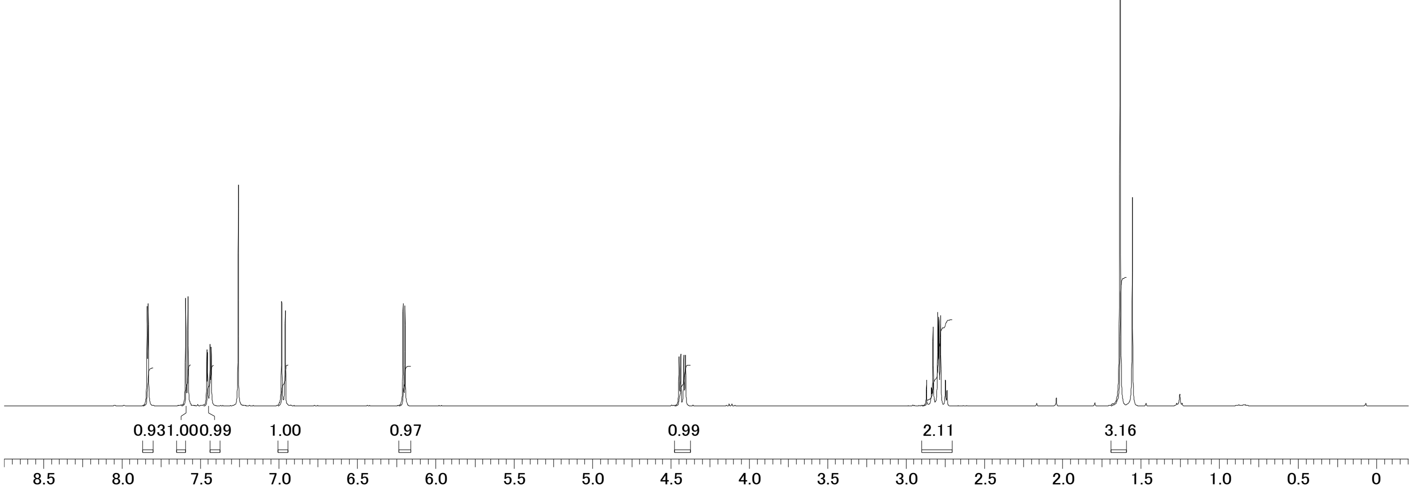
0.93
1.00
0.99
1.00
0.97
0.99
2.11
3.16
8.5 8.0 7.5 7.0 6.5 6.0 5.5 5.0 4.5 4.0 3.5 3.0 2.5 2.0 1.5 1.0 0.5 0

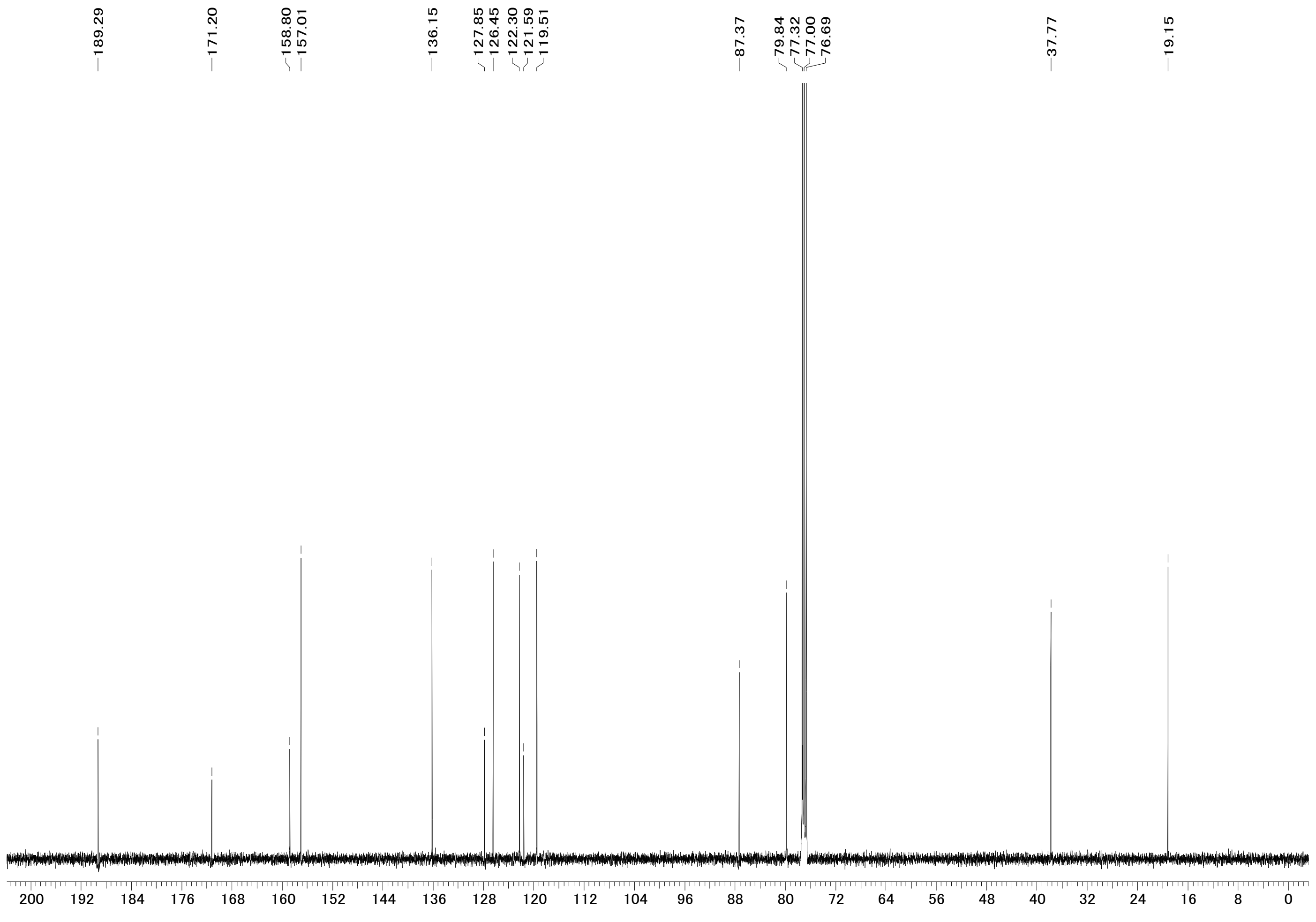
189.29
171.20
158.80
157.01
136.15
127.85
126.45
122.30
121.59
119.51
87.37
79.84
77.32
77.00
76.69
37.77
19.15
200 192 184 176 168 160 152 144 136 128 120 112 104 96 88 80 72 64 56 48 40 32 24 16 8 0

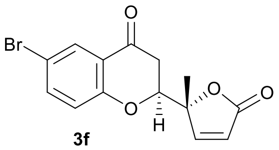
Br
O
O
O
O
H

3f

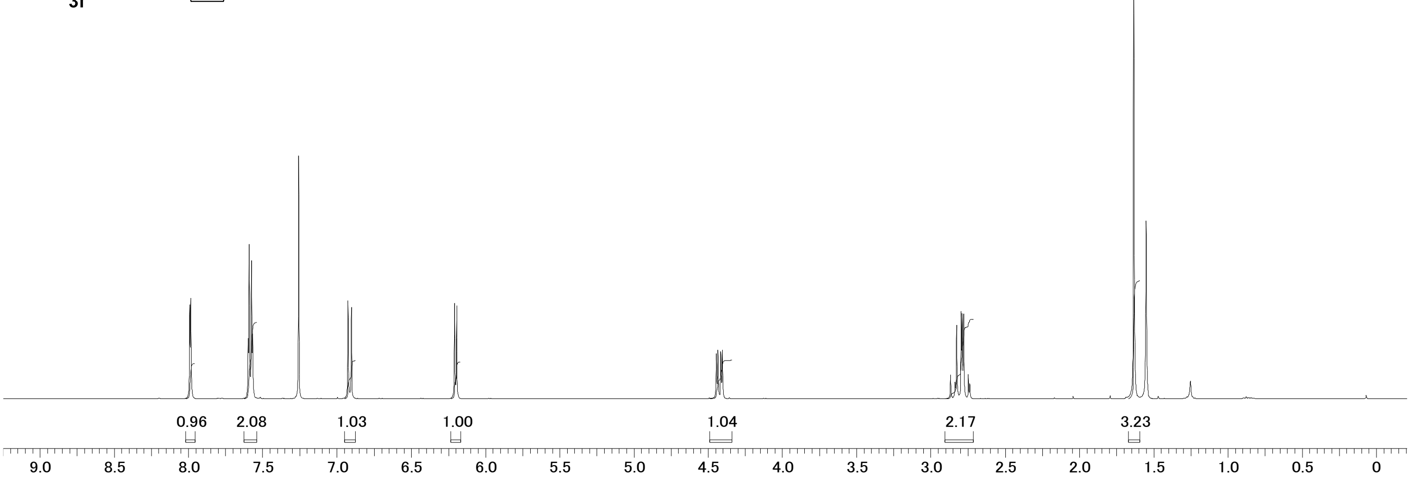
0.96
2.08
1.03
1.00
1.04
2.17
3.23
9.0
8.5
8.0
7.5
7.0
6.5
6.0
5.5
5.0
4.5
4.0
3.5
3.0
2.5
2.0
1.5
1.0
0.5
0

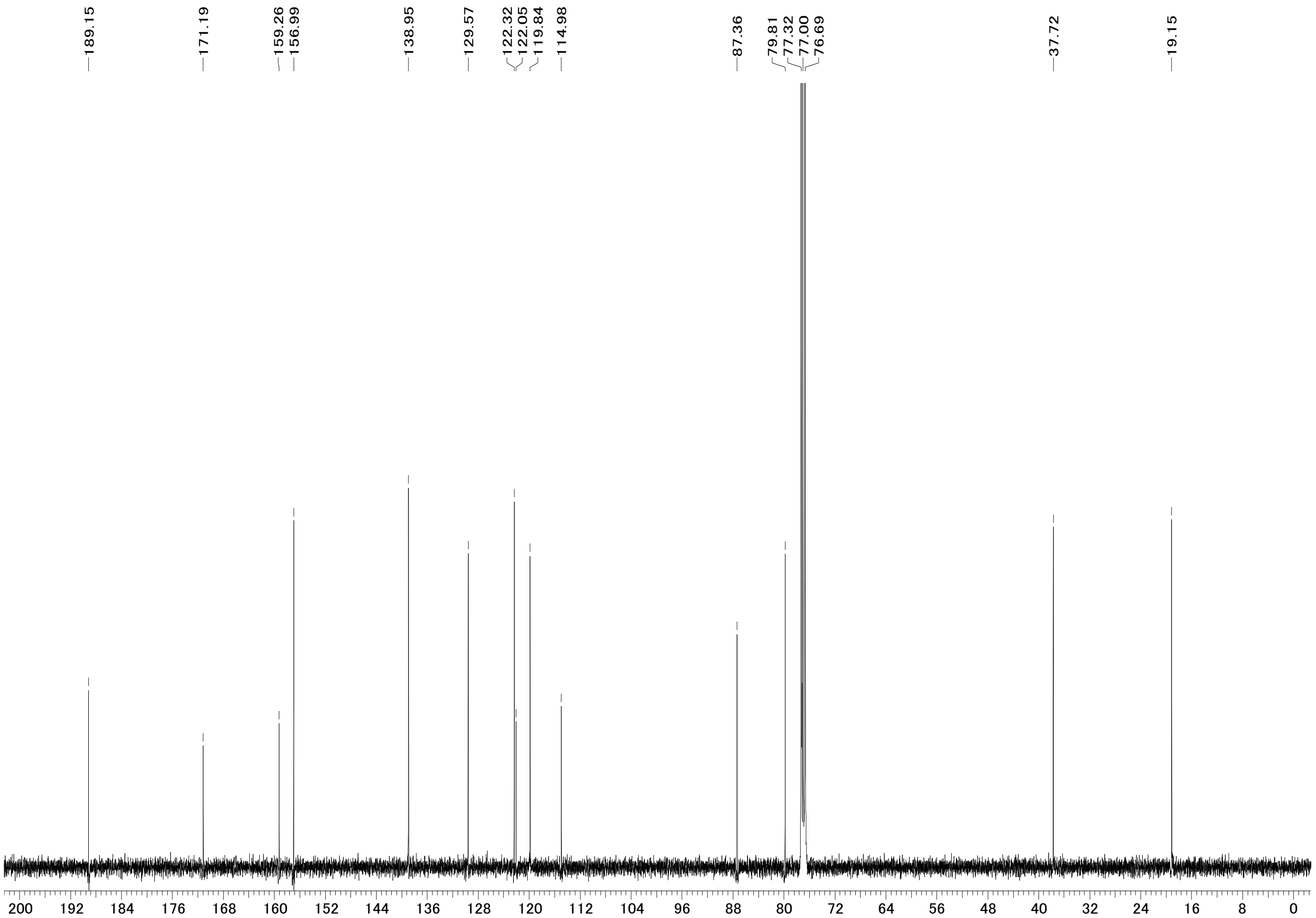
189.15
171.19
159.26
156.99
138.95
129.57
122.32
122.05
119.84
114.98
87.36
79.81
77.32
77.00
76.69
37.72
19.15
200
192
184
176
168
160
152
144
136
128
120
112
104
96
88
80
72
64
56
48
40
32
24
16
8
0

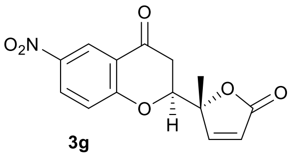

3g

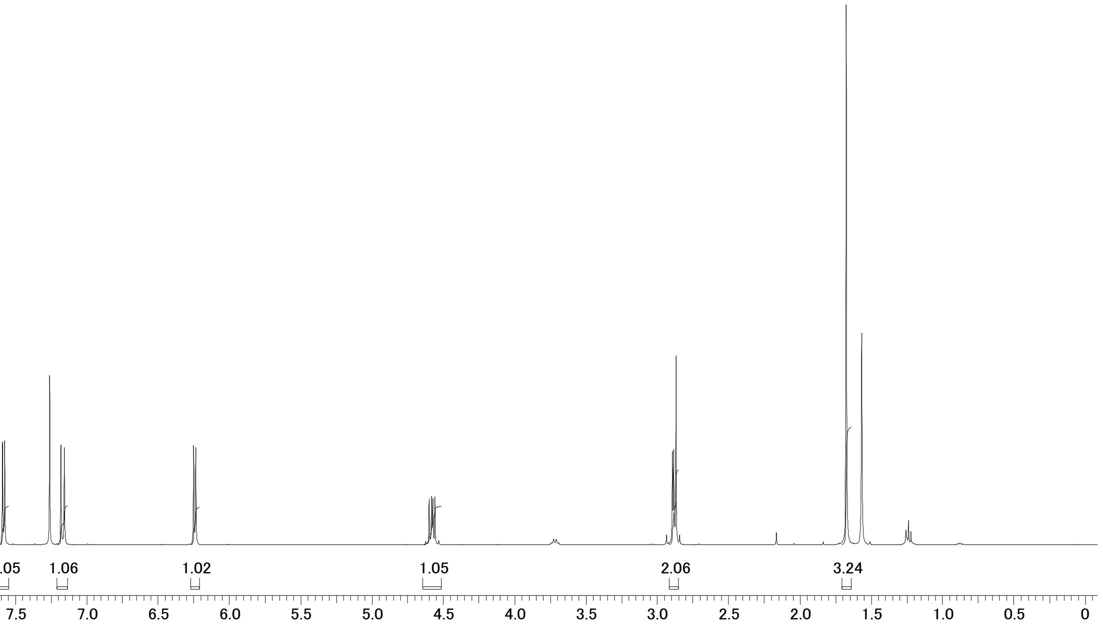

0.98
1.05
1.05
1.06
1.02
1.05
2.06
3.24
9.5 9.0 8.5 8.0 7.5 7.0 6.5 6.0 5.5 5.0 4.5 4.0 3.5 3.0 2.5 2.0 1.5 1.0 0.5 0

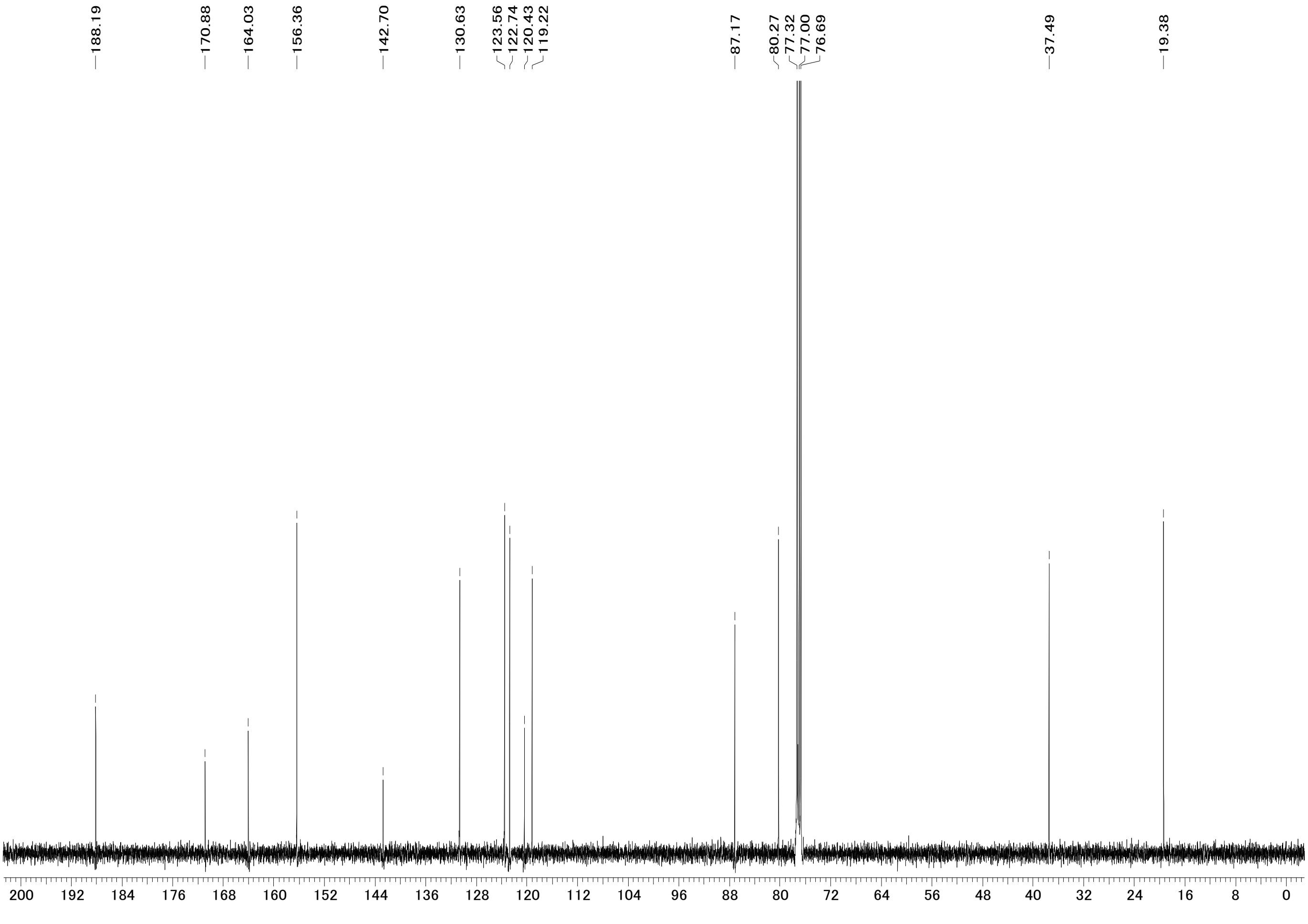

188.19
170.88
164.03
156.36
142.70
130.63
123.56
122.74
120.43
119.22
87.17
80.27
77.32
77.00
76.69
37.49
19.38
200 192 184 176 168 160 152 144 136 128 120 112 104 96 88 80 72 64 56 48 40 32 24 16 8 0

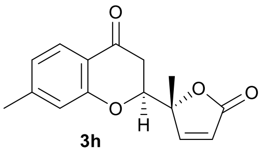

3h

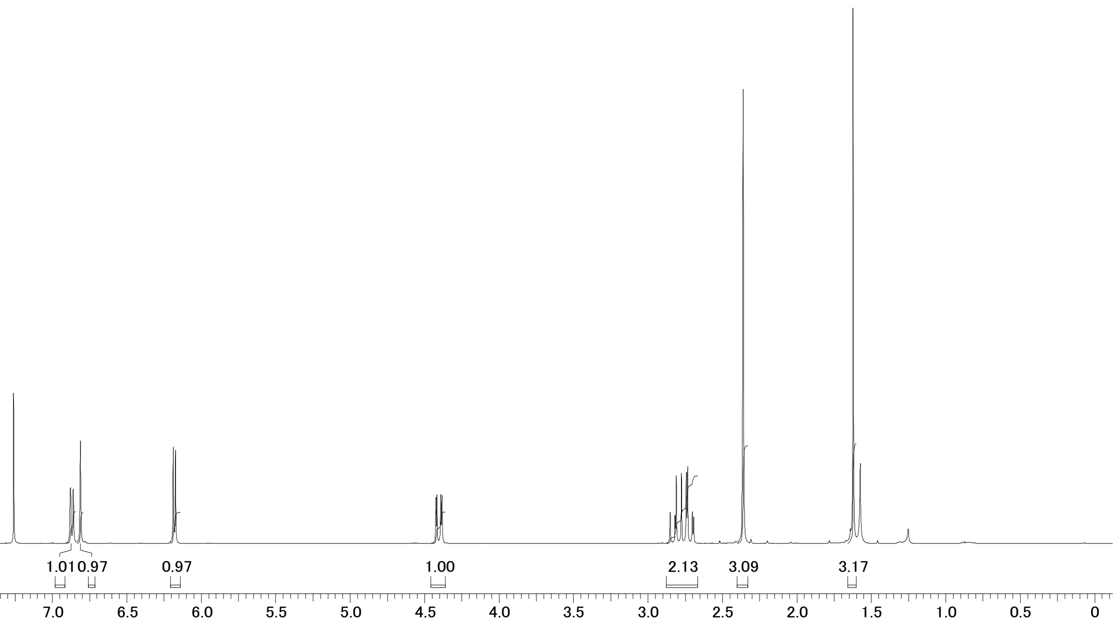
0.98
1.00
1.01
0.97
0.97
1.00
2.13
3.09
3.17
9.0
8.5
8.0
7.5
7.0
6.5
6.0
5.5
5.0
4.5
4.0
3.5
3.0
2.5
2.0
1.5
1.0
0.5
0

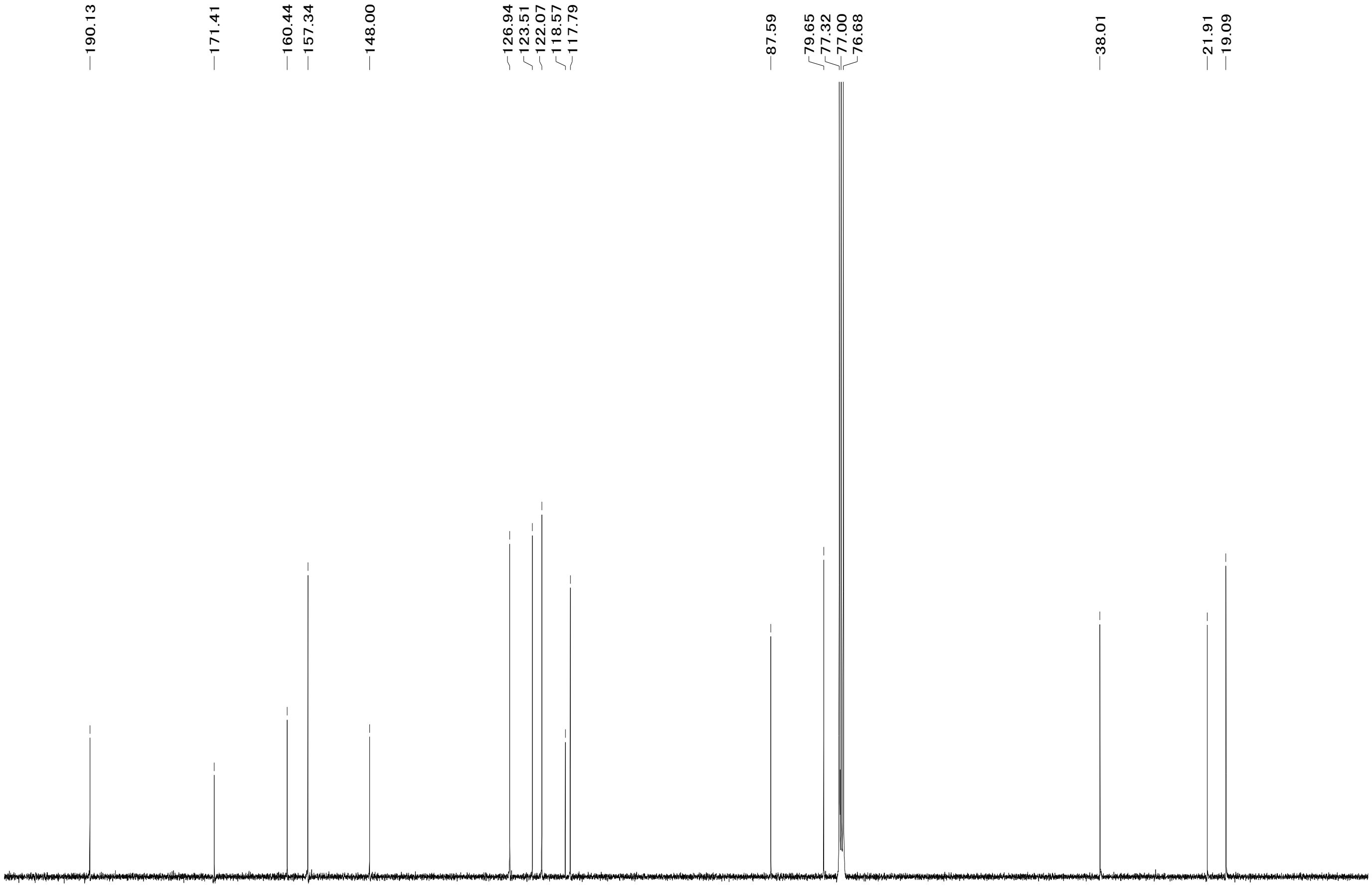
190.13
171.41
160.44
157.34
148.00
126.94
123.51
122.07
118.57
117.79
87.59
79.65
77.32
77.00
76.68
38.01
21.91
19.09
200
192
184
176
168
160
152
144
136
128
120
112
104
96
88
80
72
64
56
48
40
32
24
16
8
0

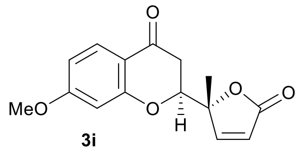
O
MeO
O
H
O
O

3i

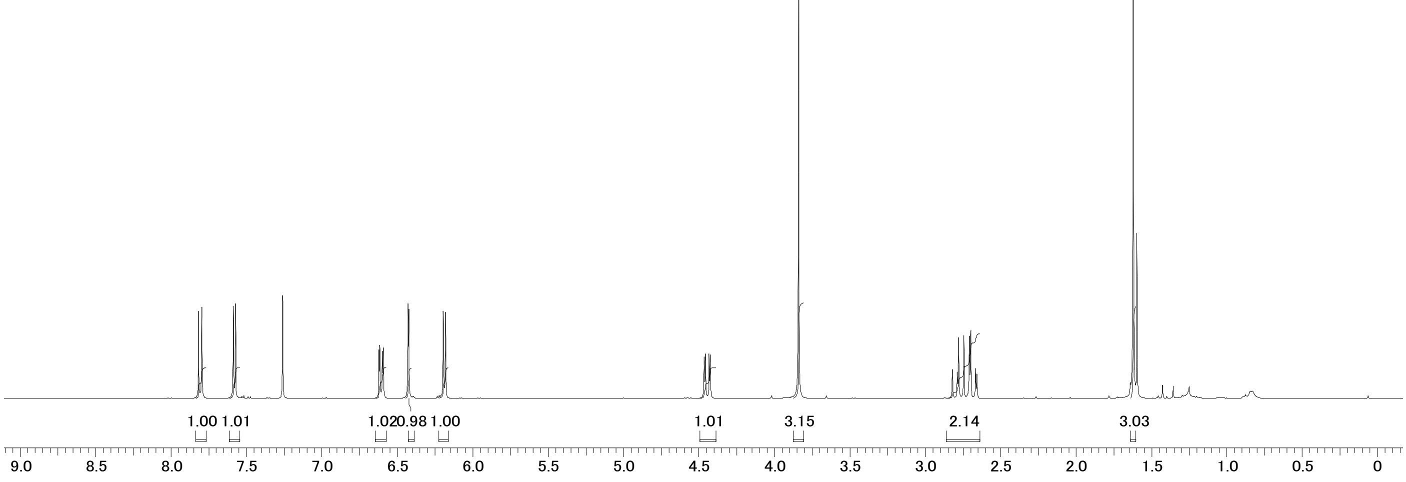
1.00 1.01 1.02 0.98 1.00 1.01 3.15 2.14 3.03
9.0 8.5 8.0 7.5 7.0 6.5 6.0 5.5 5.0 4.5 4.0 3.5 3.0 2.5 2.0 1.5 1.0 0.5 0

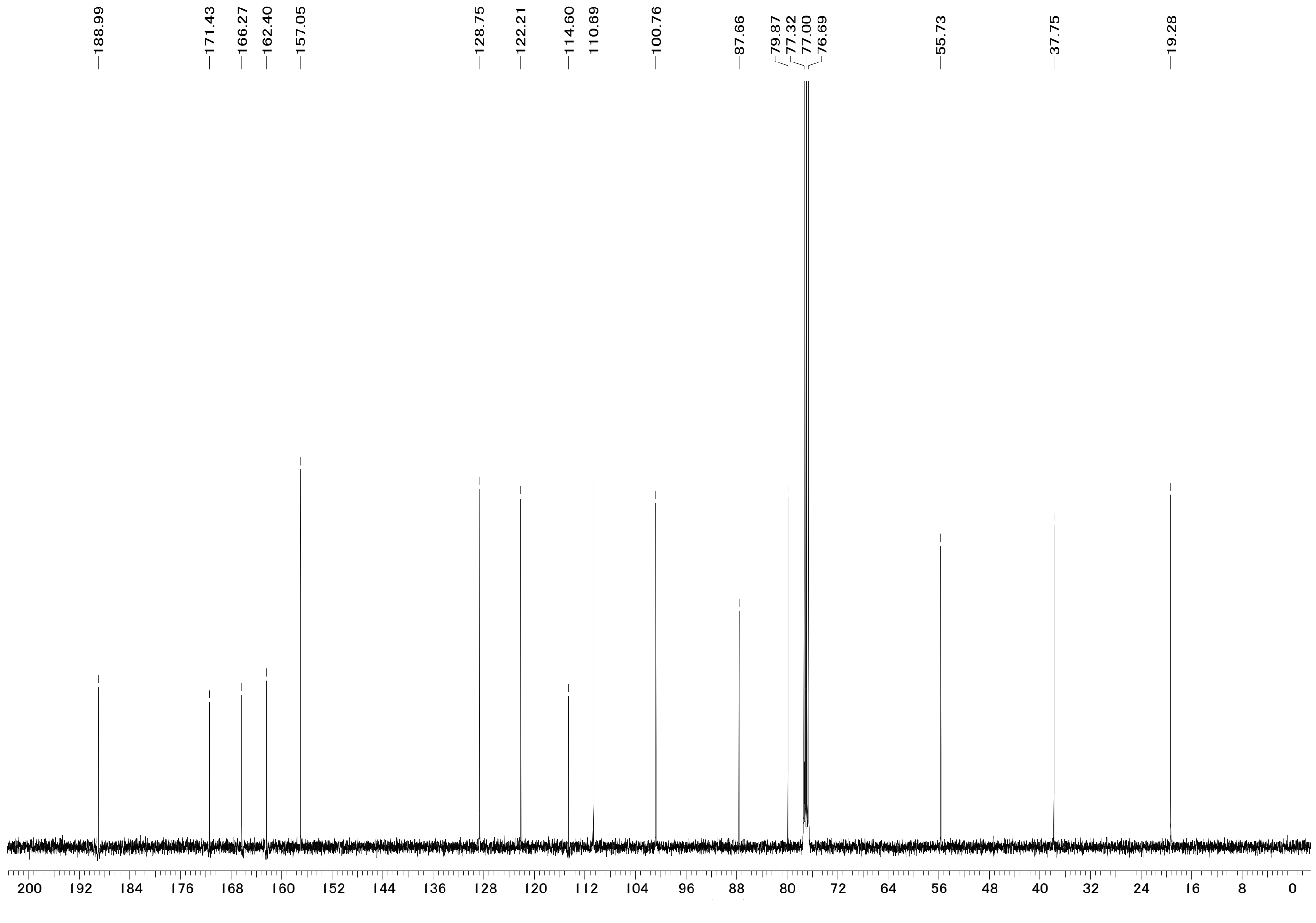
188.99
171.43
166.27
162.40
157.05
128.75
122.21
114.60
110.69
100.76
87.66
79.87
77.32
77.00
76.69
55.73
37.75
19.28
200 192 184 176 168 160 152 144 136 128 120 112 104 96 88 80 72 64 56 48 40 32 24 16 8 0

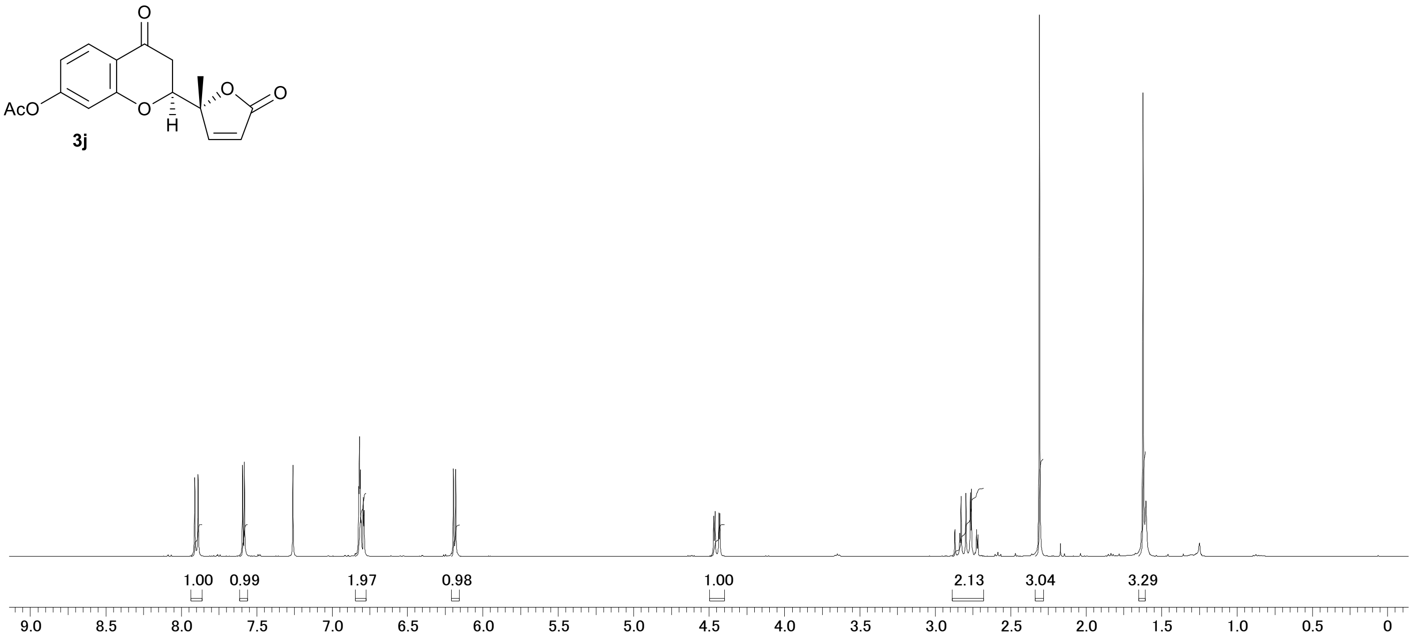
O
AcO
O
H
O
O
3j
1.00 0.99 1.97 0.98 1.00 2.13 3.04 3.29
9.0 8.5 8.0 7.5 7.0 6.5 6.0 5.5 5.0 4.5 4.0 3.5 3.0 2.5 2.0 1.5 1.0 0.5 0

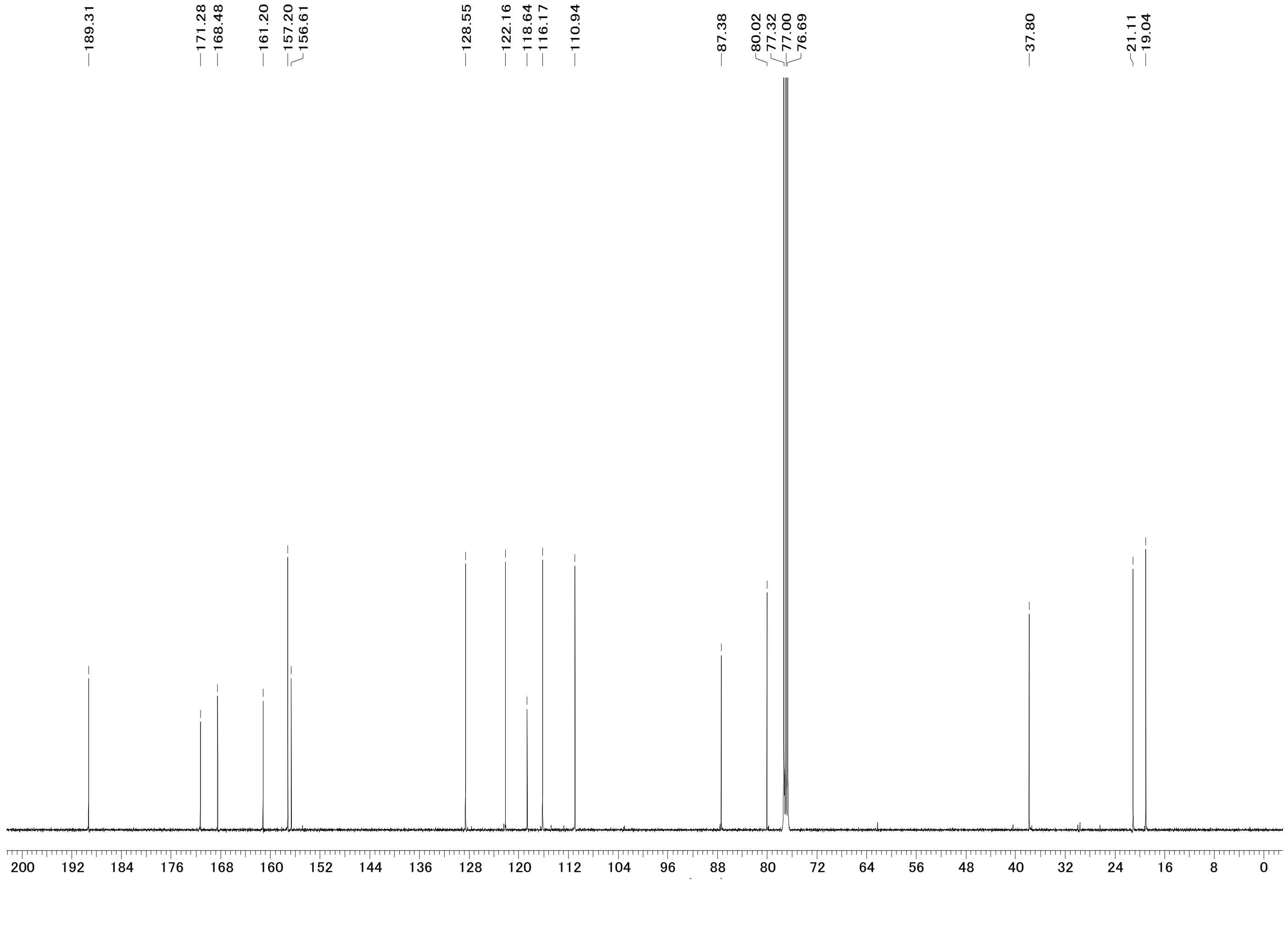
189.31
171.28
168.48
161.20
157.20
156.61
128.55
122.16
118.64
116.17
110.94
87.38
80.02
77.32
77.00
76.69
37.80
21.11
19.04
200 192 184 176 168 160 152 144 136 128 120 112 104 96 88 80 72 64 56 48 40 32 24 16 8 0

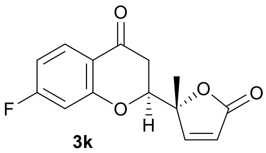
F
O
O
H
O
O

3k

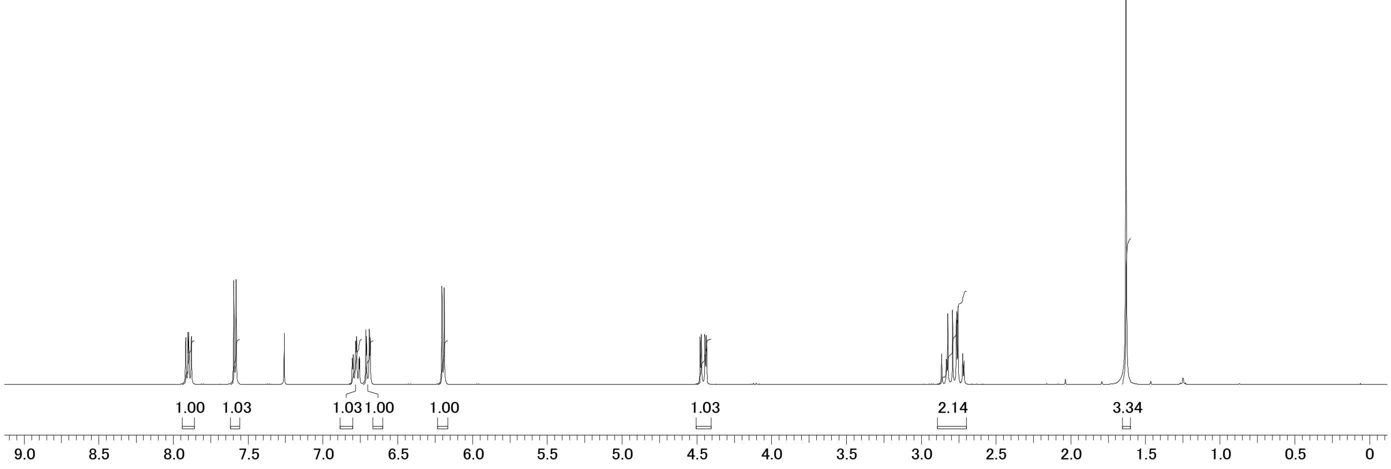
1.00 1.03 1.03 1.00 1.00 1.03 2.14 3.34
9.0 8.5 8.0 7.5 7.0 6.5 6.0 5.5 5.0 4.5 4.0 3.5 3.0 2.5 2.0 1.5 1.0 0.5 0

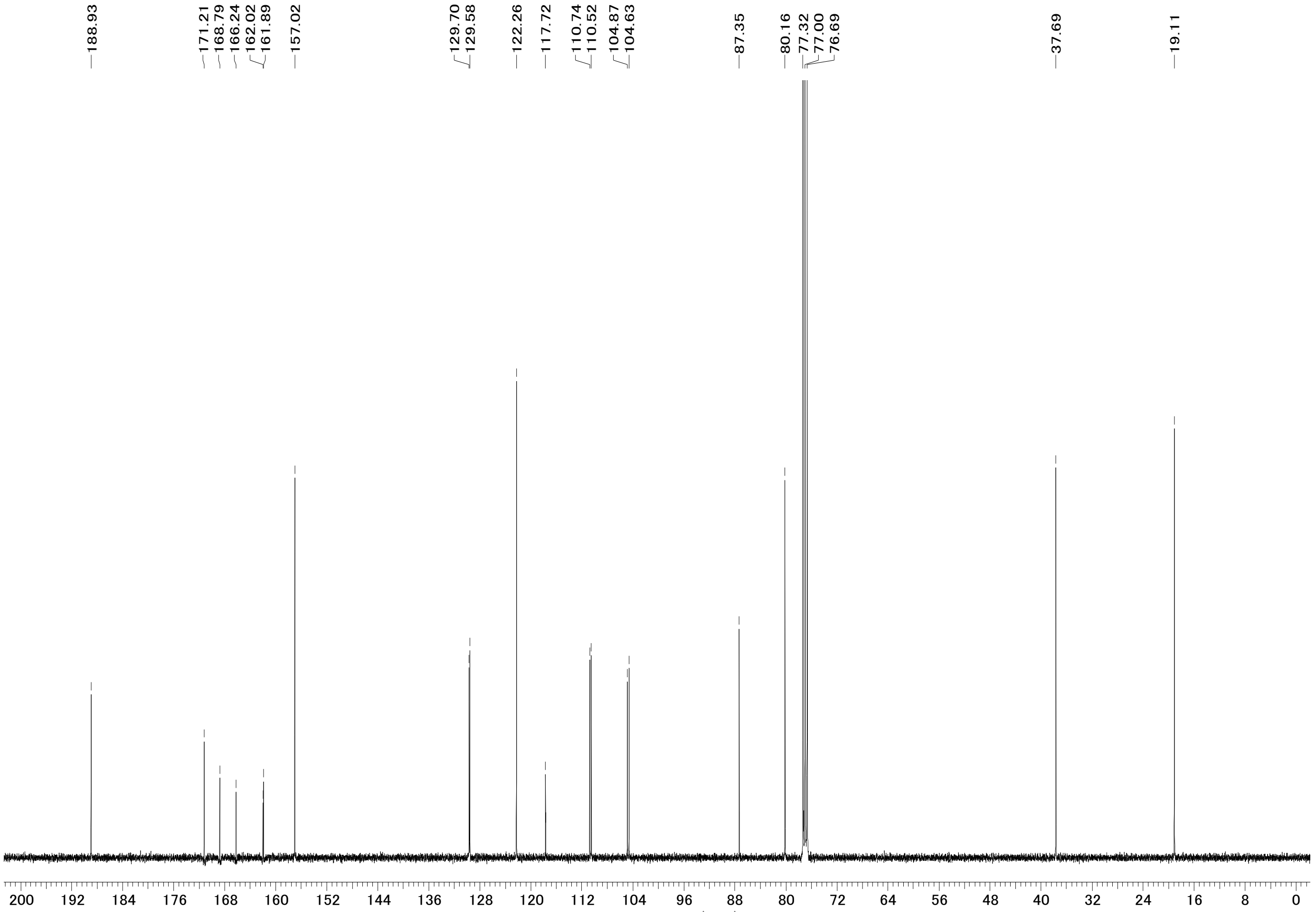
188.93
171.21
168.79
166.24
162.02
161.89
157.02
129.70
129.58
122.26
117.72
110.74
110.52
104.87
104.63
87.35
80.16
77.32
77.00
76.69
37.69
19.11
200 192 184 176 168 160 152 144 136 128 120 112 104 96 88 80 72 64 56 48 40 32 24 16 8 0

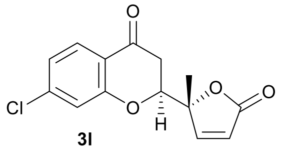

3l

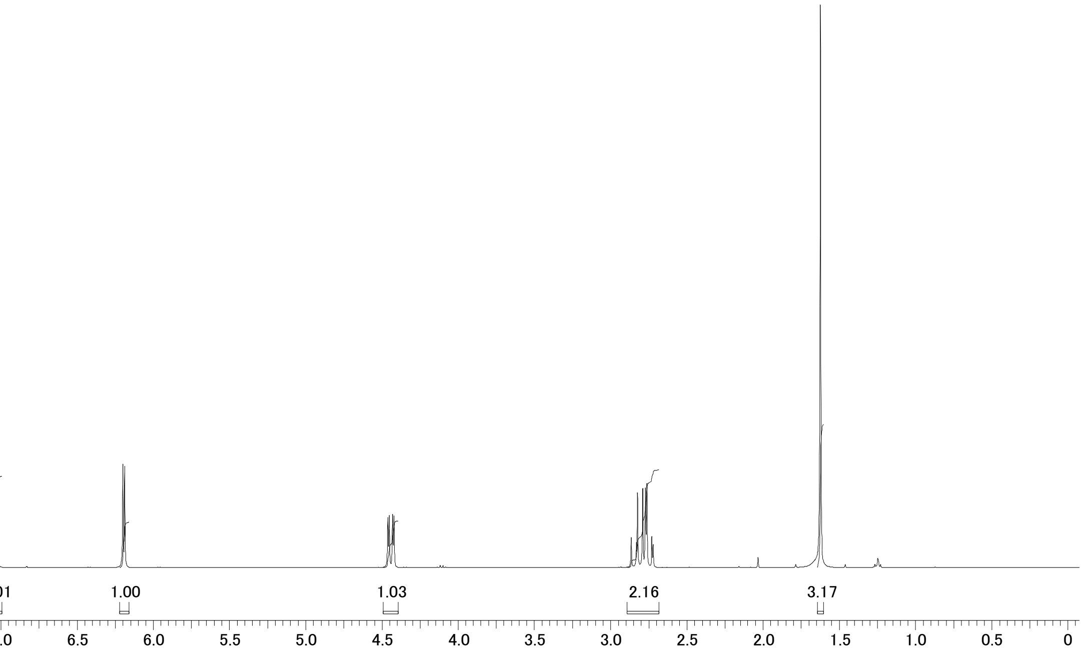
1.02 1.04 2.01 1.00 1.03 2.16 3.17
9.0 8.5 8.0 7.5 7.0 6.5 6.0 5.5 5.0 4.5 4.0 3.5 3.0 2.5 2.0 1.5 1.0 0.5 0

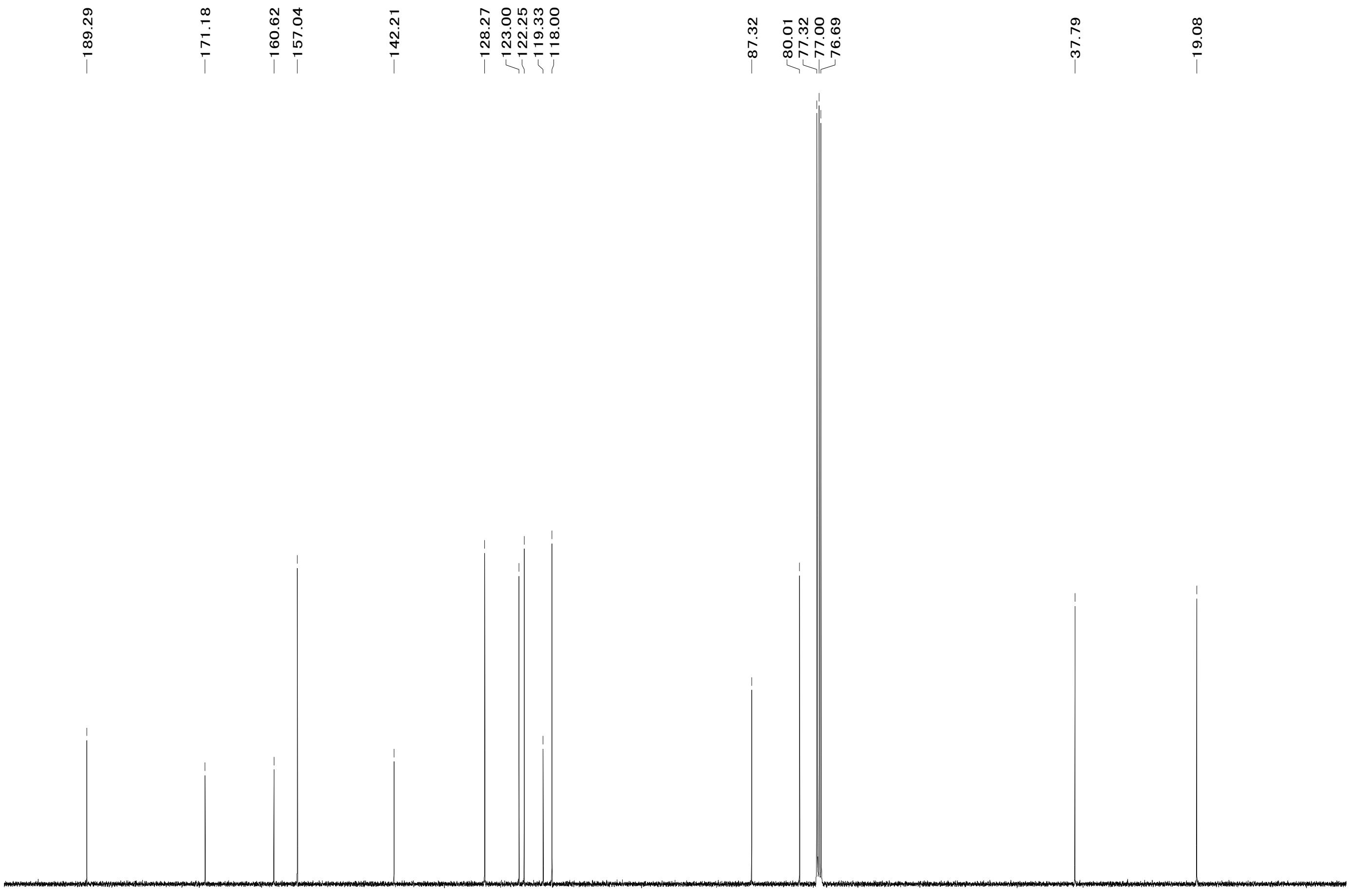
189.29
171.18
160.62
157.04
142.21
128.27
123.00
122.25
119.33
118.00
87.32
80.01
77.32
77.00
76.69
37.79
19.08
200 192 184 176 168 160 152 144 136 128 120 112 104 96 88 80 72 64 56 48 40 32 24 16 8 0

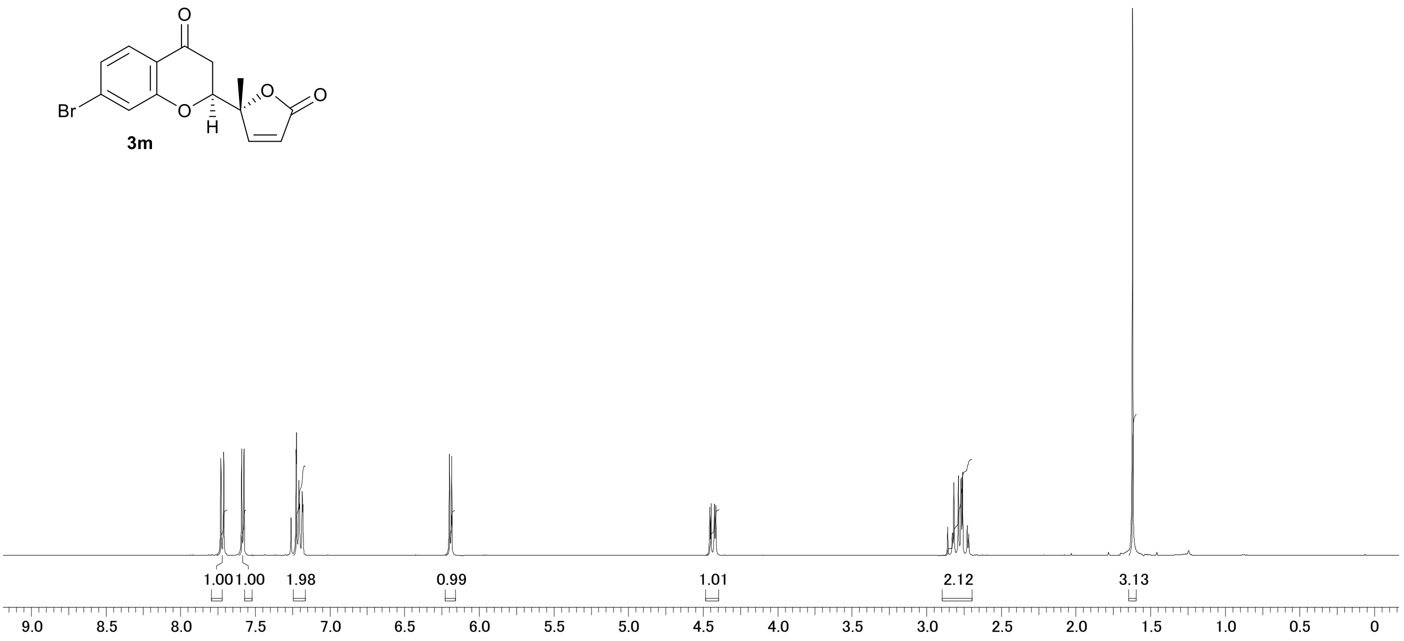
Br
O
O
H
O
O
1.00 1.00 1.98 0.99 1.01 2.12 3.13
9.0 8.5 8.0 7.5 7.0 6.5 6.0 5.5 5.0 4.5 4.0 3.5 3.0 2.5 2.0 1.5 1.0 0.5 0

3m

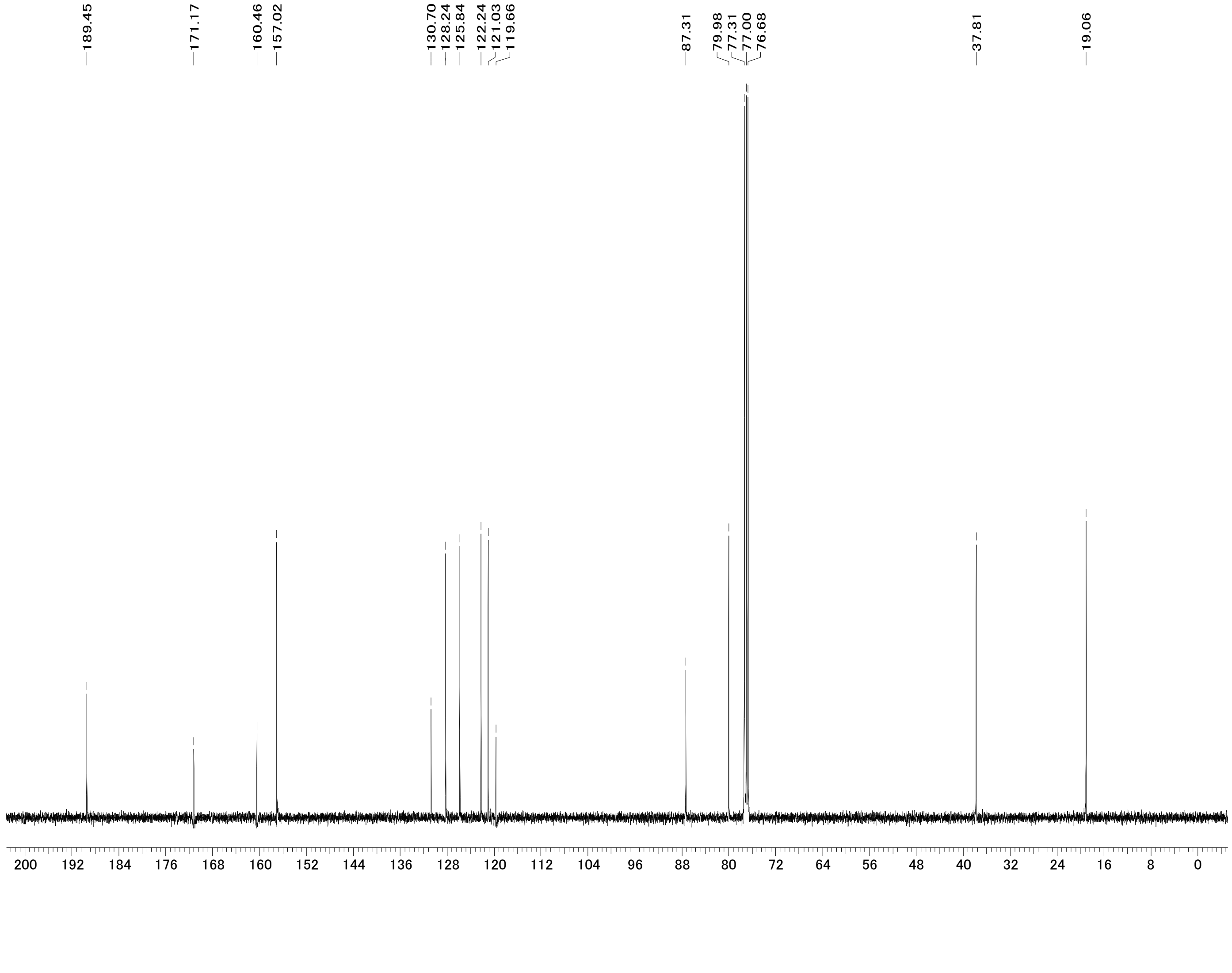
189.45
171.17
160.46
157.02
130.70
128.24
125.84
122.24
121.03
119.66
87.31
79.98
77.31
77.00
76.68
37.81
19.06
200 192 184 176 168 160 152 144 136 128 120 112 104 96 88 80 72 64 56 48 40 32 24 16 8 0

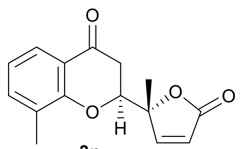

3n

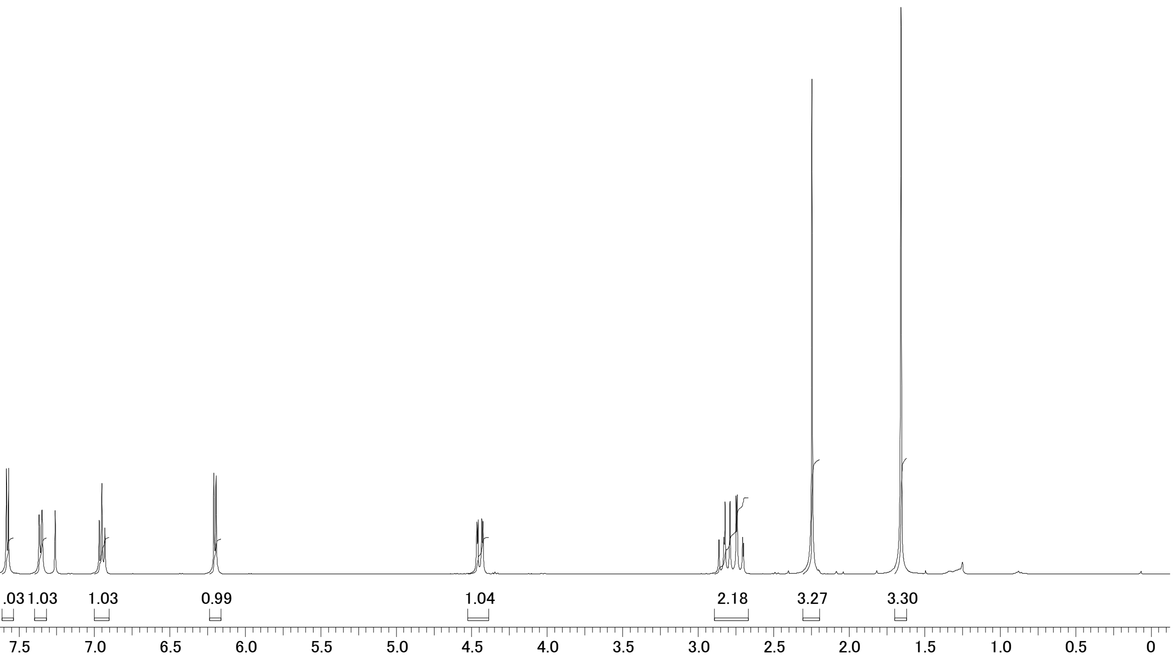
1.00 1.03 1.03 1.03 0.99 1.04 2.18 3.27 3.30
9.0 8.5 8.0 7.5 7.0 6.5 6.0 5.5 5.0 4.5 4.0 3.5 3.0 2.5 2.0 1.5 1.0 0.5 0

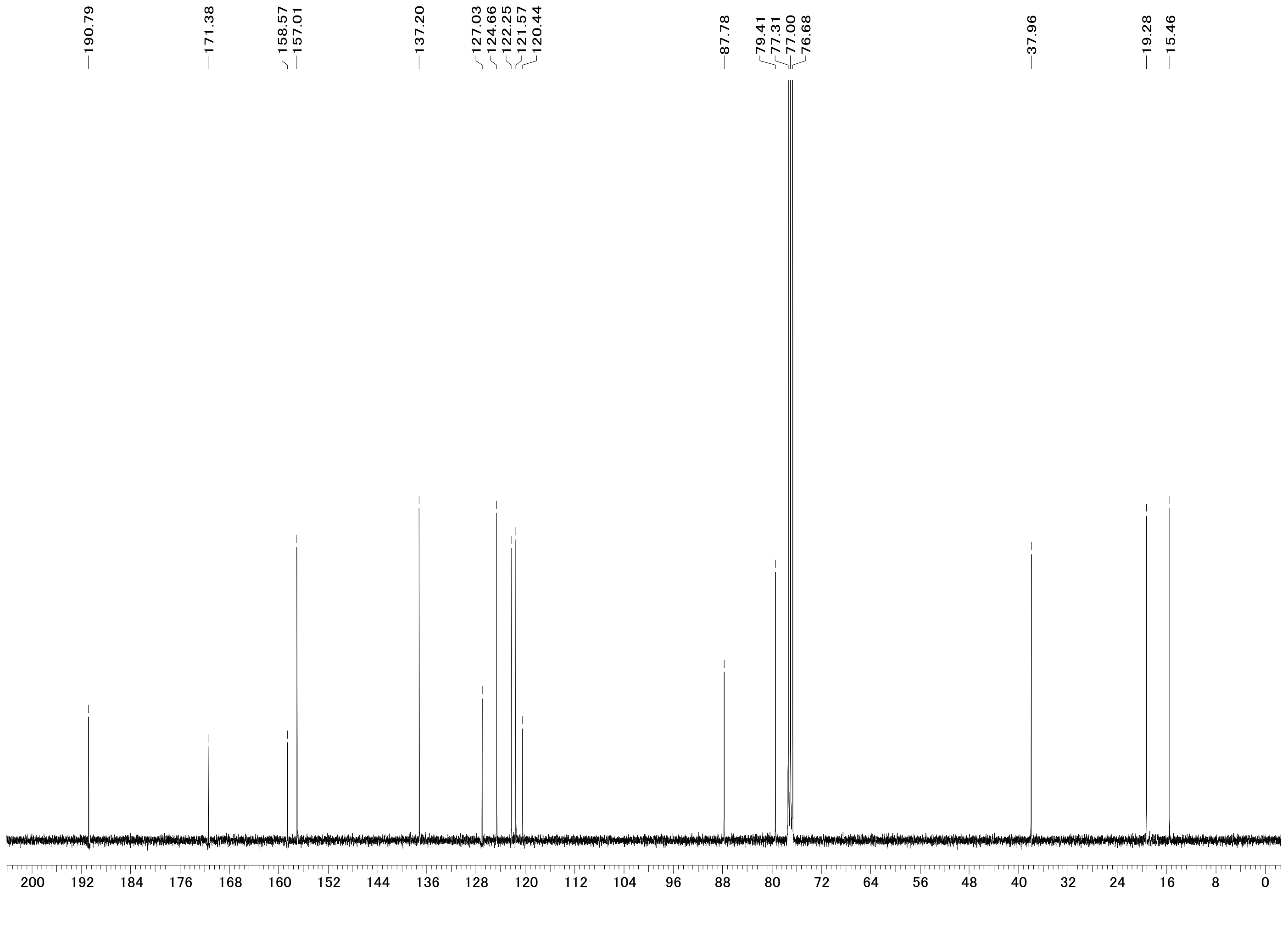
190.79
171.38
158.57
157.01
137.20
127.03
124.66
122.25
121.57
120.44
87.78
79.41
77.31
77.00
76.68
37.96
19.28
15.46
200 192 184 176 168 160 152 144 136 128 120 112 104 96 88 80 72 64 56 48 40 32 24 16 8 0

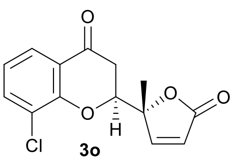

3o

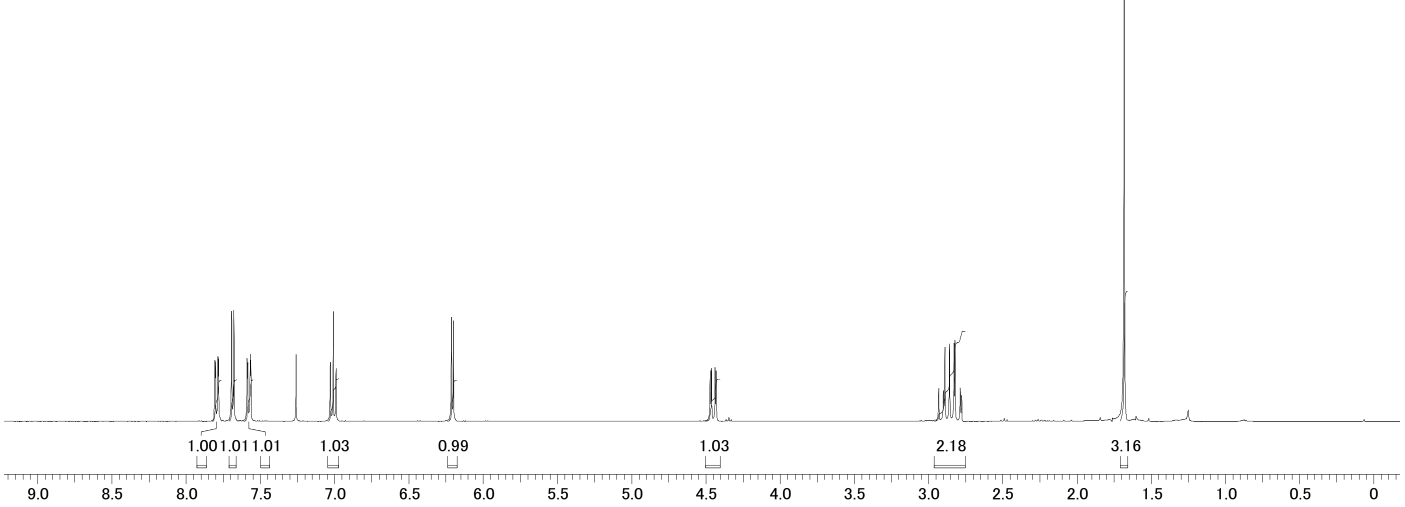
1.00
1.01
1.01
1.03
0.99
1.03
2.18
3.16
9.0 8.5 8.0 7.5 7.0 6.5 6.0 5.5 5.0 4.5 4.0 3.5 3.0 2.5 2.0 1.5 1.0 0.5 0

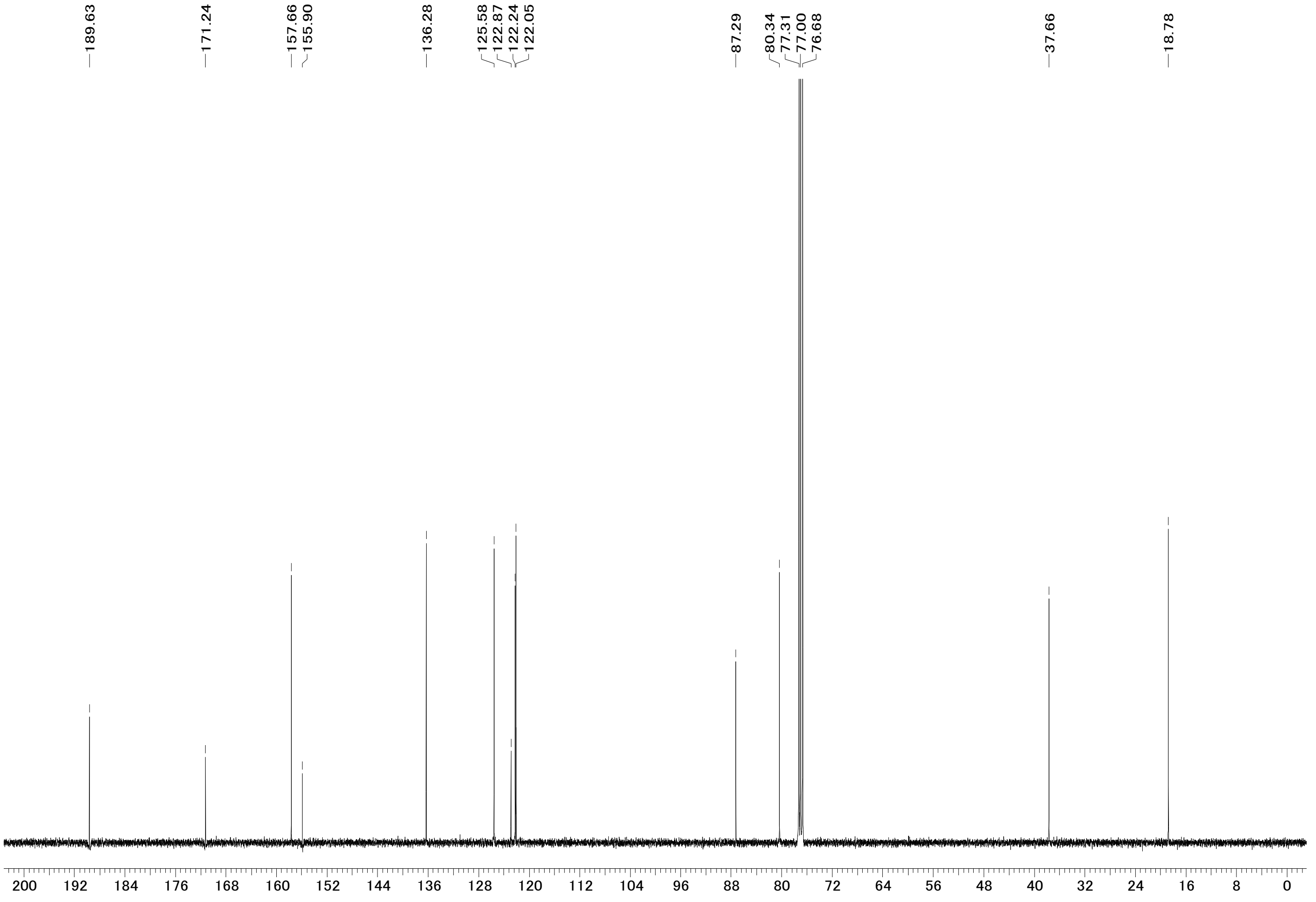
189.63
171.24
157.66
155.90
136.28
125.58
122.87
122.24
122.05
87.29
80.34
77.31
77.00
76.68
37.66
18.78
200 192 184 176 168 160 152 144 136 128 120 112 104 96 88 80 72 64 56 48 40 32 24 16 8 0

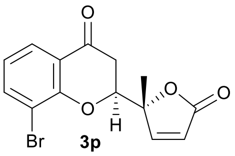
O
O
H
O
O
Br

3p

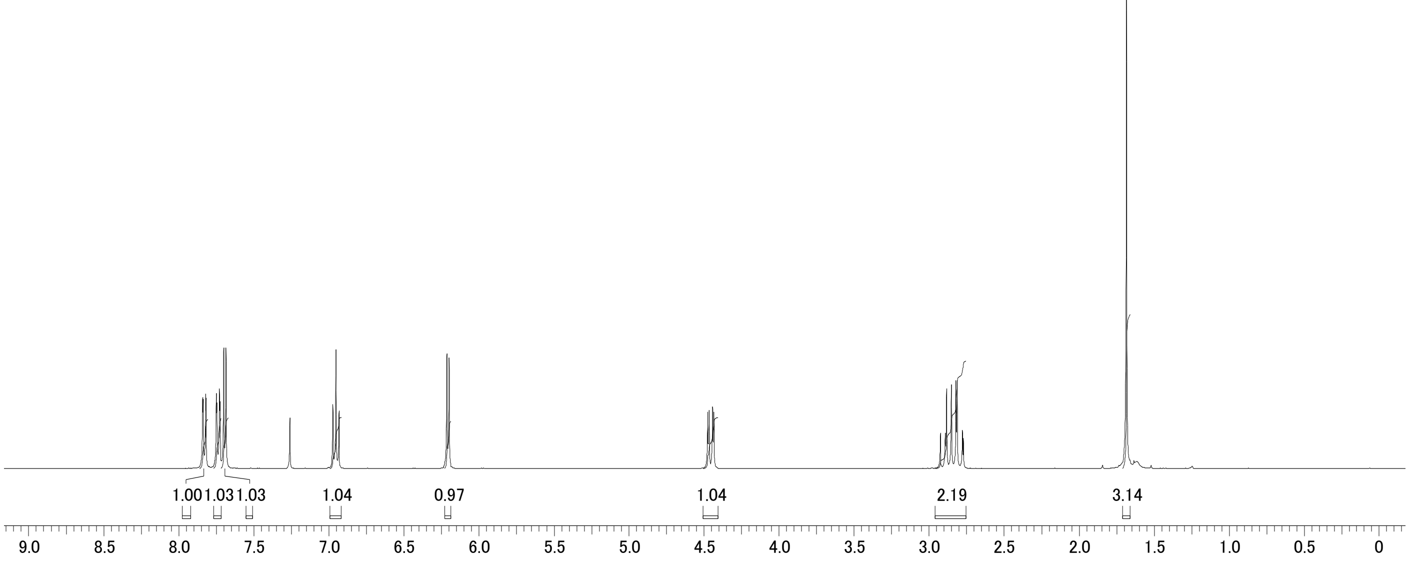
1.00 1.03 1.03
1.04
0.97
1.04
2.19
3.14
9.0 8.5 8.0 7.5 7.0 6.5 6.0 5.5 5.0 4.5 4.0 3.5 3.0 2.5 2.0 1.5 1.0 0.5 0

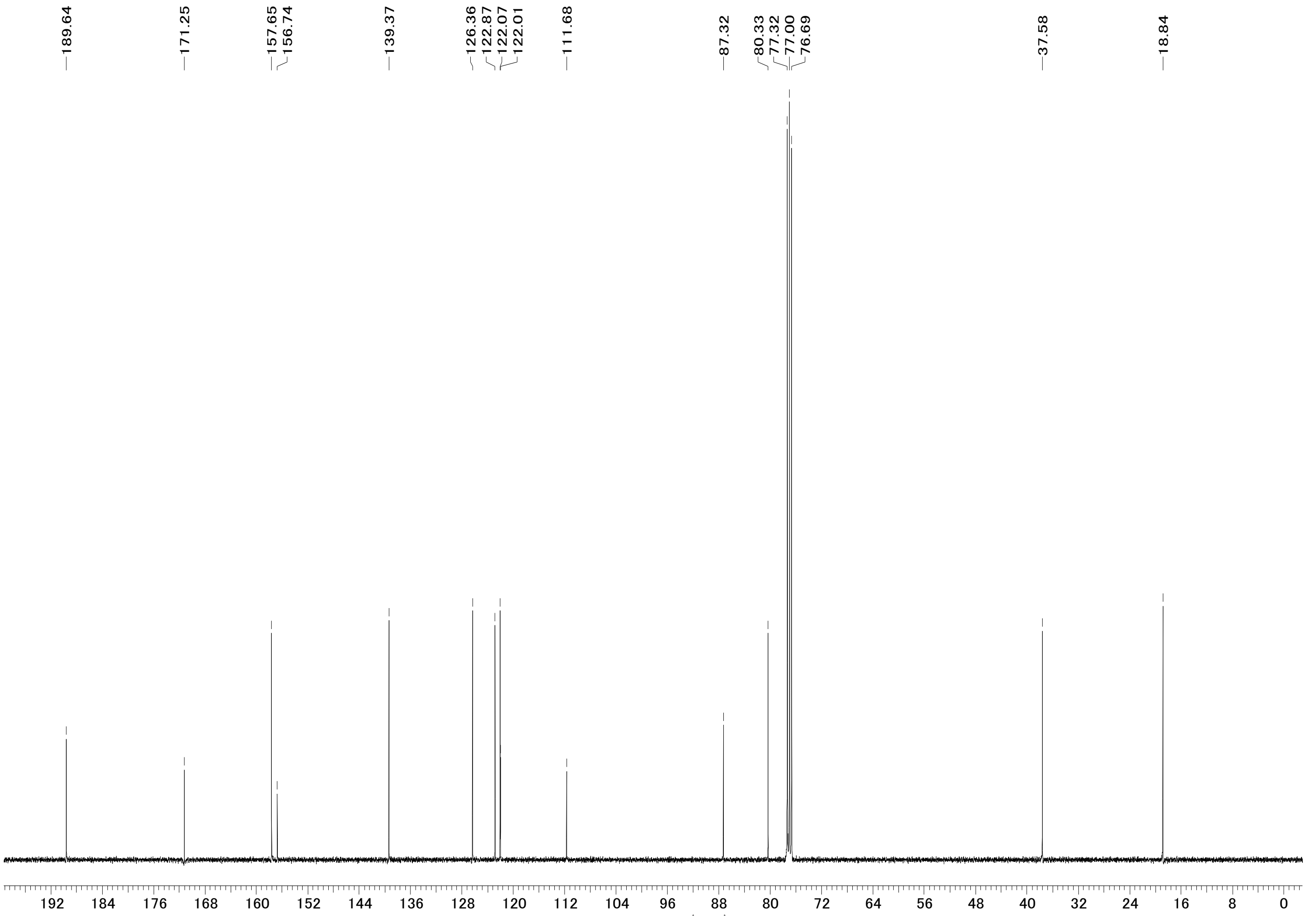
189.64
171.25
157.65
156.74
139.37
126.36
122.87
122.07
122.01
111.68
87.32
80.33
77.32
77.00
76.69
37.58
18.84
192 184 176 168 160 152 144 136 128 120 112 104 96 88 80 72 64 56 48 40 32 24 16 8 0

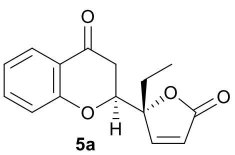
O
O
H
O
O

5a

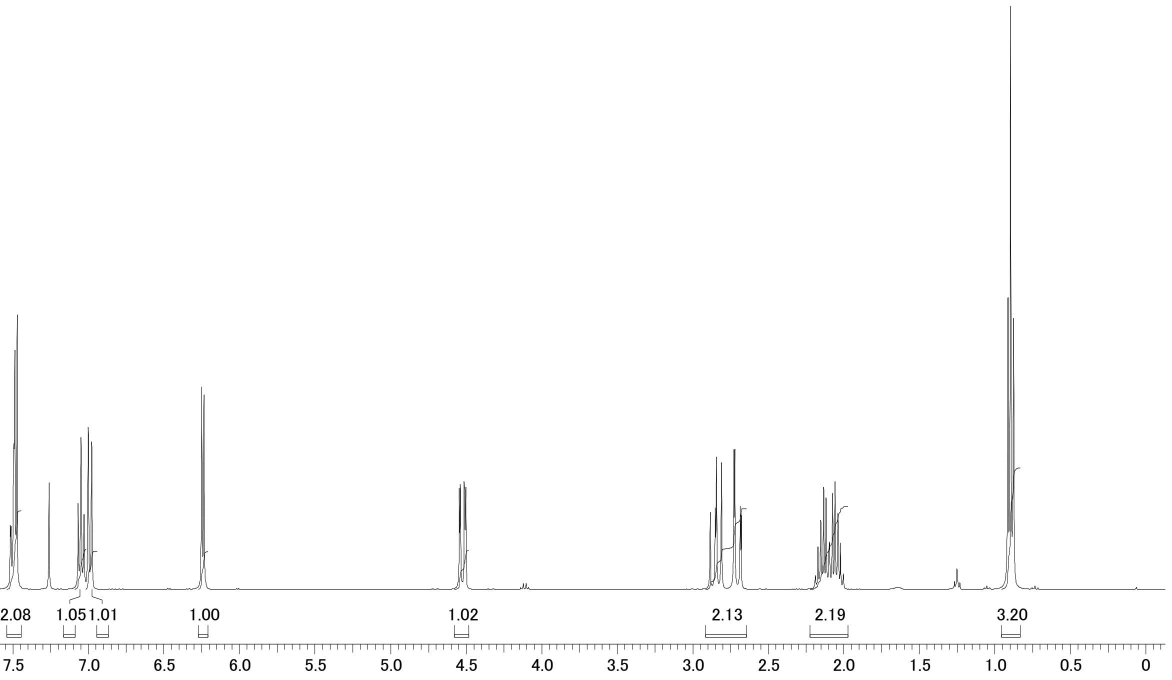
1.00 2.08 1.05 1.01 1.00 1.02 2.13 2.19 3.20
9.0 8.5 8.0 7.5 7.0 6.5 6.0 5.5 5.0 4.5 4.0 3.5 3.0 2.5 2.0 1.5 1.0 0.5 0

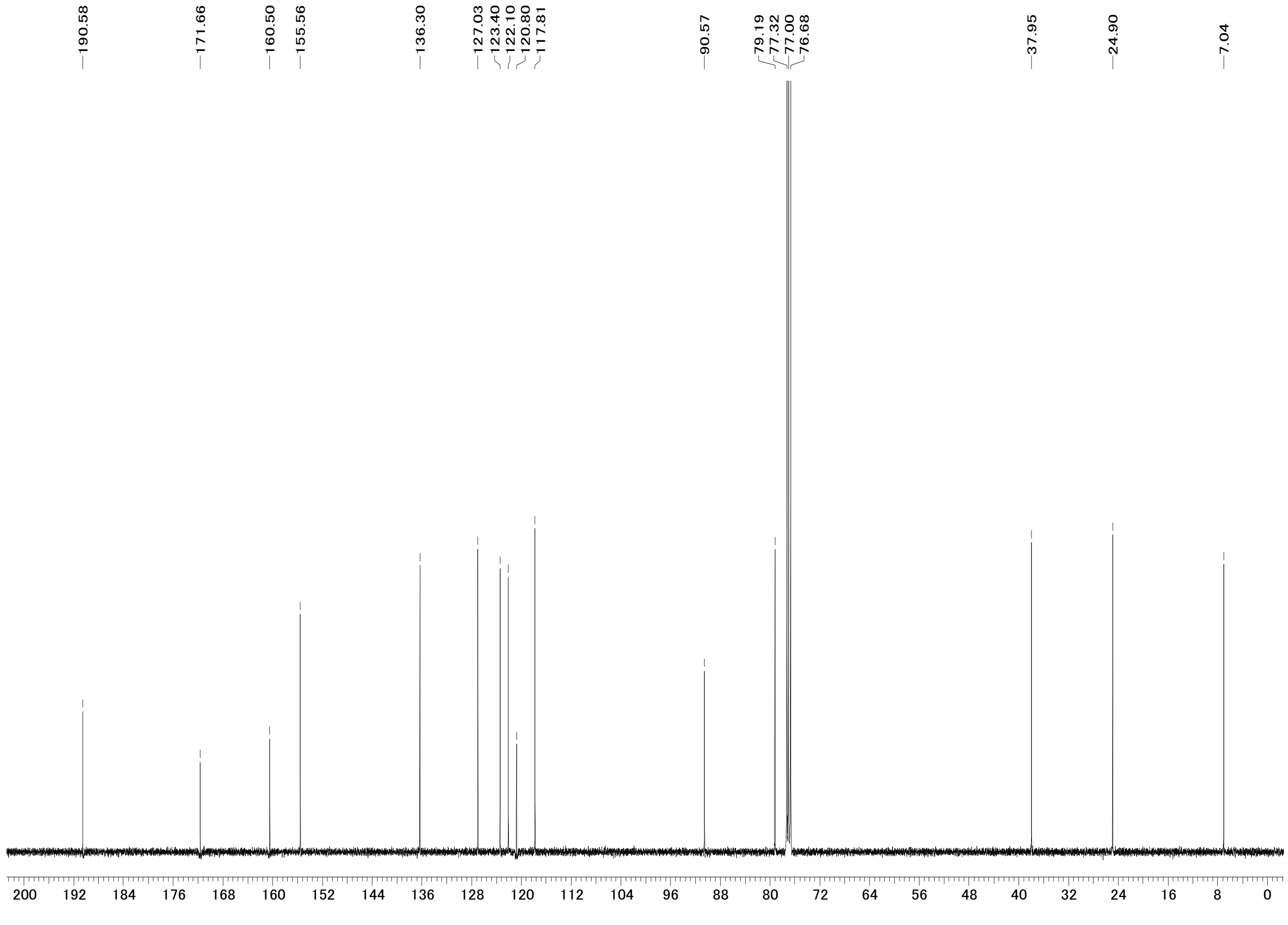
190.58
171.66
160.50
155.56
136.30
127.03
123.40
122.10
120.80
117.81
90.57
79.19
77.32
77.00
76.68
37.95
24.90
7.04
200 192 184 176 168 160 152 144 136 128 120 112 104 96 88 80 72 64 56 48 40 32 24 16 8 0

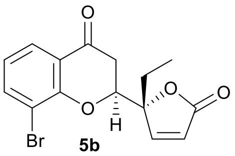

5b

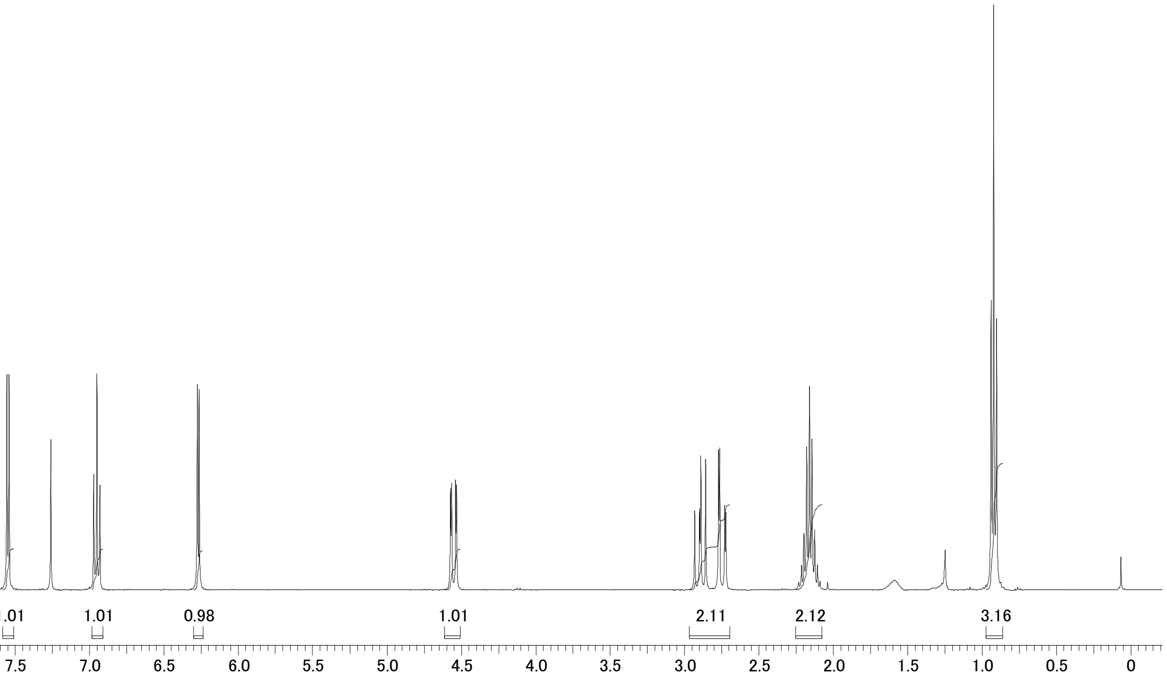

1.00
0.99
1.01
1.01
0.98
1.01
2.11
2.12
3.16
9.0 8.5 8.0 7.5 7.0 6.5 6.0 5.5 5.0 4.5 4.0 3.5 3.0 2.5 2.0 1.5 1.0 0.5 0

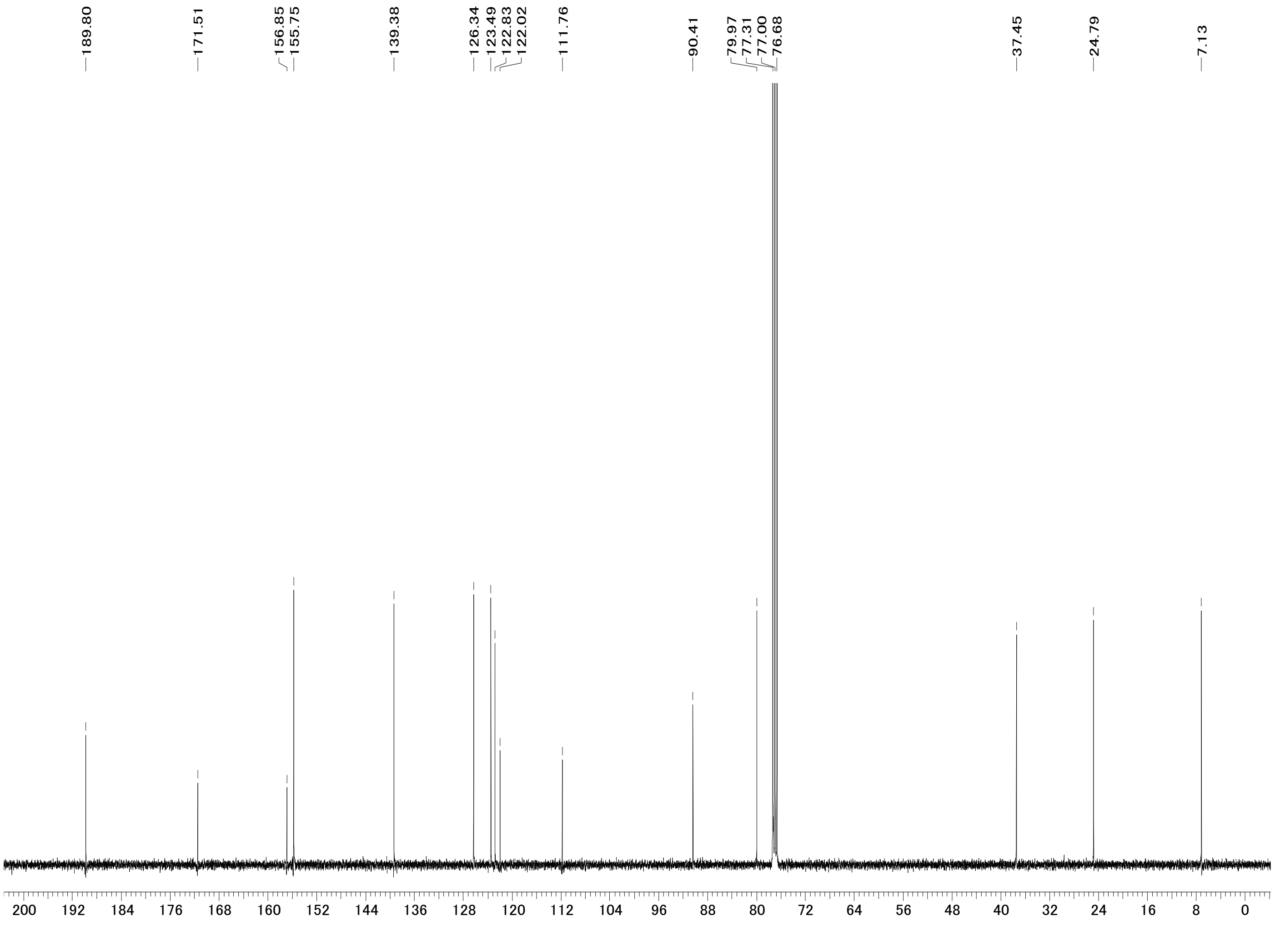

189.80
171.51
156.85
155.75
139.38
126.34
123.49
122.83
122.02
111.76
90.41
79.97
77.31
77.00
76.68
37.45
24.79
7.13
200 192 184 176 168 160 152 144 136 128 120 112 104 96 88 80 72 64 56 48 40 32 24 16 8 0
